# Global, regional, and national burden of low back pain in working-age population from 1990 to 2021 and projections for 2050

**DOI:** 10.3389/fpubh.2025.1559355

**Published:** 2025-04-24

**Authors:** Cifeng Zhang, Bing Lv, Qian Yi, Guicong Qiu, Fengling Wu

**Affiliations:** ^1^The First Affiliated Hospital of Jinan University, Guangzhou, China; ^2^The Fifth People's Hospital of Longgang District, Shenzhen, China

**Keywords:** low back pain, global burden of disease, incidence, prevalence, DALYs

## Abstract

**Background:**

Low back pain (LBP) is a leading cause of disability worldwide, especially among working-age group. This study evaluates the global, regional, and national burden of LBP among individuals aged 15–64 utilizing data from the Global Burden of Disease (GBD) 2021 study.

**Methods:**

We assessed trends in incidence, prevalence, and disability-adjusted life years (DALYs) for LBP from 1900 to 2021. Age-standardized rates (ASRs) were calculated, and joinpoint regression and decomposition analyses were used to identify key drivers. Future trends were projected through 2050.

**Results:**

The prevalence of LBP in working-age group has risen to 452.8 million cases globally, a 52.66% increase since 1990. South Asia reported the highest absolute number of cases, while Central and Eastern Europe showed the highest ASRs. Across all regions, women consistently exhibited higher incidence, prevalence, and DALYs than men. Decomposition analysis revealed that population growth was the main factor contributing to the rising burden. Projections indicate that LBP cases will continue increasing through 2050, particularly among women, although ASRs are expected to decline.

**Conclusion:**

The burden of LBP among working-age group is growing due to population expansion. Despite declining ASRs, substantial regional and gender disparities remain, highlighting the need for targeted public health strategies.

## 1 Introduction

Low back pain (LBP) is defined as discomfort in the posterior region of the back, extending from the lower margin of the 12th rib to the inferior gluteal folds, with or without referred pain in one or both lower limbs (1). The Global Burden of Disease (GBD) 2020 study estimated that the global prevalence of LBP affected 619 million individuals, constituting approximately one-tenth of the world’s population (2). The GBD 2021 study identified LBP as the predominant cause of years lived with disability (YLD), accounting for nearly 15.9 million disability-adjusted life years (DALYs) annually (3). LBP not only inflicts pain and dysfunction on individuals but also exerts a significant influence on the labor market, healthcare system, and the broader socioeconomic framework. As the global population ages and work patterns shift, the epidemiological trends and determinants of LBP are in a state of continuous flux (4).

The working-age population, as delineated by the World Health Organization (WHO) and the International Labour Organization (ILO) to include individuals aged 15 to 64, is pivotal to economic productivity (5). Individuals within the working-age group, face a higher risk of developing LBP due to the physical demands of their occupations. Sustained poor posture or repetitive physical tasks elevate the probability of this condition’s onset. Comprehensive understanding of the epidemiology of LBP in this demographic is essential for devising effective public health interventions and policies (6).

Prior research has explored the global burden of LBP, yet scant attention has been directed toward the working-age group cohort specifically. Previous studies utilizing GBD 2019 data have explored the global burden of LBP across various countries and age groups, yet scant research has specifically targeted the working-age group (7–10). Our study endeavors to offer a thorough and contemporary analysis of LBP within the working-age population, leveraging the latest GBD 2021 data alongside sophisticated analytical techniques. These include estimated annual percentage change (EAPC), joinpoint regression, decomposition analysis, and Bayesian ratio age-period-cohort (BAPC) modeling for projections.

## 2 Materials and methods

### 2.1 Data sources and study population

Data for this study were sourced from the GBD 2021 edition, accessed via the GBD Outcomes Tool on the Institute for Health Metrics and Evaluation (IHME) website^1^. The GBD 2021 edition offers estimates for 369 diseases, injuries, and 88 risk factors across 204 countries and territories, categorized into 21 regions. We specifically extracted LBP data pertaining to incidence, prevalence, and DALYs for the working-age group (11). The data is stratified by gender, age, socio-demographic index (SDI), geographical region, and country (12). The SDI is a composite measure reflecting a country’s socio-economic development, derived from indicators such as fertility rates, educational attainment, and per capita income (13). LBP is classified at the third level in the GBD 2021 system, and ICD codes are used for diagnostic purposes (Supplementary Table S1). Our analysis strictly followed the Guidelines for Accurate and Transparent Health Estimates Reporting (GATHER) (14) and the University of Washington’s Institutional Review Board (IRB) granted a waiver for informed consent, permitting access to the GBD data (15).

The working-age population, as defined by the Organisation for Economic Co-operation and Development (OECD) to include individuals aged 15–64 years, constitutes a pivotal demographic in global health research. This cohort represents a substantial segment of the workforce, contributing significantly to the economy and frequently encountering occupational risk factors that heighten the risk of developing LBP (16).

### 2.2 Statistical analyses

**Figure 1:**
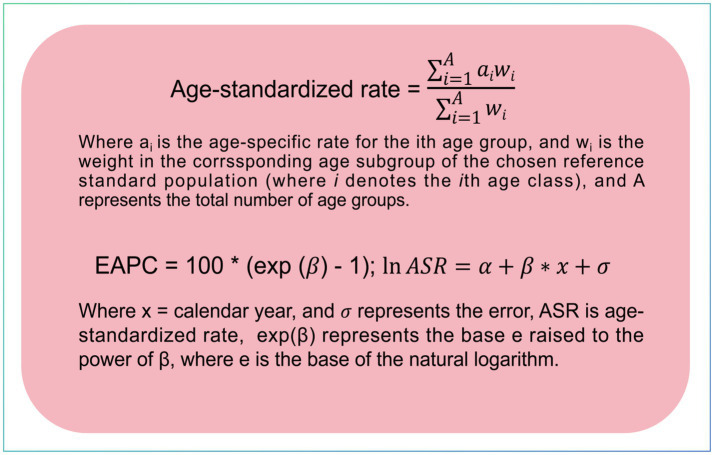
Equations for calculating age-standardized rates and estimated annual percentage change.

Age-standardized rates (ASRs) were employed to quantify the incidence, prevalence, and DALYs of LBP. These ASRs were calibrated to the global age distribution, facilitating comparisons across populations with varying age structures (17). To evaluate the trend in LBP, we determined the EAPC from these indicators, which was derived from a regression model where the natural logarithm of the ASR was regressed on the calendar year. The trend is considered increasing if both the EAPC and its lower 95% confidence interval (CI) are positive; decreasing if both the EAPC and its upper 95% CI are negative; and stable otherwise (18). The calculation formulas for ASR and EAPC are displayed in Figure 1. Spearman correlation was used to assess the correlation between SDI and ASR.

Joinpoint regression (version 5.0.2) was utilized to analyze the temporal trends in LBP from 1990 to 2021. This analytical approach identifies ‘junctures’ where trend changes occur and estimates the annual percentage change (APC) for each segment. A linear regression model served as the dependent variable in the analysis. The model accounted for both the zero-inflation and proportional variance using a Poisson distribution. The optimal number of breakpoints was ascertained based on the Bayesian Information Criterion (BIC) (19, 20). Trends were classified as increasing, decreasing, or stable based on the APC and its corresponding 95% CI. Decomposition analysis was employed to investigate the factors driving changes in DALYs attributed to LBP over time. We assessed the contributions of population growth, alterations in age structure, and epidemiological factors at both the global and regional levels (21, 22). We used the BAPC model with Integrated Nested Laplace Approximation (INLA) to forecast future trends in LBP from 2022 to 2050. Projections included the number of cases and ASR (23, 24). The combination of BAPC and INLA outperforms traditional Age-Period-Cohort (APC) models in terms of precision and convergence (25). All statistical analyses were conducted using R software (version 4.2.2).

## 3 Results

### 3.1 Global level

**Figure 2:**
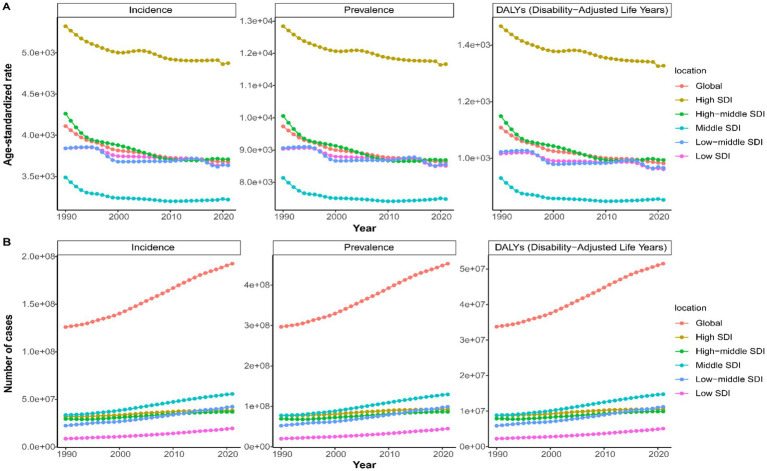
Age-standardized rate **(A)** and number **(B)** of incidence, prevalence, and DALYs change curves for LBP in working-age group by Global and SDI from 1990 to 2021.

Globally, there was a marked increase in the absolute numbers of incidence, prevalence, and DALYs for LBP from 1990 to 2021, while ASRs for these metrics displayed a decline (Figure 2). In 2021, the global burden of LBP among the working-age group was substantial, with 452.8 million cases [95% uncertainty interval (UI): 330.3–604.3], a 52.66% increase from the 296.64 million cases (95% UI: 215.2–395.4) reported in 1990. Despite this significant rise, the age-standardized prevalence rate (ASPR) showed a slight decrease, from 9,730.87 per 100,000 people (95% UI: 7,060.6–12,969.72) in 1990 to 8,631.79 per 100,000 people (95% UI: 6,295.99–11,517.11) in 2021 (Supplementary Figure S1). The EAPC for ASPR was −0.32 (95% CI: −0.36–−0.28) (Table 1). The global incidence of LBP reached 192.5 million cases (95% UI: 134.3–262.8) in 2021, a 52.9% increase from 125.92 million cases (95% UI: 87.5–172.2) in 1990. The age-standardized incidence rate (ASIR) declined from 4,111.22 per 100,000 people (95% UI: 2,862.47–5,618.98) in 1990 to 3,675.9 per 100,000 people (95% UI: 2,563.49–5,020.64) in 2021 (Supplementary Figure S1). Over the study period, the EAPC in ASIR was −0.3 (95% CI: −0.34–−0.26), indicating a consistent decrease in the incidence rate of LBP (Table 2). Additionally, LBP contributed to 51.5 million DALYs globally in 2021 (95% UI: 31.8–76.5), a 52.51% increase from 33.7 million DALYs (95% UI: 20.8 to 50.3) in 1990 (Supplementary Figure S1). Despite these increases, the age-standardized death rate (ASDR) showed a slight decline from 1990 to 2021, with a minimal change of −0.31% (95% CI: −0.35 to −0.27) (Table 3).

**Table 1:** The case number and ASR of prevalence of LBP in aged 15–64 between 1990 and 2021 by sex, SDI quintile and region, with EAPC from 1990 to 2021.

Characteristics	Number of cases in 1990 (95%UI)	ASPR (1/100,000) (95%UI)	Number of cases in 2021 (95%UI)	ASPR (1/100,000) (95%UI)	EAPC (95%CI)
Global	296,642,960 (215204490–395,405,566)	9730.87 (7060.6–12969.72)	452,846,791 (330333138–604,396,309)	8631.79 (6295.99–11517.11)	−0.32 (−0.36–−0.28)
Sex					
1 Female	181,779,425 (131887939–241,458,952)	12025.71 (8726.45–15969.16)	281,435,400 (205375959–373,723,566)	10742.5 (7838.24–14263.67)	−0.29 (−0.34–−0.24)
2 Male	114,863,535 (83052587–154,202,535)	7476.28 (5405.16–10040.19)	171,411,390 (124374955–230,170,260)	6528.2 (4737.01–8763.87)	−0.39 (−0.42–−0.36)
SDI region					
1 High SDI	77,274,769 (57105936–101,490,300)	12837.99 (9485.16–16863.9)	92,246,403 (70795946–118,421,405)	11662.1 (8953.29–14956.28)	−0.24 (−0.27–−0.21)
2 High-middle SDI	69,239,192 (50321775–92,184,033)	10056.01 (7309.23–13387.01)	86,579,542 (62634606–115,920,251)	8690.65 (6282.42–11633.22)	−0.39 (−0.46–−0.33)
3 Middle SDI	77,479,340 (55355152–104,614,900)	8135.4 (5812.78–10988.02)	129,464,382 (92484333–174,824,719)	7480.41 (5344.98–10095.9)	−0.17 (−0.22–−0.11)
4 Low-middle SDI	52,234,309 (37562830–70,111,395)	9060.35 (6515.15–12160.28)	99,061,649 (70836855–133,825,654)	8548.07 (6113.63–11548.96)	−0.18 (−0.22–−0.13)
5 Low SDI	20,069,793 (14473489–26,853,856)	9035.4 (6512.93–12086.16)	45,072,896 (32387947–60,627,236)	8508.7 (6113.34–11450.33)	−0.19 (−0.22–−0.17)
GBD region					
1 Andean Latin America	1,300,086 (929913–1,758,658)	6783.28 (4847.1–9201.32)	2,847,094 (2047731–3,835,421)	6769.81 (4868.75–9120.79)	0.01 (−0.01–0.05)
2 Australasia	2,066,723 (1526064–2,717,767)	15311.4 (11303.92–20142.97)	3,008,647 (2178172–4,015,827)	14022.97 (10158.56–18713.02)	−0.19 (−0.22–−0.15)
3 Caribbean	1,445,197 (1034694–1,945,278)	7249.11 (5188.3–9765.05)	2,274,842 (1652979–3,032,091)	7120.73 (5172.78–9490.37)	−0.01 (−0.02–0)
4 Central Asia	3,907,378 (2836612–5,220,358)	10462.28 (7601.75–13968.93)	6,593,807 (4753671–8,821,861)	10433.68 (7521.68–13964.35)	0 (0–0.01)
5 Central Europe	13,531,238 (9943671–17,789,506)	15661.73 (11500.55–20604.99)	13,054,958 (9520426–17,233,576)	15227.76 (11096.56–20107.97)	−0.1 (−0.11–−0.09)
6 Central Latin America	7,601,306 (5436561–10,208,604)	9041.69 (6465.48–12166.94)	15,531,012 (11181554–20,913,070)	9208.33 (6629.87–12397.47)	0.05 (0–0.1)
7 Central Sub-Saharan Africa	2,151,323 (1542370–2,914,873)	9015.2 (6458.16–12207.15)	5,558,525 (3969417–7,531,464)	8692.87 (6208.43–11765.93)	−0.14 (−0.17–−0.11)
8 East Asia	56,107,998 (39955974–76,098,904)	7585.01 (5405.55–10287.25)	71,737,362 (51716562–96,683,286)	6162.11 (4438.82–8301.64)	−0.47 (−0.58–−0.35)
9 Eastern Europe	21,105,700 (15366990–28,015,849)	12962.08 (9429.98–17214.43)	19,997,331 (14568950–26,570,220)	12472.83 (9071.65–16602.91)	−0.04 (−0.06–−0.02)
10 Eastern Sub-Saharan Africa	6,991,696 (5032759–9,404,430)	8898.99 (6407.86–11963.5)	16,718,989 (12008068–22,526,836)	8563.98 (6146.19–11554.81)	−0.14 (−0.15–−0.13)
11 High-income Asia Pacific	16,672,536 (12109033–22,162,508)	13425.47 (9753.38–17843.67)	16,030,957 (11581002–21,474,373)	12186.32 (8805.16–16296.18)	−0.26 (−0.28–−0.24)
12 High-income North America	26,478,416 (19646880–34,549,504)	14129.53 (10479.51–18448.14)	33,164,835 (27035294–40,447,087)	12785.06 (10424.1–15587.74)	−0.15 (−0.22–−0.08)
13 North Africa and Middle East	17,716,053 (13039690–23,431,481)	10370.17 (7633.7–13728.31)	40,340,535 (29102857–54,118,941)	10073.4 (7260.21–13526.63)	−0.06 (−0.08–−0.04)
14 Oceania	234,275 (166874–316,932)	7388.76 (5260.68–10001.61)	563,433 (400515–763,915)	7335.42 (5213.45–9949.6)	0 (−0.01–0.02)
15 South Asia	50,035,142 (35872880–67,251,932)	9010.41 (6462.07–12103.37)	93,413,074 (66617610–126,303,904)	8087.09 (5770.03–10931.36)	−0.34 (−0.44–−0.25)
16 Southeast Asia	16,557,288 (11949773–22,279,064)	6794.66 (4901.2–9149.99)	32,662,570 (23304570–44,205,634)	6727.57 (4800.7–9104.43)	−0.01 (−0.02–0)
17 Southern Latin America	3,572,163 (2591771–4,762,739)	11917.65 (8647.11–15887.97)	5,507,152 (3981218–7,358,567)	11845.21 (8563.53–15826.19)	−0.01 (−0.05–0.02)
18 Southern Sub-Saharan Africa	1,878,427 (1347408–2,537,203)	7625.26 (5473.4–10297.93)	3,525,900 (2514867–4,762,265)	7275.1 (5189.33–9831.34)	−0.12 (−0.13−−0.11)
19 Tropical Latin America	9,255,776 (6656614–12,447,624)	10957.53 (7880.39–14737.98)	17,761,409 (12793784–23,887,660)	11103.58 (7996.38–14932.18)	0.01 (0–0.04)
20 Western Europe	31,201,039 (23130783–40,998,981)	11619.5 (8613.51–15261.7)	35,040,339 (25334206–46,899,586)	11146.53 (8064.98–14895.34)	−0.1 (−0.11–−0.08)
21 Western Sub-Saharan Africa	6,833,199 (4930867–9,179,879)	8125.47 (5861.68–10921.62)	17,514,020 (12576483–23,620,977)	7985.19 (5736.27–10774.41)	−0.06 (−0.1–−0.02)

ASPR, age-standardized prevalence rate; EAPC, estimated annual percentage change; SDI, sociodemographic index; CI, confidence interval; UI, uncertainty interval.

**Table 2:** The case number and ASR of incidence of LBP in aged 15–64 between 1990 and 2021 by sex, SDI quintile and region, with EAPC from 1990 to 2021.

Characteristics	Cases in 1990 (95%UI)	ASIR (1/100,000) (95%UI)	Cases in 2021 (95%UI)	ASIR (1/100,000) (95%UI)	EAPC (95%CI)
Global	125,925,385 (87544428–172,267,414)	4111.22 (2862.47–5618.98)	192,550,188 (134315720–262,878,367)	3675.94 (2563.49–5020.64)	−0.3 (−0.34–−0.26)
Sex					
1 Female	75,951,023 (53026751–103,695,789)	5000.79 (3497.67–6823.17)	118,418,188 (82947248–161,150,943)	4528.97 (3170.7–6166.81)	−0.25 (−0.3–−0.2)
2 Male	49,974,361 (34573334–68,574,620)	3,238 (2242.53–4436.53)	74,132,000 (51443798–101,390,522)	2826.69 (1961.14–3866.76)	−0.39 (−0.42–−0.36)
SDI region					
1 High SDI	31,996,276 (22636746–43,257,100)	5322.54 (3766.51–7194.25)	38,264,233 (27840059–50,615,316)	4874.22 (3547.7–6454.88)	−0.22 (−0.25–−0.19)
2 High-middle SDI	29,407,862 (20509990–40,183,442)	4262.32 (2976.47–5821.91)	36,778,634 (25568300–50,289,192)	3708.61 (2574.68–5080.52)	−0.37 (−0.43–−0.31)
3 Middle SDI	33,470,147 (22933114–46,153,891)	3488.39 (2394.66–4802.16)	55,606,595 (38259999–76,458,930)	3220.22 (2214.56–4429.84)	−0.17 (−0.22–−0.12)
4 Low-middle SDI	22,304,333 (15371539–30,651,219)	3841.45 (2651.7–5272.34)	42,279,629 (29044383–58,279,689)	3635.29 (2500.23–5004.14)	−0.17 (−0.21–−0.13)
5 Low SDI	8,604,821 (5940659–11,821,306)	3841.06 (2658.9–5265.77)	19,445,994 (13368207–26,821,070)	3637.29 (2506.24–5003.85)	−0.18 (−0.2–−0.16)
GBD region					
1 Andean Latin America	570,301 (392916–785,348)	2956.53 (2038.3–4070.28)	1,246,659 (863571–1,711,670)	2960.96 (2051.98–4063.38)	0.02 (0–0.05)
2 Australasia	842,795 (596044–1,136,169)	6243.65 (4414.83–8412.8)	1,235,552 (861674–1,678,056)	5797.57 (4045.11–7884.31)	−0.15 (−0.17–−0.12)
3 Caribbean	627,518 (429017–864,096)	3132.58 (2143.38–4312.13)	984,506 (686699–1,344,506)	3087.22 (2153.37–4217.85)	−0.02 (−0.03–−0.01)
4 Central Asia	1,687,686 (1172570–2,313,336)	4493.67 (3133.27–6150.59)	2,829,579 (1962110–3,876,012)	4471.61 (3101.44–6124.53)	0 (−0.01–0)
5 Central Europe	5,398,459 (3805760–7,315,417)	6265.36 (4412.53–8499.08)	5,212,380 (3669280–7,056,409)	6138.77 (4310.43–8326.87)	−0.07 (−0.08–−0.07)
6 Central Latin America	3,224,029 (2211591–4,433,405)	3796.38 (2606.12–5212.97)	6,506,259 (4485666–8,933,634)	3858.83 (2660.46–5298.81)	0.04 (0–0.09)
7 Central Sub-Saharan Africa	932,231 (640581–1,287,702)	3,872 (2667.85–5337.34)	2,414,165 (1653161–3,336,430)	3742.97 (2568.38–5158.27)	−0.13 (−0.15–−0.1)
8 East Asia	24,527,075 (16743945–33,961,801)	3293.75 (2252.57–4551.58)	31,262,886 (21738588–42,798,228)	2698.83 (1875.11–3703.57)	−0.46 (−0.56–−0.35)
9 Eastern Europe	8,760,256 (6127502–11,931,222)	5397.21 (3777.18–7364.93)	8,331,610 (5841544–11,321,574)	5227.55 (3652.63–7126.17)	−0.03 (−0.05–−0.01)
10 Eastern Sub-Saharan Africa	3,036,652 (2088958–4,170,944)	3822.05 (2638.87–5234.76)	7,272,284 (4996304–10,035,640)	3684.2 (2538.58–5072.21)	−0.13 (−0.14–−0.12)
11 High-income Asia Pacific	6,865,578 (4801616–9,341,969)	5544.01 (3873.49–7549.22)	6,616,781 (4615567–9,015,050)	5081.45 (3540.42–6935.53)	−0.22 (−0.24–−0.19)
12 High-income North America	10,999,385 (7810364–14,831,262)	5866.93 (4166.93–7904.89)	13,781,072 (10424484–17,533,208)	5356.04 (4053.7–6816.87)	−0.15 (−0.22–−0.08)
13 North Africa and Middle East	7,376,602 (5177808–10,022,480)	4301.16 (3020.35–5845.58)	16,809,500 (11598307–23,073,766)	4196.95 (2893.73–5756.15)	−0.05 (−0.07–−0.04)
14 Oceania	102,670 (70401–142,293)	3203.98 (2199.76–4426.72)	245,452 (167332–337,938)	3176.14 (2169.21–4364.85)	−0.01 (−0.02–0)
15 South Asia	21,481,714 (14785843–29,571,432)	3840.14 (2647.79–5276.62)	40,315,604 (27644486–55,764,381)	3477.54 (2387.3–4803.06)	−0.32 (−0.41–−0.23)
16 Southeast Asia	7,229,000 (4982187–9,939,229)	2936.47 (2026.73–4032.26)	14,012,052 (9593895–19,337,440)	2891.39 (1979.05–3991.07)	−0.03 (−0.04–−0.02)
17 Southern Latin America	1,517,508 (1055478–2,066,344)	5052.73 (3514.85–6879.39)	2,321,978 (1617674–3,170,624)	5009.77 (3489.66–6842.56)	−0.01 (−0.04–0.03)
18 Southern Sub-Saharan Africa	824,736 (567621–1,137,008)	3318.51 (2291.65–4564.45)	1,539,763 (1058908–2,121,892)	3165.84 (2178.92–4355.8)	−0.13 (−0.13–−0.12)
19 Tropical Latin America	3,874,493 (2665869–5,320,599)	4554.67 (3138.3–6251.17)	7,297,263 (5053407–10,012,452)	4569.79 (3163.13–6269.4)	0 (−0.02–0.01)
20 Western Europe	13,054,611 (9258096–17,573,161)	4873.63 (3457.96–6564.54)	14,664,075 (10187235–20,015,546)	4690.28 (3255.5–6414.85)	−0.07 (−0.1–−0.05)
21 Western Sub-Saharan Africa	2,992,087 (2065043–4,109,687)	3526.15 (2439.82–4833.2)	7,650,770 (5257615–10,576,423)	3455.93 (2381.02–4763.85)	−0.07 (−0.11–−0.03)

ASIR, age-standardized incidence rate; EAPC, estimated annual percentage change; SDI, sociodemographic index; CI, confidence interval; UI, uncertainty interval.

**Table 3:** The case number and ASR of DALYs of LBP in aged 15–64 between 1990 and 2021 by sex, SDI quintile and region, with EAPC from 1990 to 2021.

Characteristics	Number of cases in 1990 (95%UI)	ASDR (1/100,000) (95%UI)	Number of cases in 2021 (95%UI)	ASDR (1/100,000) (95%UI)	EAPC (95%CI)
Global	33,774,033 (20852535–50,306,970)	1108.27 (685.54–1650.01)	51,510,153 (31892638–76,590,376)	981.79 (607.53–1460.21)	−0.31 (−0.35–−0.27)
Sex					
1 Female	20,561,461 (12731279–30,601,305)	1360.81 (844.12–2024.69)	31,774,138 (19714694–47,167,005)	1212.77 (751.83–1800.97)	−0.28 (−0.33–−0.23)
2 Male	13,212,572 (8113814–19,753,220)	860.15 (529.13–1285.73)	19,736,015 (12181688–29,520,121)	751.57 (463.75–1124.25)	−0.38 (−0.41–−0.35)
SDI region					
1 High SDI	8,829,101 (5518929–13,072,804)	1466.26 (916.52–2171.67)	10,497,420 (6753939–15,249,470)	1326.75 (851.47–1927.6)	−0.25 (−0.28–−0.22)
2 High-middle SDI	7,906,269 (4888223–11,755,339)	1148.57 (710.88–1708.12)	9,903,186 (6109710–14,795,755)	993.9 (611.71–1486.47)	−0.38 (−0.44–−0.31)
3 Middle SDI	8,851,163 (5394085–13,267,367)	929.84 (568.39–1392.81)	14,767,555 (9043438–22,110,951)	852.95 (521.89–1277.42)	−0.17 (−0.22–−0.11)
4 Low-middle SDI	5,892,534 (3625718–8,802,658)	1022.25 (631.09–1525.7)	11,200,732 (6832626–16,825,861)	966.03 (590.51–1450.43)	−0.16 (−0.21–−0.11)
5 Low SDI	2,255,601 (1388544–3,367,474)	1016.14 (627.77–1515.38)	5,093,147 (3119142–7,613,299)	961.4 (591.3–1435.42)	−0.17 (−0.19–−0.14)
GBD region					
1 Andean Latin America	148,355 (90463–223,531)	775.05 (473.94–1167.22)	324,878 (198941–485,611)	772.19 (473.19–1153.65)	0.02 (−0.01–0.05)
2 Australasia	234,269 (146185–347,022)	1735.23 (1083.65–2570.67)	342,010 (212337–511,963)	1592.48 (988.17–2384.24)	−0.18 (−0.22–−0.15)
3 Caribbean	164,502 (100238–247,134)	825.98 (504.04–1240.21)	258,135 (160206–383,823)	808 (501.11–1201.51)	−0.01 (−0.03–0)
4 Central Asia	446,427 (273835–665,486)	1196.27 (735.69–1780.77)	753,992 (465989–1,122,242)	1192.54 (737.13–1775.11)	0.01 (0–0.02)
5 Central Europe	1,540,921 (962984–2,283,177)	1784.85 (1114.6–2647.3)	1,490,925 (929268–2,212,244)	1740.57 (1082.21–2583.67)	−0.08 (−0.09–−0.07)
6 Central Latin America	864,199 (526570–1,296,362)	1028.51 (628.56–1542.01)	1,764,319 (1084620–2,636,778)	1046.02 (642.98–1563.09)	0.05 (0–0.09)
7 Central Sub-Saharan Africa	240,224 (147050–362,449)	1007.72 (618.73–1521.2)	626,611 (383324–944,565)	980.15 (602.58–1473.8)	−0.1 (−0.13–−0.08)
8 East Asia	6,471,913 (3924938–9,746,738)	875.13 (532.15–1317.52)	8,289,483 (5099856–12,445,169)	712 (436.87–1070.32)	−0.46 (−0.57–−0.35)
9 Eastern Europe	2,386,881 (1482552–3,554,559)	1467.87 (911.09–2187.78)	2,260,500 (1411454–3,357,021)	1412.61 (878.28–2100.92)	−0.02 (−0.05–0)
10 Eastern Sub-Saharan Africa	788,361 (483789–1,177,574)	1004.77 (619.82–1498.56)	1,895,082 (1158297–2,835,843)	970.88 (596.43–1450.79)	−0.11 (−0.11–−0.1)
11 High-income Asia Pacific	1,920,757 (1185510–2,869,983)	1546.51 (953.7–2311.5)	1,850,364 (1143256–2,766,942)	1405.17 (865.81–2100.93)	−0.25 (−0.28–−0.23)
12 High-income North America	3,013,766 (1894926–4,439,691)	1607.06 (1011.2–2368.23)	3,731,787 (2501266–5,212,688)	1439.98 (962.57–2012.81)	−0.17 (−0.25–−0.1)
13 North Africa and Middle East	2,009,988 (1251401–2,968,344)	1178.56 (735.66–1739.58)	4,571,384 (2806526–6,841,897)	1140.42 (700.56–1706.47)	−0.07 (−0.09–−0.05)
14 Oceania	26,704 (16243–40,154)	842.39 (513.83–1263.9)	64,292 (38915–96,481)	836.26 (507.57–1254.44)	0 (−0.01–0.02)
15 South Asia	5,625,074 (3460446–8,415,807)	1012.44 (625.04–1512.91)	10,522,771 (6426627–15,827,222)	910.35 (557.04–1368.62)	−0.33 (−0.42–−0.23)
16 Southeast Asia	1,898,468 (1161224–2,826,889)	779.4 (478.28–1160.06)	3,756,883 (2287241–5,644,606)	773.62 (470.78–1162.43)	0 (−0.01–0.02)
17 Southern Latin America	406,761 (249398–608,336)	1357.56 (832.58–2029.96)	626,033 (385018–934,908)	1346.29 (827.59–2010.32)	−0.01 (−0.05–0.02)
18 Southern Sub-Saharan Africa	212,365 (130591–316,868)	862.37 (531.87–1285.76)	393,811 (241651–589,625)	811.72 (498.85–1214.53)	−0.16 (−0.17−−0.15)
19 Tropical Latin America	1,042,945 (638459–1,565,086)	1234.45 (757.17–1852.26)	1,999,829 (1232711–2,986,297)	1249.75 (769.96–1866.82)	0.02 (0.01–0.04)
20 Western Europe	3,559,397 (2232481–5,246,740)	1325.21 (830.47–1954.78)	3,998,066 (2471532–5,946,859)	1270.85 (783.65–1892.75)	−0.1 (−0.11–−0.08)
21 Western Sub-Saharan Africa	771,757 (475726–1,149,716)	918.64 (568.21–1367.27)	1,988,999 (1214258–2,985,207)	907.23 (556.72–1359.03)	−0.04 (−0.08–0)

DALYs, disability-adjusted life years; ASDR, age-standardized DALYs rate; EAPC, estimated annual percentage change; SDI, sociodemographic index; CI, confidence interval; UI, uncertainty interval.

### 3.2 Sex and age groups level

From 1990 to 2021, global trends revealed a sex-specific pattern in LBP in the working-age group, with both males and females showing increasing incident, prevalence, and DALYs cases, while ASRs declined. Females consistently had higher rates than males across these metrics (Supplementary Figure S2). In 2021, male workers had 171.4 million LBP cases, and females had 281.4 million cases. The incidence rates were 74.1 million for males and 118.4 million for females. DALYs attributed to LBP were 19.7 million for males and 31.8 million for females (Supplementary Figure S3). The ASPR was 6,528.2 per 100,000 people for males and 10,742.5 per 100,000 people for females. The ASIR was 2,826.69 per 100,000 people for males and 4,528.97 per 100,000 people for females. The ASDR was 751.57 per 100,000 people for males and 1,212.77 per 100,000 people for females (Tables 1–3).

Supplementary Figure S4 shows that from 1990 to 2021, incidence, prevalence, and DALYs for LBP increased across all age groups, with the rate of increase accelerating with age. Conversely, the ASR for these metrics consistently declined. In 2021, ASPR, ASIR, and ASDR peaked in the 60–64 age group, while the 50–54 age group had the highest absolute number of cases for incidence, prevalence, and DALYs (Supplementary Figure S5). Further stratification by age and gender in 2021 revealed higher ASR and case numbers in females compared to males for all indicators, a significant pattern (Supplementary Figure S6).

### 3.3 SDI regional level

From 1990 to 2021, there was a consistent increase in incident cases, prevalent cases, and deaths due to LBP among the working-age group within each SDI quintile, while the ASR for these metrics declined (Figure 2). In 2021, high SDI region had higher ASPR (11,662.1 per 100,000 people; 95% UI: 8,953.29–14,956.28), ASIR (4,874.22 per 100,000 people; 95% UI: 3,547.7–6,454.88), and ASDR (1,326.75 per 100,000 people; 95% UI: 851.47–1,927.6) compared to other SDI regions. Middle SDI regions, however, had the highest absolute numbers: 129.4 million prevalent cases (95% UI: 92.4–174.8), 55.6 million incident cases (95% UI: 38.2–76.4), and 14.7 million DALYs (95% UI: 9.0–22.1) (Supplementary Figure S1 and Tables 1–3). The high-middle SDI region saw the steepest decline in ASIR, ASPR, and ASDR between 1990 and 2021, with EAPCs of −0.37 (95% CI: −0.43 to −0.31), −0.39 (95% CI: −0.46 to −0.33), and −0.38 (95% CI: −0.44 to −0.31), respectively (Tables 1–3).

### 3.4 GBD regional level

**Figure 3:**
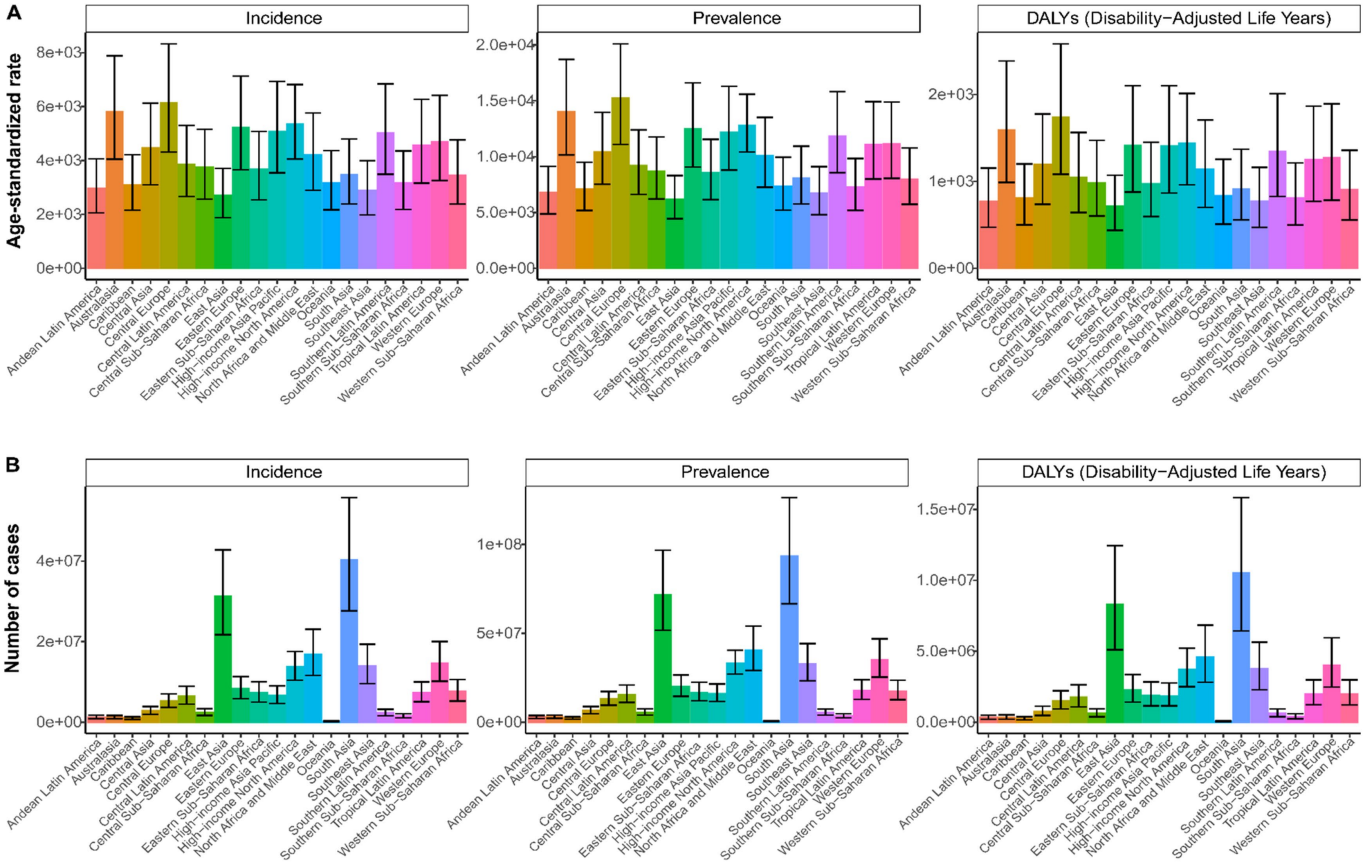
Age-standardized rate **(A)** and number **(B)** of incidence, prevalence, and DALYs of LBP in working-age group by 21 regions, 2021.

**Figure 4:**
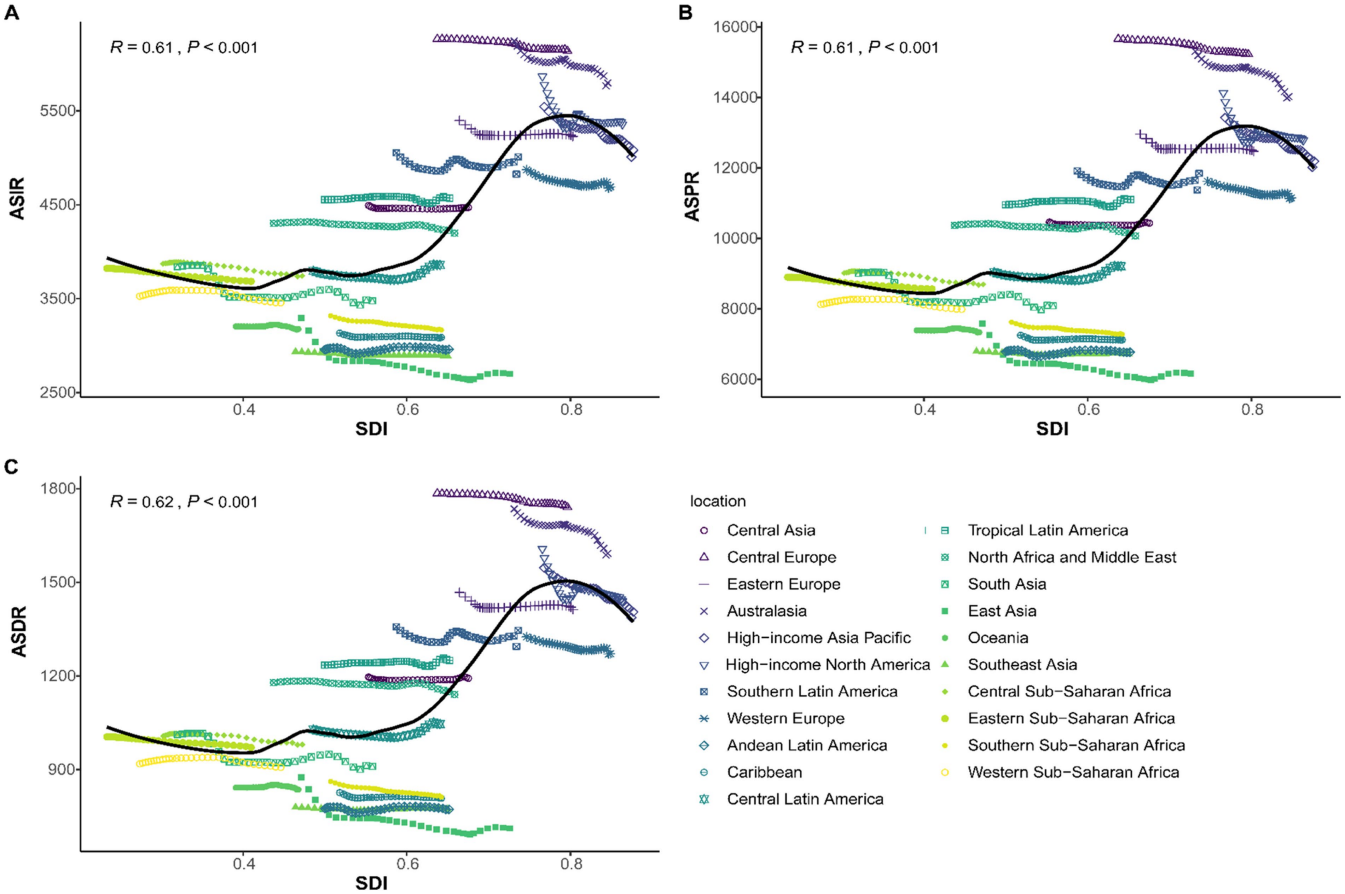
The associations between the socio-demographic index and LBP in working-age group across 21 regions. **(A)** The age-standardized incidence rate; **(B)** The age-standardized prevalence rate; **(C)** The age-standardized DALYs rate.

In 2021, South Asia was identified as the region with the highest prevalence of LBP among the working-age group, with an estimated 93.4 million cases (95% UI: 66.6–126.3), while Oceania had the lowest with 0.56 million cases (95% UI: 0.4–0.76). Among the 21 regions studied, 18 showed an increase in LBP cases, incidence, prevalence, and DALYs, with Central Europe, Eastern Europe, and the High-income Asia Pacific exhibiting decreases. Central Europe had the highest ASIR (6,138.77 per 100,000 people; 95% UI: 4,310.43–8,326.87), ASPR (15,227.76 per 100,000 people; 95% UI: 11,096.56–20,107.97), and ASDR (1,740.57 per 100,000 people; 95% UI: 1,082.21–2,583.67). East Asia recorded the lowest rates for these indicators (Figure 3). Over the past 32 years, Central Latin America saw the most significant increase in LBP incidence, prevalence, and DALYs, with EAPCs of 0.04 (95% CI: 0–0.09), 0.05 (95% CI: 0–0.1), and 0.05 (95% CI: 0–0.09), respectively. East Asia experienced the most substantial decline in these metrics, with EAPCs of −0.46 (95% CI: −0.56−−0.35) for incidence, −0.47 (95% CI: −0.58–−0.35) for prevalence, and −0.46 (95% CI: −0.57–−0.35) for DALYs (Tables 1–3). Figure 4 shows a positive correlation between the ASIR, ASPR, and ASDR for LBP and the SDI. Eight regions, including Central Asia, Australasia, and Eastern Europe, have rates exceeding the global average for three metrics.

### 3.5 National level

**Figure 5:**
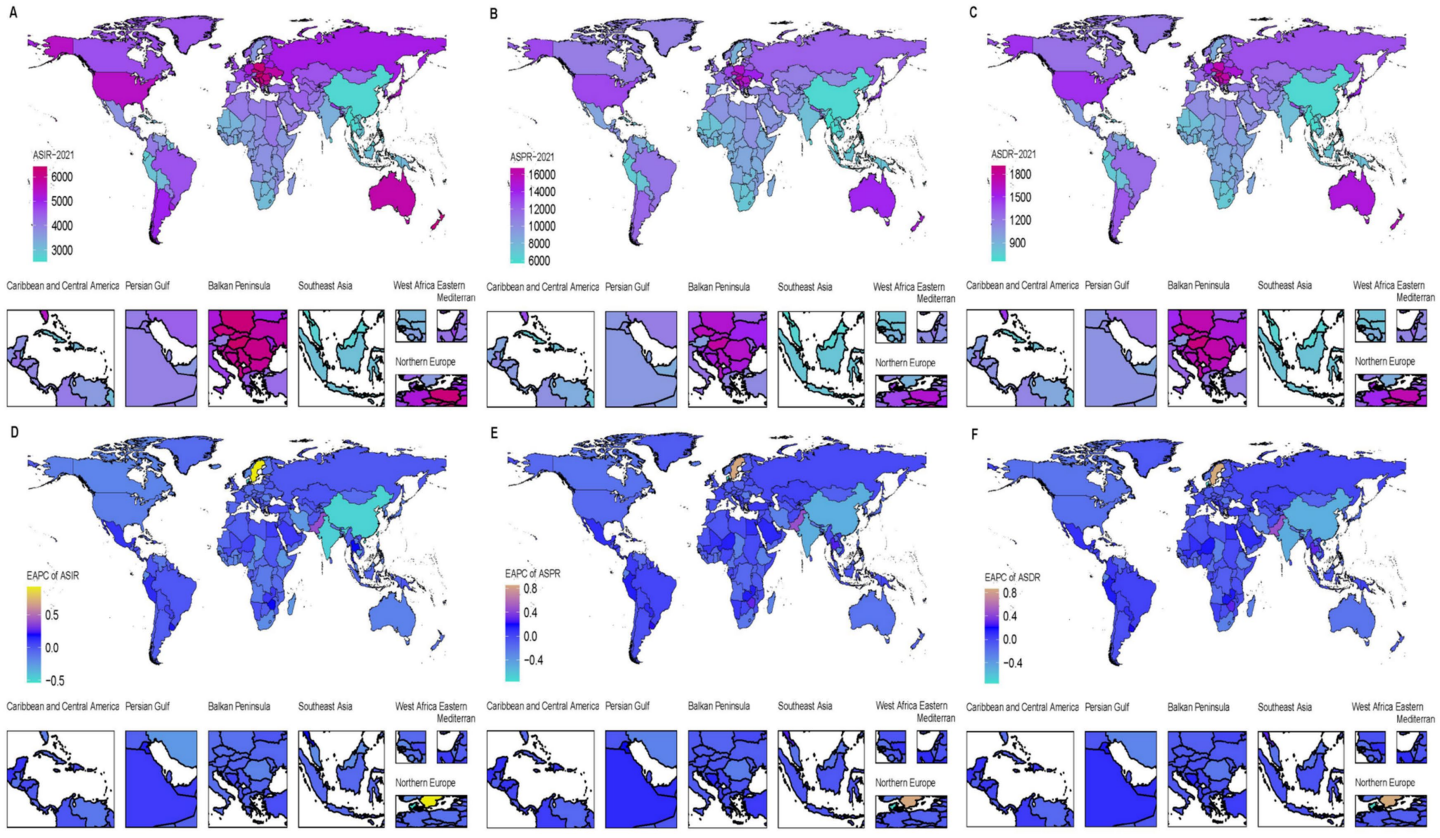
Age-standardized rate of incidence, prevalence, and DALYs of LBP in working-age group in 204 countries and territories in 2021 **(A–C)** and EAPC from 1990 to 2021 **(D–F)**.

Between 1990 and 2021, significant disparities in LBP prevalence were observed across 204 countries. In 2021, Hungary had the highest ASPR at 16,704.84 per 100,000 people (95% UI: 12,202.96–22,041.11), while Maldives had the lowest at 5,704.79 per 100,000 people (95% UI: 4,011.05–7,825.33) (Figure 5). India had the highest number of prevalent cases with 68.5 million (95% UI: 48.8–92.5) (Supplementary Table S3). Sweden showed the most significant increase in prevalence with an EAPC of 0.86 (95% CI: 0.63–1.08), and Denmark had the most notable decline with an EAPC of −0.75 (95% CI: −0.89–−0.62) (Figure 5). China had the highest number of incident cases with 29.8 million (95% UI: 20.6–41.1), followed closely by India with 29.8 million cases (95% UI: 20.5–41.1) (Supplementary Table S2). Hungary also reported the highest ASIR at 6,424.08 per 100,000 people in 2021. Sweden showed the most notable increase in incidence with an EAPC of 0.93 (95% CI: 0.68–1.18) (Figure 4). China led in the number of DALYs cases with 7.9 million (95% UI: 4.8–11.9) (Supplementary Table S4), and Hungary had the highest ASDR at 1,910.1 per 100,000 people. Sweden also exhibited the most notable increase in DALYs with an EAPC of 0.86 (95% CI: 0.64–1.09) (Figure 5).

### 3.6 Joinpoint analysis from 1990 to 2021

**Figure 6:**
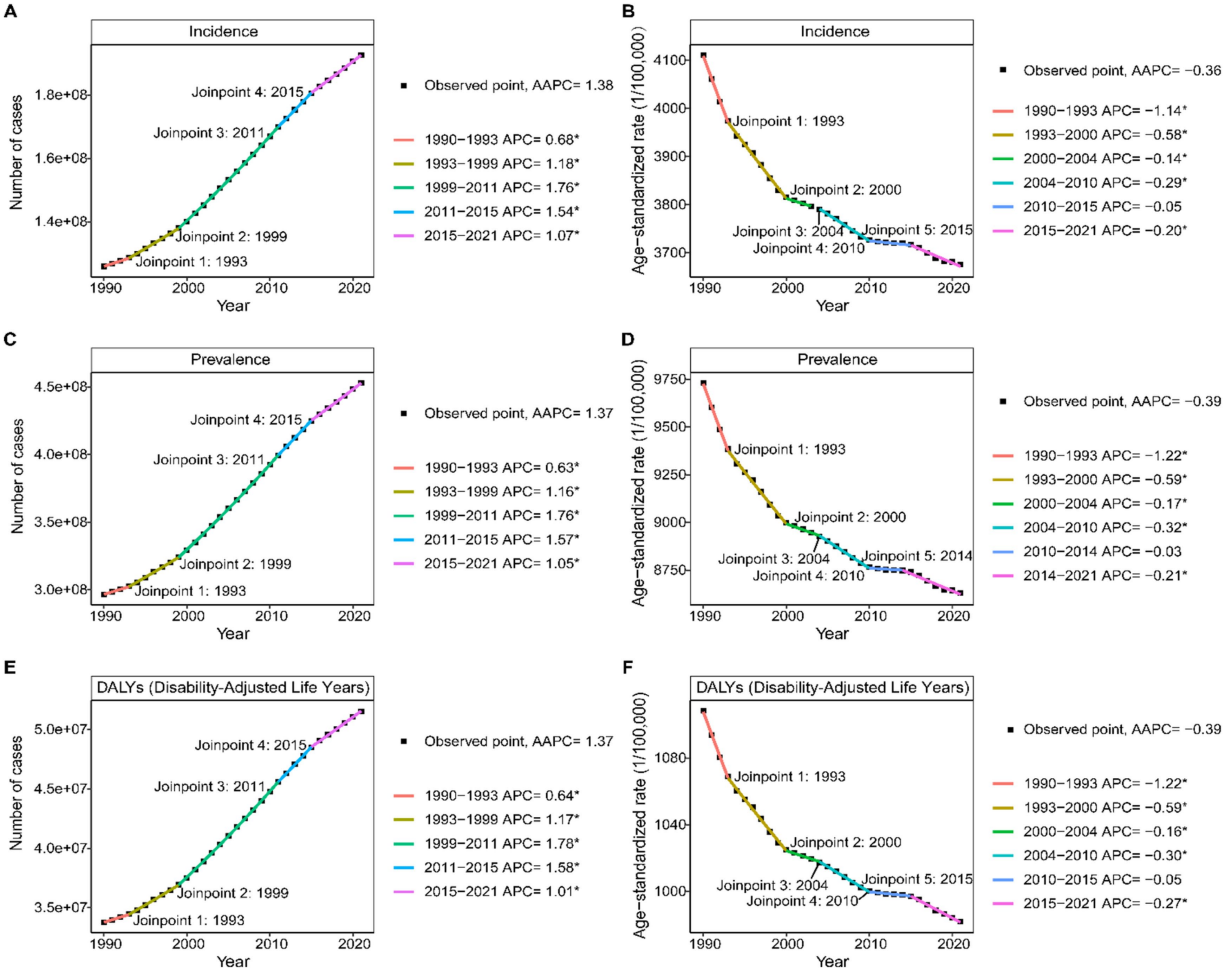
Joinpoint regression analysis of Age-standardized rate **(A,C,D)** and number **(B,D,E)** of incidence, prevalence, and DALYs of LBP in working-age group from 1990 to 2021.

The Joinpoint regression analysis for LBP in the working-age group, as depicted in Figure 6, revealed a significant upward trend in incident cases, prevalence, and DALYs from 1990 to 2021, with four phases: 1990–1993, 1993–1999, 1999–2011, and 2011–2015, followed by 2015–2021. The steepest increase was observed between 1999 and 2011 (Supplementary Table S5). Conversely, the ASR for these metrics showed an overall downward trend, with five transition points: 1990–1993, 1993–2000, 2000–2004, 2004–2010, 2010–2014, and 2014–2021. Except for the stable period from 2010 to 2014, all other intervals showed a decline, with the most significant drop between 1990 and 1993 (Supplementary Table S6).

### 3.7 Decomposition analysis of LBP

**Figure 7:**
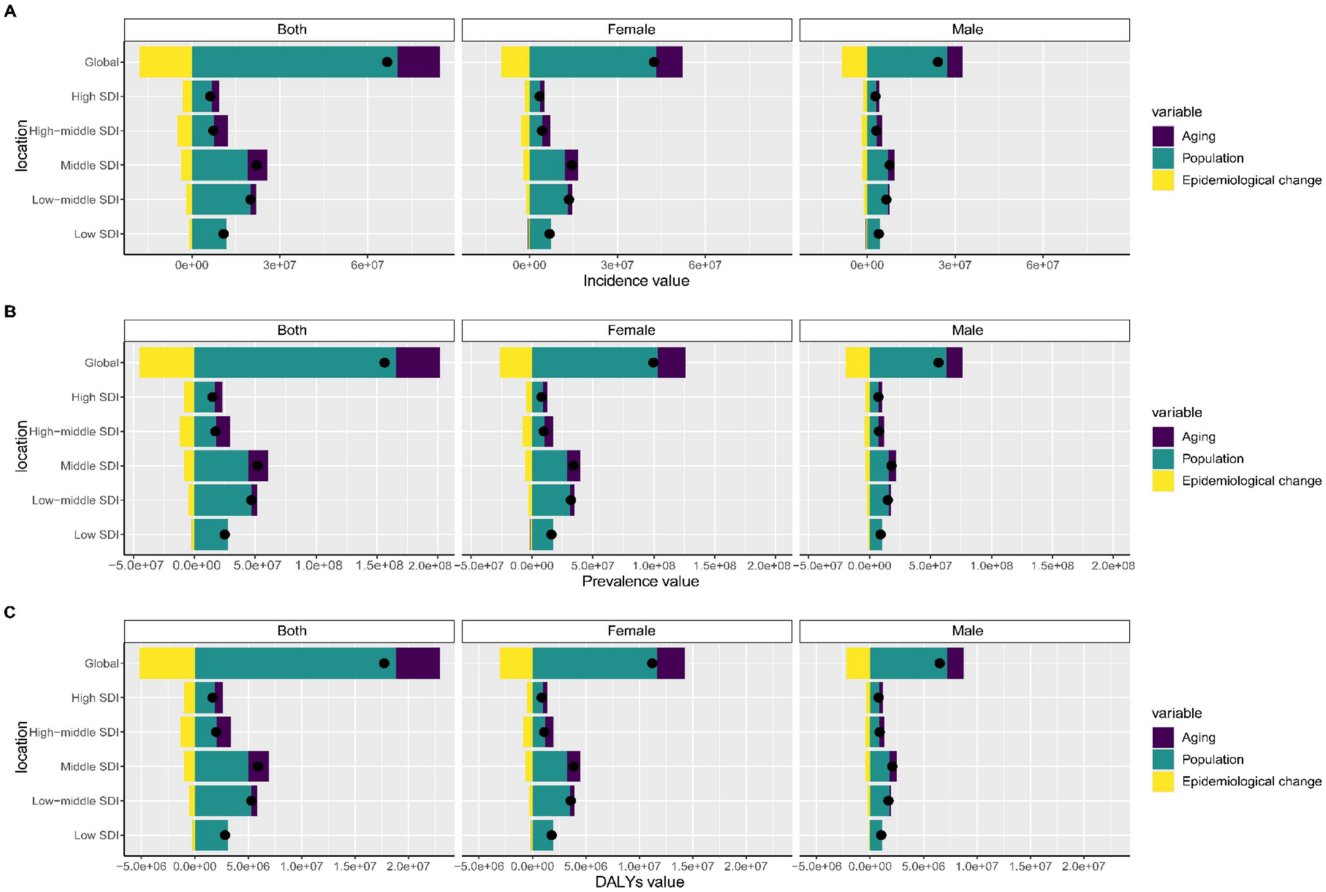
Decomposition analysis of LBP in working-age group change from 1990 to 2021 at global level by SDI quintile and sex. **(A)** Incidence; **(B)** Prevalence; **(C)** DALYs.

Our decomposition analysis indicates that aging, population growth, and epidemiological changes significantly contribute to the trends in LBP incidence, prevalence, and DALYs among the working-age group across five SDI region (Figure 7). Globally, these factors account for 21.43, 105.46%, and −26.9% of the increase in incidence; 22.95, 105.93%, and −28.88% of the increase in prevalence; and 23.09, 106.17%, and −29.27% of the increase in DALYs, respectively. Population growth is identified as the primary driver of the global increase in LBP burden. In the high-middle SDI region, these factors contribute 66.28, 103.43%, and −69.71% to prevalence, respectively. The Low SDI region uniquely shows a negative aging rate of −1.58%. Females across all subgroups and SDI quintiles consistently have a higher LBP burden compared to males, with population growth being the most influential factor on incidence, prevalence, and DALYs (Supplementary Table S7).

### 3.8 Future burden of LBP

**Figure 8:**
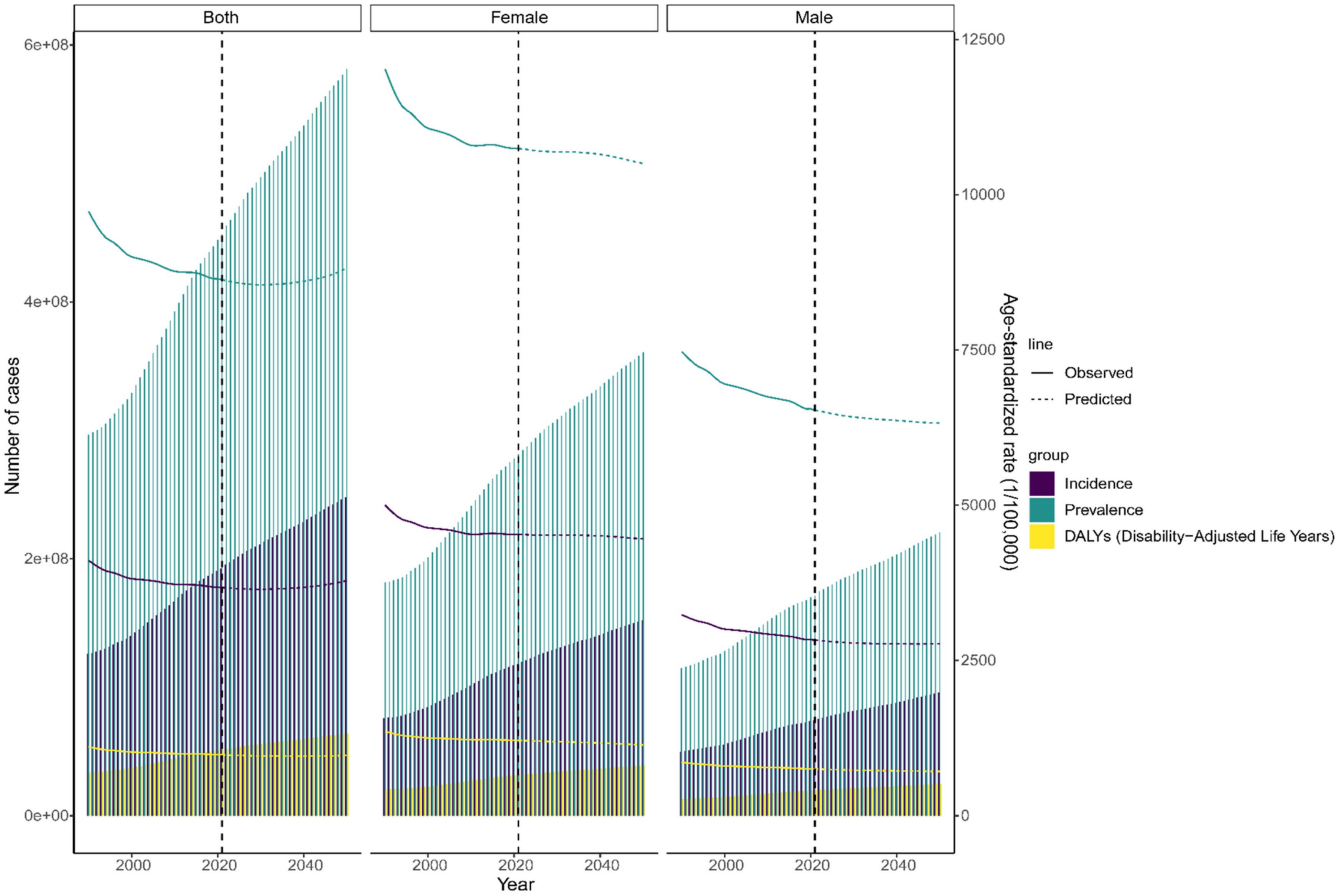
Future forecasts of global burden of LBP in working-age group.

Figure 8 project a significant increase in global incidence, prevalence, and DALYs for LBP among the working-age group by 2050. The incidence is projected to reach 248.1 million cases, prevalence 581.3 million cases, and DALYs 64 million cases. Despite these increases, the ASRs are anticipated to decrease. Females are also projected to consistently have higher case numbers and ASRs compared to males (Supplementary Table S8).

## 4 Discussion

Our study leverages the latest GBD 2021 data to offer the most current epidemiological insights into the burden of LBP among working-age group. We present global, regional, and national data on incidence, prevalence, and DALYs from 1990 to 2021, complemented by a thorough assessment that includes trend analysis, decomposition, and projection modeling. Despite a decline in the ASR of LBP, there has been a significant increase in the number of cases and DALYs. Decomposition analysis indicates that population growth is the predominant driver of the increasing burden. Projections reveal that while the ASR is expected to continue declining through 2050, the absolute number of cases will rise. This underscores the substantial challenges in controlling and managing LBP in the forthcoming decades.

In 2021, the global prevalence of LBP among the working-age group reached an estimated 452.8 million cases. This figure, along with 19.25 million incident cases and 51.51 million DALYs, underscores the profound impact of LBP on workers’ health, productivity, healthcare systems, and economic costs. The global prevalence and incidence rates of LBP highlight its widespread nature, contributing significantly to the disability burden within the workforce (26). The high DALYs reflect the chronic nature of LBP, requiring a multifaceted management approach (27). According to the classification of 21 regions, South Asia had the highest number of cases, likely due to a large working-age group and physically demanding jobs (28). Conversely, Central Europe exhibited the highest ASRs for LBP incidence, prevalence, and DALYs, likely due to a combination of sedentary work patterns, an aging population, and robust health reporting systems (29). The high burden of LBP in Central Latin America is particularly notable, with the region seeing the largest rise in ASR for incidence, prevalence, and DALYs from 1990 to 2021. This may be driven by economic development, urbanization, and shifts in occupational structures that increase exposure to ergonomic risk factors, such as prolonged standing, heavy lifting, and poor workplace ergonomics. Additionally, increased healthcare access may have led to better reporting of LBP cases over time (30). Nationally, India reports the highest number of prevalent LBP cases, a trend likely attributed to its large population and the significant proportion of manual laborers. China, on the other hand, has the highest number of incident cases and DALYs, which could be associated with factors such as industrialization, urbanization, and an aging workforce (10). Hungary notably reported the highest ASR for LBP incidence, prevalence, and DALYs, potentially attributable to the high prevalence of chronic musculoskeletal conditions, an aging population, and a well-developed healthcare system. Conversely, Sweden experienced the most significant increase in LBP burden, likely due to factors such as increased life expectancy, an aging demographic, and enhanced access to healthcare, which may reflect improved diagnostics rather than an actual increase in the number of cases (31). Peru has experienced the most notable increase in LBP incidence, likely a consequence of socioeconomic changes, urbanization, and heightened exposure to occupational risk factors within sectors such as construction and agriculture (27).

A striking finding from our study is the consistently high LBP burden in working-age women compared to men, even after age adjustment. This aligns with prior research emphasizing that women are more susceptible to chronic pain conditions like LBP due to biological factors such as hormonal fluctuations, pregnancy, and menopause, which may affect musculoskeletal stability through changes in estrogen and relaxin levels (31). Women are also more likely to be employed in physically demanding yet ergonomically poor occupations-such as healthcare, retail, and domestic work-that involve repetitive lifting and awkward postures (32, 33). Additionally, lower physical activity levels and caregiving responsibilities may lead to muscle deconditioning and delayed healthcare-seeking behavior, further increasing LBP risk. Although occupational hazards are a major contributor, environmental exposures such as air pollution, extreme temperatures and poor urban planning may also exacerbate LBP by promoting inflammation and limiting physical activity (34, 35). These findings highlight the need for gender-sensitive strategies, including ergonomic workplace improvements, musculoskeletal health education, and accessible rehabilitation services for women.

Notably, when analyzing age-specific trends, the 50–54 age group exhibited the highest ASR for incidence, prevalence, and DALYs, whereas the 60–64 age group had the highest total number of cases. This indicates that while the aging population contributes to the increased burden of LBP, the peak ASRs may indicate a higher proportion of individuals experiencing LBP-related disability within the working-age population. The higher absolute case numbers in the older cohort could be attributed to extended life expectancy and the cumulative impact of long-term physical stressors, such as repetitive physical labor or prolonged periods of sitting (27). Furthermore, addressing these disparities is crucial not only for enhancing health outcomes but also for bolstering labor force participation. As LBP is the leading cause of disability globally, it significantly contributes to lost productivity (11).

Correlation analyses revealed a positive correlation between the ASR for LBP in the working-age group and the SDI, underscoring the significant link between economic development and LBP burden. Despite significant increases in incident cases, prevalent cases, and DALYs across all regions from 1990 to 2021, the ASRs for these metrics generally declined over time, with several notable transition points. This apparent paradox-declining ASRs alongside rising absolute numbers-can be attributed to global population growth and aging, particularly in middle- and high-SDI countries where life expectancy has increased. Moreover, improvements in healthcare access, diagnostic accuracy, and reporting systems may have enhanced case detection while simultaneously promoting earlier intervention and better management of LBP, thereby reducing its severity or chronicity at a population level (36, 37). This trend suggests that while the global burden of LBP is rising in absolute numbers, health interventions, enhanced reporting, and population aging may have collectively reduced the incidence rate per unit of population (38). The period from 1999 to 2011, which saw the most rapid growth in LBP cases, prevalence, and DALYs, likely reflects global transitions in occupational behaviors, including the rise of sedentary office work and increasing exposure to risk factors such as poor posture and prolonged screen time (27, 39). The stabilization of trends from 2010 to 2014 may suggest the effectiveness of health interventions, followed by a slight decline in the subsequent years (2015–2021) (40). Notably, in many regions with high SDI, aging contributes significantly to the burden of LBP; for instance, in medium to high SDI regions, the aging population is becoming increasingly vulnerable to chronic musculoskeletal disorders, including LBP. With advancing age, the risk of developing degenerative spinal conditions escalates, as does the cumulative effect of occupational labor or sedentary behavior (41). Conversely, the negative impact of epidemiological changes observed in the decomposition analysis may reflect improvements in healthcare systems, including earlier diagnosis, better management of LBP through multidisciplinary care, and increased availability of physical therapy and rehabilitation services. These advances may have helped reduce the incidence, severity, or chronic progression of LBP in certain regions, thereby offsetting some of the burden (42, 43).

While this study offers valuable insights into the global burden of LBP among working-age group, several limitations should be considered. Firstly, despite utilizing the latest GBD 2021 data and sophisticated modeling techniques, the data’s accuracy and applicability might be compromised by variations in reporting standards, healthcare system disparities, and inconsistencies in disease definitions, especially in low- and middle-income countries. Secondly, although the study conducted extensive epidemiological trend analyses across different regions, the models may not fully account for region-specific sociocultural influences, workplace ergonomics, or healthcare accessibility, which could play crucial roles in shaping LBP prevalence. Understanding these factors is essential for designing tailored interventions that target high-burden areas more effectively. Thirdly, while ASR were employed for comparative analyses, the models did not incorporate other confounding factors, such as comorbid chronic conditions (e.g., obesity, diabetes) and mental health disorders (e.g., depression, anxiety), which are known to influence the development, severity, and chronicity of LBP. These interactions were not fully explored due to limitations in the GBD dataset. Finally, projections of future trends rely on current data and assumptions, which may not fully account for unforeseen events such as pandemics or significant policy shifts that could substantially alter the projected burden of LBP (44–46). To address these limitations, future research should prioritize more detailed data collection and longitudinal cohort studies to yield more precise and region-specific insights. This approach will enable the development of more targeted interventions and enhance the evidence base for public health policy-making.

## 5 Conclusion

This study underscores the escalating burden of LBP in the working-age group from 1990 to 2021, characterized by significant increases in incident, prevalent and DALYs cases. Despite a decline in ASRs, population growth and aging are identified as the primary drivers of the escalating absolute burden. Women consistently exhibit a higher burden compared to men, with regions such as South Asia and Central Europe reporting the highest absolute case numbers. Projections indicate a continued global increase in LBP burden until 2050, albeit with a decline in ASR. These findings highlight an urgent need for comprehensive public health responses. Policymakers should prioritize integrating LBP prevention into primary healthcare systems, promoting workplace ergonomic improvements, and implementing gender-sensitive public health initiatives. Additionally, improving access to rehabilitation services and strengthening public awareness campaigns can help reduce the long-term impact of LBP on health systems, workforce productivity, and economic stability.

## Data Availability

Publicly available datasets were analyzed in this study. This data can be found: GBD 2021, https://ghdx.healthdata.org/gbd-2021.

## Footnotes

^1^ http://ghdx.healthdata.org/

## References

[1] Global pattern, trend, and cross-country inequality of early musculoskeletal disorders from 1990 to 2019, with projection from 2020 to 2050. Med. (2024) 5:943–62.e6. doi: 10.1016/j.medj.2024.04.009, PMID: 38834074 PMC11321819

[2] Global, regional, and national burden of low back pain, 1990-2020, its attributable risk factors, and projections to 2050: a systematic analysis of the global burden of disease study 2021. Lancet Rheumatol. (2023) 5:e316–29. doi: 10.1016/S2665-9913(23)00098-X, PMID: 37273833 PMC10234592

[3] Global incidence, prevalence, years lived with disability (YLDs), disability-adjusted life-years (DALYs), and healthy life expectancy (HALE) for 371 diseases and injuries in 204 countries and territories and 811 subnational locations, 1990-2021: a systematic analysis for the global burden of disease study 2021. Lancet. (2024) 403:2133–61. doi: 10.1016/S0140-6736(24)00757-8 38642570 PMC11122111

[4] Knezevic NN Candido KD Vlaeyen JWS Van Zundert J Cohen SP. Low back pain. Lancet. (2021) 398:78–92. doi: 10.1016/S0140-6736(21)00733-9, PMID: 34115979

[5] Population and fertility by age and sex for 195 countries and territories, 1950-2017: a systematic analysis for the global burden of disease study 2017. Lancet. (2018) 392:1995–2051. doi: 10.1016/S0140-6736(18)32278-5, PMID: 30496106 PMC6227915

[6] Zhou T Salman D McGregor AH. Recent clinical practice guidelines for the management of low back pain: a global comparison. BMC Musculoskelet Disord. (2024) 25:344. doi: 10.1186/s12891-024-07468-0, PMID: 38693474 PMC11061926

[7] Wu Z Huang G Ai J Liu Y Pei B. The burden of low back pain in adolescents and young adults. J Back Musculoskelet Rehabil. (2024) 37:955–66. doi: 10.3233/BMR-230215, PMID: 38517768 PMC11321494

[8] Chen S Chen M Wu X Lin S Tao C Cao H. Global, regional and national burden of low back pain 1990-2019: a systematic analysis of the global burden of disease study 2019. J Orthop Translat. (2022) 32:49–58. doi: 10.1016/j.jot.2021.07.005, PMID: 34934626 PMC8639804

[9] Daryabor A Akbarzadeh BA. Comparison of a 30-year trend of incidence, prevalence, and DALY due to low back pain in Iran with low- and high-SDI countries; based on GBD study 2019 data. Arch Acad Emerg Med. (2024) 12:e39. doi: 10.22037/aaem.v12i1.2257, PMID: 38737131 PMC11088793

[10] Xu S Qi J Liu C Xia W Wang Z Li K. Evaluation of three decades of the burden of low back pain in China before COVID-19: estimates from the global burden of disease database 2019. J Glob Health. (2024) 14:04006. doi: 10.7189/jogh.14.04006, PMID: 38487857 PMC10940963

[11] Burden of disease scenarios for 204 countries and territories, 2022-2050: a forecasting analysis for the global burden of disease study 2021. Lancet. (2024) 403:2204–56. doi: 10.1016/S0140-6736(24)00685-8 38762325 PMC11121021

[12] Global, regional, and national incidence, prevalence, and years lived with disability for 354 diseases and injuries for 195 countries and territories, 1990-2017: a systematic analysis for the global burden of disease study 2017. Lancet. (2018) 392:1789–858. doi: 10.1016/S0140-6736(18)32279-7, PMID: 30496104 PMC6227754

[13] Kocarnik JM Compton K Dean FE Fu W Gaw BL Harvey JD. Cancer incidence, mortality, years of life lost, years lived with disability, and disability-adjusted life years for 29 Cancer groups from 2010 to 2019: a systematic analysis for the global burden of disease study 2019. JAMA Oncol. (2022) 8:420–44. doi: 10.1001/jamaoncol.2021.6987, PMID: 34967848 PMC8719276

[14] Stevens GA Alkema L Black RE Boerma JT Collins GS Ezzati M. Guidelines for accurate and transparent health estimates reporting: the GATHER statement. Lancet. (2016) 388:e19–23. doi: 10.1016/S0140-6736(16)30388-9, PMID: 27371184

[15] Mathew G Agha R Albrecht J Goel P Mukherjee I Pai P. STROCSS 2021: strengthening the reporting of cohort, cross-sectional and case-control studies in surgery. Int J Surg. (2021) 96:106165. doi: 10.1016/j.ijsu.2021.106165, PMID: 34774726

[16] OECD. Employment rate by age group. (2015). Available online at: https://www.oecd.org/en/data/indicators/employment-rate-by-age-group.html (Accessed January 1, 2015).

[17] Wang S Dong Z Wan X. Global, regional, and national burden of inflammatory bowel disease and its associated anemia, 1990 to 2019 and predictions to 2050: An analysis of the global burden of disease study 2019. Autoimmun Rev. (2024) 23:103498. doi: 10.1016/j.autrev.2023.103498, PMID: 38052263

[18] Sung H Ferlay J Siegel RL Laversanne M Soerjomataram I Jemal A. Global Cancer statistics 2020: GLOBOCAN estimates of incidence and mortality worldwide for 36 cancers in 185 countries. CA Cancer J Clin. (2021) 71:209–49. doi: 10.3322/caac.21660, PMID: 33538338

[19] Cao F Liu YC Ni QY Chen Y Wan CH Liu SY. Temporal trends in the prevalence of autoimmune diseases from 1990 to 2019. Autoimmun Rev. (2023) 22:103359. doi: 10.1016/j.autrev.2023.103359, PMID: 37201621

[20] Cao F Pan HF Hou S. A novel metric of autoimmune disease burden and its estimated incidence across different stages in life cycle of women. Autoimmun Rev. (2024) 23:103671. doi: 10.1016/j.autrev.2024.103671, PMID: 39442592

[21] Bai Z Han J An J Wang H Du X Yang Z. The global, regional, and national patterns of change in the burden of congenital birth defects, 1990-2021: an analysis of the global burden of disease study 2021 and forecast to 2040. EClinicalMedicine. (2024) 77:102873. doi: 10.1016/j.eclinm.2024.102873, PMID: 39416384 PMC11474384

[22] Liang X Lyu Y Li J Li Y Chi C. Global, regional, and national burden of preterm birth, 1990-2021: a systematic analysis from the global burden of disease study 2021. EClinicalMedicine. (2024) 76:102840. doi: 10.1016/j.eclinm.2024.102840, PMID: 39386159 PMC11462015

[23] Cao F Xu Z Li XX Fu ZY Han RY Zhang JL. Trends and cross-country inequalities in the global burden of osteoarthritis, 1990-2019: a population-based study. Ageing Res Rev. (2024) 99:102382. doi: 10.1016/j.arr.2024.102382, PMID: 38917934

[24] Huang D Lai H Shi X Jiang J Zhu Z Peng J. Global temporal trends and projections of acute hepatitis E incidence among women of childbearing age: age-period-cohort analysis 2021. J Infect. (2024) 89:106250. doi: 10.1016/j.jinf.2024.106250, PMID: 39181413

[25] Hu W Fang L Zhang H Ni R Pan G. Global disease burden of COPD from 1990 to 2019 and prediction of future disease burden trend in China. Public Health. (2022) 208:89–97. doi: 10.1016/j.puhe.2022.04.015, PMID: 35728417

[26] Chen N Fong DYT Wong JYH. The global health and economic impact of low-back pain attributable to occupational ergonomic factors in the working-age population by age, sex, geography in 2019. Scand J Work Environ Health. (2023) 49:487–95. doi: 10.5271/sjweh.4116, PMID: 37634250 PMC10838400

[27] Yang Y Lai X Li C Yang Y Gu S Hou W. Focus on the impact of social factors and lifestyle on the disease burden of low back pain: findings from the global burden of disease study 2019. BMC Musculoskelet Disord. (2023) 24:679. doi: 10.1186/s12891-023-06772-5, PMID: 37633880 PMC10464198

[28] Wu A March L Zheng X Huang J Wang X Zhao J. Global low back pain prevalence and years lived with disability from 1990 to 2017: estimates from the global burden of disease study 2017. Ann Transl Med. (2020) 8:299. doi: 10.21037/atm.2020.02.175, PMID: 32355743 PMC7186678

[29] Wang L Ye H Li Z Lu C Ye J Liao M. Epidemiological trends of low back pain at the global, regional, and national levels. Eur Spine J. (2022) 31:953–62. doi: 10.1007/s00586-022-07133-x, PMID: 35217914

[30] Zhang C Qin L Yin F Chen Q Zhang S. Global, regional, and national burden and trends of low back pain in middle-aged adults: analysis of GBD 1990–2021 with projections to 2050. BMC Musculoskelet Disord. (2024) 25:886. doi: 10.1186/s12891-024-08002-y, PMID: 39511565 PMC11542344

[31] Bizzoca D Solarino G Pulcrano A Brunetti G Moretti AM Moretti L. Gender-related issues in the Management of low-Back Pain: a current concepts review. Clin Pract. (2023) 13:1360–8. doi: 10.3390/clinpract13060122, PMID: 37987423 PMC10660510

[32] Dev R Raparelli V Bacon SL Lavoie KL Pilote L Norris CM. Impact of biological sex and gender-related factors on public engagement in protective health behaviours during the COVID-19 pandemic: cross-sectional analyses from a global survey. BMJ Open. (2022) 12:e059673. doi: 10.1136/bmjopen-2021-059673, PMID: 35688591 PMC9189548

[33] Nielsen MW Stefanick ML Peragine D Neilands TB Ioannidis JPA Pilote L. Gender-related variables for health research. Biol Sex Differ. (2021) 12:23. doi: 10.1186/s13293-021-00366-3, PMID: 33618769 PMC7898259

[34] Chen J Liao Y Luo M Tang S Huang J Chen R. Environmental polycyclic aromatic hydrocarbon exposure is associated with low back pain. Environ Geochem Health. (2023) 45:5093–107. doi: 10.1007/s10653-023-01567-y 37069329

[35] Cheng B Pan C Cai Q Liu L Cheng S Yang X. Long-term ambient air pollution and the risk of musculoskeletal diseases: a prospective cohort study. J Hazard Mater. (2024) 466:133658. doi: 10.1016/j.jhazmat.2024.133658, PMID: 38310839

[36] Safiri S Kolahi AA Cross M Hill C Smith E Carson-Chahhoud K. Prevalence, deaths, and disability-adjusted life years due to musculoskeletal disorders for 195 countries and territories 1990-2017. Arthritis Rheumatol. (2021) 73:702–14. doi: 10.1002/art.41571, PMID: 33150702

[37] Cheng M Xue Y Cui M Zeng X Yang C Ding F. Global, regional, and National Burden of low Back pain: findings from the global burden of disease study 2021 and projections to 2050. Spine. (2025) 50:E128–39. doi: 10.1097/BRS.0000000000005265, PMID: 39838749 PMC11888834

[38] Zheng DKY Kawchuk GN Bussières AE Al Zoubi FM Hartvigsen J Fu SN. Trends of low Back pain research in older and working-age adults from 1993 to 2023: a bibliometric analysis. J Pain Res. (2023) 16:3325–41. doi: 10.2147/JPR.S425672, PMID: 37808461 PMC10557964

[39] Ostern N Perscheid G Reelitz C Moormann J. Keeping pace with the healthcare transformation: a literature review and research agenda for a new decade of health information systems research. Electron Mark. (2021) 31:901–21. doi: 10.1007/s12525-021-00484-1, PMID: 35599689 PMC8285287

[40] Shu J Jin W. Prioritizing non-communicable diseases in the post-pandemic era based on a comprehensive analysis of the GBD 2019 from 1990 to 2019. Sci Rep. (2023) 13:13325. doi: 10.1038/s41598-023-40595-7, PMID: 37587173 PMC10432467

[41] Maurer E Klinger C Lorbeer R Rathmann W Peters A Schlett CL. Long-term effect of physical inactivity on thoracic and lumbar disc degeneration-an MRI-based analysis of 385 individuals from the general population. Spine J. (2020) 20:1386–96. doi: 10.1016/j.spinee.2020.04.016, PMID: 32360761

[42] Delitto A George SZ Van Dillen L Whitman JM Sowa G Shekelle P. Low Back pain. J Orthop Sports Phys Ther. (2012) 42:A1–A57. doi: 10.2519/jospt.2012.42.4.A1, PMID: 22466247 PMC4893951

[43] Kamper SJ Apeldoorn AT Chiarotto A Smeets RJ Ostelo RW Guzman J. Multidisciplinary biopsychosocial rehabilitation for chronic low Back pain: Cochrane systematic review and Meta-analysis. BMJ. (2015) 350:h444. doi: 10.1136/bmj.h444, PMID: 25694111 PMC4353283

[44] Petrucci G Papalia GF Russo F Vadalà G Piredda M De Marinis MG. Psychological approaches for the integrative Care of Chronic low Back Pain: a systematic review and meta-analysis. Int J Environ Res Public Health. (2021) 19:60. doi: 10.3390/ijerph19010060, PMID: 35010319 PMC8751135

[45] Wyper GMA. The global burden of disease study and population health metrics. Popul Health Metrics. (2024) 22:35. doi: 10.1186/s12963-024-00357-7, PMID: 39673070 PMC11639115

[46] Nong J Su C Li C Wang C Li W Li Y. Global, regional, and national epidemiology of childhood neuroblastoma (1990-2021): a statistical analysis of incidence, mortality, and DALYs. EClinicalMedicine. (2025) 79:102964. doi: 10.1016/j.eclinm.2024.102964, PMID: 39720601 PMC11667623

