# Supplementary material for: Global, regional, and national burden of low back pain in working-age population from 1990 to 2021 and projections for 2050
Source: Front Public Health. 2025 Apr 24;13:1559355. doi: 10.3389/fpubh.2025.1559355 (PMC12058504; doi:10.3389/fpubh.2025.1559355)
Supplement: Supplementary file 1 [file Data_Sheet_1.PDF]

Online Supplementary Content

Table S1. List of International Classificatin of Diseases (ICD) codes in the GBD 2021.

Table S2. The case number and ASR of incidence of LBP in working-age group between 1990 and 2021 by countries/territories, with EAPC from 1990 to 2021.

Table S3. The case number and ASR of prevalence of LBP in working-age group between 1990 and 2021 by countries/territories, with EAPC from 1990 to 2021.

Table S4. The case number and ASR of DALYs of LBP in working-age group between 1990 and 2021 by countries/territories, with EAPC from 1990 to 2021.

Table S5. The APC and AAPC in case number of LBP in working-age group of incidence, prevalence and DALYs from 1990 to 2021.

Table S6. The APC and AAPC in ASR of LBP in working-age group of incidence, prevalence and DALYs from 1990 to 2021.

Table S7. Decomposition analysis of LBP in working-age group change in incidence, prevalence, and DALYs by SDI quintile and sex, 1990 to 2021.

Table S8. The predicted case number and ASR of incidence, prevalence, and DALYs of LBP in working-age group from 2022 to 2050 globally.

Figure S1. Age-standardized rate and number of incidence, prevalence, and DALYs of LBP in working-age group by Global and SDI, 2021.

Figure S2. Age-standardized rate and number of incidence, prevalence, and DALYs change curves for LBP in working-age group by sex from 1990 to 2021.

Figure S3. Age-standardized rate and number of incidence, prevalence, and DALYs of LBP in working-age group by sex, 2021.

Figure S4. Age-standardized rate and number of incidence, prevalence, and DALYs change curves for LBP in working-age group by sex from 1990 to 2021.

Figure S5. Age-standardized rate and number of incidence, prevalence, and DALYs of LBP in working-age group by age, 2021.

Figure S6. Age- standardized rate and number of incidence, prevalence, and DALYs of LBP in working-age group by sex, 2021.

Table S1. List of International Classificatin of Diseases (ICD) codes in the GBD 2021.

Cause name	ICD10	ICD9
Low back pain	G54.4, M47.015-M47.019, M47.15-M47.18, M47.25-M47.28, M47.815-M47.818, M47.896-M47.899, M48.05-M48.08, M48.16-M48.19, M48.25-M48.27, M48.35-M48.38, M48.45-M48.48, M48.55-M48.58, M49.85-M49.88, M51.05-M51.07, M51.15-M51.17, M51.25-M51.27, M51.35-M51.37, M51.45-M51.47, M51.85-M51.87, M53.3, M53.85-M53.88, M54.05-M54.09, M54.15-M54.18, M54.3-M54.5, M99.03-M99.04, M99.13-M99.14, M99.23-M99.24, M99.33-M99.34, M99.43-M99.44, M99.53-M99.54, M99.63-M99.64, M99.73-M99.74, M99.83-M99.84	353.1, 353.4, 721.3, 721.42, 722.10, 722.32, 722.52, 722.73, 722.83, 722.93, 724.02-724.03, 724.2-724.3, 724.6-724.79

Table S2. The case number and ASR of incidence of LBP in working-age group between 1990 and 2021 by countries/territories, with EAPC from 1990 to 2021.

Location	Cases in 1990 (95% UI)	ASIR (1/100,000) (95% UI)	Cases in 2021 (95% UI)	ASIR (1/100,000) (95% UI)	EAPC (95% CI)
Afghanistan	201453 (138084-276239)	4160.35 (2850.17-5725.34)	598030 (409944-818186)	4157.79 (2850.19-5699.54)	-0.01 (-0.02-0)
Albania	114352 (79387-156149)	6123.27 (4264.37-8344.99)	119581 (83240-162821)	6138.5 (4276.95-8377.74)	0.01 (0-0.01)
Algeria	502002 (344462-691795)	4104.8 (2820.59-5656.2)	1153183 (794832-1595719)	4079.8 (2809.44-5639.91)	-0.03 (-0.05--0.02)
American Samoa	797 (544-1102)	3264.55 (2232.41-4503.05)	1067 (734-1481)	3217.42 (2209.82-4472.56)	-0.02 (-0.04--0.01)
Andorra	1867 (1283-2573)	4729.93 (3253.69-6505.7)	3233 (2230-4428)	4576.49 (3151.26-6276.4)	-0.07 (-0.09--0.06)
Angola	177238 (121539-245674)	3886.17 (2670.31-5378.39)	536210 (366739-746300)	3748.13 (2565.73-5205.82)	-0.14 (-0.17--0.1)
Antigua and Barbuda	1054 (720-1464)	3088.28 (2113.77-4281.12)	2067 (1422-2838)	3079.67 (2117.19-4229.71)	-0.01 (-0.03-0)
Argentina	1010713 (700651-1382842)	5054.84 (3503.33-6919.34)	1525777 (1061028-2090533)	4988.84 (3467.85-6836.49)	-0.02 (-0.05-0.02)
Armenia	101443 (69593-138874)	4738.89 (3262.7-6490.17)	105165 (72984-143706)	4749.43 (3294.92-6507.12)	0.04 (0.03-0.05)
Australia	697797 (493822-940848)	6194.92 (4383.26-8348.53)	1017122 (706140-1385526)	5732.45 (3982.74-7817.07)	-0.16 (-0.18--0.13)
Austria	239713 (166978-326424)	4403.68 (3069.02-6000.94)	272332 (188708-372249)	4068.69 (2822.93-5574.7)	-0.06 (-0.13-0)
Azerbaijan	186168 (128423-255875)	4448.87 (3082.22-6105.93)	355859 (245787-485873)	4498.68 (3107.2-6149.71)	0.08 (0.06-0.09)
Bahamas	4572 (3126-6342)	3089.59 (2116.16-4276.83)	8749 (5998-12095)	3082.19 (2112.03-4262.29)	-0.02 (-0.02--0.01)
Bahrain	12153 (8309-16842)	3956.2 (2714.72-5458.94)	46009 (31697-63382)	3886.28 (2673.46-5343.62)	-0.07 (-0.08--0.06)
Bangladesh	2249357 (1560569-3061204)	4630.7 (3220.07-6279.24)	4538741 (3130869-6252134)	4393.5 (3033.9-6044)	-0.12 (-0.16--0.07)
Barbados	4824 (3291-6657)	3133.83 (2141.74-4321.97)	6971 (4740-9631)	3104.66 (2111.2-4301.82)	-0.02 (-0.03--0.02)
Belarus	376788 (261372-514113)	5102.6 (3543.59-6975.53)	370122 (256877-503585)	5075.7 (3512.35-6929.66)	0 (-0.01-0.01)
Belgium	339486 (234108-465251)	4830.26 (3333.4-6619.98)	380976 (264393-520853)	4700.84 (3257.86-6434.59)	-0.06 (-0.07--0.04)
Belize	2652 (1808-3681)	3133.6 (2140.46-4338.45)	8454 (5767-11713)	3158.68 (2156.7-4375)	0.04 (0.03-0.05)
Benin	69474 (47275-96060)	3643.46 (2483.96-5027.69)	203995 (139604-283574)	3465.53 (2377.49-4804.95)	-0.16 (-0.2--0.13)
Bermuda	1347 (923-1864)	3168.02 (2173.08-4378.27)	1535 (1051-2120)	3136.82 (2150.16-4347.37)	-0.03 (-0.04--0.03)
Bhutan	12385 (8558-17045)	4281.55 (2971.47-5884.13)	20623 (14183-28469)	4213.27 (2902.18-5801.94)	-0.04 (-0.06--0.03)
Bolivia (Plurinational State of)	95949 (65729-133465)	3094.06 (2117.66-4297.66)	226698 (155190-313848)	3120.97 (2136.73-4319.1)	0.01 (-0.02-0.05)
Bosnia and Herzegovina	180917 (127001-247147)	5777.48 (4059.92-7897.38)	147849 (103332-201785)	5903.12 (4121.81-8069.07)	0.06 (0.05-0.08)

Botswana	17989 (12291-24891)	3200.2 (2195.73-4413.38)	46221 (31584-64192)	3155.57 (2156.03-4375.6)	-0.03 (-0.04--0.01)
Brazil	3797006 (2610360-5215510)	4571.35 (3147.38-6275.44)	7119114 (4930113-9767292)	4588.73 (3176.03-6294.9)	0 (-0.01-0.01)
Brunei Darussalam	6895 (4759-9489)	4686.27 (3236.41-6425.55)	14917 (10320-20480)	4474.71 (3094.02-6136.89)	-0.11 (-0.13--0.09)
Bulgaria	380055 (265223-516014)	6041.13 (4206.43-8233.64)	295994 (206589-401755)	5933.25 (4137.47-8071.03)	-0.05 (-0.06--0.05)
Burkina Faso	138923 (96546-189196)	3584.76 (2497.38-4880.02)	344565 (234707-478031)	3483.81 (2377.78-4821.82)	-0.15 (-0.18--0.11)
Burundi	89445 (61376-124446)	3852.47 (2652.7-5340.52)	217220 (148278-299271)	3647.68 (2492.87-5018.89)	-0.21 (-0.22--0.2)
Cabo Verde	5161 (3524-7153)	3476.44 (2382.03-4806.53)	11908 (8139-16557)	3231.83 (2209.82-4485.42)	-0.26 (-0.29--0.22)
Cambodia	149413 (102327-207674)	3246.94 (2229.13-4502.82)	315927 (214912-437651)	2994.79 (2039-4145.33)	-0.27 (-0.29--0.25)
Cameroon	168683 (115378-233014)	3732.8 (2562.63-5148.18)	526378 (358847-728579)	3552 (2424.19-4906.31)	-0.19 (-0.22--0.15)
Canada	947630 (654641-1297830)	5045.03 (3484.94-6907.56)	1224359 (847638-1681815)	4632.3 (3206.51-6372.25)	-0.17 (-0.21--0.13)
Central African Republic	47645 (32630-65824)	3838.1 (2634.27-5299.59)	101134 (69384-140075)	3771.31 (2590.66-5214.57)	-0.07 (-0.09--0.06)
Chad	99713 (69385-136122)	4057.18 (2825.17-5531.55)	254770 (174726-353660)	3733.38 (2570.58-5171.47)	-0.23 (-0.31--0.16)
Chile	411821 (286461-561678)	5113.65 (3560.85-6965.98)	678448 (469008-927920)	5046.22 (3489.3-6907.84)	-0.03 (-0.07-0)
China	23603463 (16026169-32772947)	3285.29 (2234.9-4552.36)	29836929 (20555460-41079785)	2666.65 (1834.65-3681.46)	-0.48 (-0.59--0.37)
Colombia	735096 (506267-1012266)	4099.22 (2825.03-5639.58)	1384931 (962331-1883214)	4083.79 (2839.52-5554.79)	0.01 (0-0.02)
Comoros	7003 (4882-9542)	3504.62 (2447.62-4771.37)	15149 (10419-20968)	3496.62 (2406.81-4832.83)	-0.05 (-0.08--0.03)
Congo	38952 (26897-53613)	3633.82 (2511.41-4997.43)	107989 (74240-149312)	3598.95 (2474.58-4962.99)	-0.05 (-0.07--0.04)
Cook Islands	337 (231-470)	3225.02 (2210.27-4495.72)	419 (287-577)	3279.72 (2242.48-4531.33)	0.09 (0.07-0.1)
Costa Rica	64731 (44234-89001)	3959.89 (2711.52-5440.98)	128420 (88059-177459)	3859.39 (2647.16-5332.89)	-0.06 (-0.07--0.06)
Coted'Ivoire	192020 (133102-262950)	3684.49 (2565.73-5039.28)	477922 (325348-671537)	3522.76 (2399.75-4930.9)	-0.14 (-0.18--0.09)
Croatia	213224 (151335-287939)	5940.21 (4214-8031.8)	179029 (124483-242836)	5805.41 (4030.15-7899.18)	-0.05 (-0.08--0.03)
Cuba	221104 (150472-306970)	3149.12 (2143.72-4369.83)	259099 (186008-345466)	2958.61 (2119.32-3956.77)	-0.09 (-0.13--0.06)
Cyprus	23864 (16482-32634)	4781.59 (3302.88-6534.54)	47469 (32829-65355)	4701.63 (3250.12-6468.43)	-0.05 (-0.06--0.04)
Czechia	454151 (319908-614403)	6361.43 (4471.83-8613.49)	473755 (330006-648013)	6205.19 (4307.9-8495.49)	-0.08 (-0.09--0.08)
Democratic People's Republic of Korea	467907 (322171-645625)	3542.14 (2442.21-4886.77)	681326 (467275-943028)	3315 (2273.76-4595.26)	-0.23 (-0.25--0.22)
Democratic Republic of the Congo	644383 (441694-892496)	3893.88 (2676.72-5382.2)	1606525 (1098736-2213584)	3755.4 (2577.11-5157.87)	-0.13 (-0.16--0.11)

Denmark	183270 (125258-252015)	5111.5 (3490.74-7028.62)	184399 (116392-269446)	4531.67 (2856.37-6639.08)	-0.53 (-0.61--0.44)
Djibouti	6631 (4532-9175)	3495.22 (2398.4-4816.34)	24767 (16830-34349)	3328.83 (2261.26-4601.74)	-0.18 (-0.19--0.17)
Dominica	1224 (837-1687)	3194.15 (2183.48-4402.22)	1492 (1021-2071)	3099.79 (2122.98-4308.31)	-0.12 (-0.13--0.11)
Dominican Republic	114366 (79006-156731)	3096.62 (2140.42-4246.63)	223947 (152871-310696)	3156.77 (2155.67-4377.18)	0.04 (0.01-0.06)
Ecuador	151171 (104936-206477)	2983.88 (2071.2-4073.26)	316955 (223882-429497)	2805.73 (1982.22-3801.17)	-0.21 (-0.3--0.11)
Egypt	1196584 (819649-1646662)	4098.35 (2806.56-5636.99)	2617147 (1791141-3610933)	4193.89 (2868.76-5787.35)	0.08 (0.05-0.11)
El Salvador	102719 (70635-140896)	3898.85 (2681.24-5340.82)	156697 (107531-215600)	3949.83 (2713.61-5433.15)	0.08 (0.06-0.09)
Equatorial Guinea	7207 (4922-9899)	3850.47 (2631.81-5282.02)	26221 (17939-36268)	3651.05 (2502.67-5032.26)	-0.2 (-0.22--0.18)
Eritrea	49173 (33498-68398)	3341.1 (2280.45-4637.34)	112720 (76985-155890)	3341 (2281.84-4609.64)	0.03 (0.02-0.04)
Estonia	56389 (39926-76227)	5053.87 (3577.42-6840.36)	47425 (32826-65063)	5013.93 (3470.21-6893.65)	0 (-0.01-0.01)
Eswatini	9401 (6495-12956)	2933.89 (2030.77-4027.12)	17231 (11775-23908)	2893.67 (1983.94-3991.32)	-0.09 (-0.12--0.05)
Ethiopia	824505 (567557-1135143)	3912.08 (2699.65-5370.38)	1836512 (1255171-2540620)	3631.55 (2490.42-5005.28)	-0.24 (-0.26--0.23)
Fiji	13032 (8902-18111)	3243.42 (2217.61-4499.23)	18750 (12845-26036)	3122.51 (2138.08-4337.12)	-0.11 (-0.11--0.11)
Finland	158625 (111698-215357)	4475.22 (3157.87-6062.53)	159890 (110518-219314)	4241.91 (2937.33-5822.73)	-0.1 (-0.12--0.08)
France	1847515 (1284740-2519266)	4719.42 (3283.17-6437.98)	2118331 (1468704-2898971)	4694.57 (3247.96-6440.21)	-0.01 (-0.04-0.01)
Gabon	16806 (11552-23222)	3603.31 (2481.94-4973.96)	36087 (24905-49924)	3583.92 (2477.61-4949.69)	-0.02 (-0.02--0.01)
Gambia	13735 (9401-18984)	3378.64 (2318.62-4654.21)	36173 (24652-50333)	3281.97 (2242.46-4553.12)	-0.1 (-0.14--0.06)
Georgia	161823 (111921-221521)	4252.96 (2947.44-5831.82)	110511 (76283-151525)	4221.08 (2910.34-5803.99)	-0.09 (-0.13--0.05)
Germany	3203561 (2238827-4346832)	5479.61 (3832.69-7432.11)	3248641 (2258458-4407433)	5231.96 (3638.97-7113.63)	-0.08 (-0.1--0.06)
Ghana	221565 (153957-303069)	3331.46 (2319.09-4552.67)	563771 (397575-765001)	3189.25 (2253.36-4312.81)	-0.17 (-0.2--0.15)
Greece	337131 (233973-458919)	4584.07 (3183.77-6251.18)	331918 (228808-452837)	4506.84 (3104.06-6162.75)	-0.06 (-0.09--0.04)
Greenland	1833 (1260-2523)	4810.07 (3315.26-6604.88)	1917 (1322-2633)	4575.13 (3159.65-6287.8)	-0.07 (-0.11--0.03)
Grenada	1339 (912-1849)	3131.78 (2137.06-4320.98)	2271 (1549-3131)	3082.99 (2105.12-4252.71)	-0.04 (-0.05--0.04)
Guam	2587 (1772-3609)	3178.64 (2181.24-4427.72)	3505 (2396-4861)	3154.19 (2158.41-4379.77)	-0.02 (-0.03-0)
Guatemala	164090 (114338-222603)	4422.77 (3086.43-6008)	392552 (270396-538827)	4227.84 (2911.68-5802.66)	-0.1 (-0.13--0.06)
Guinea	96495 (66256-133138)	3609.16 (2480.61-4971.48)	209040 (142712-291157)	3551.22 (2429.11-4932.67)	-0.09 (-0.13--0.05)

Guinea-Bissau	14459 (9917-20144)	3490.68 (2399.78-4848.28)	31699 (21561-43956)	3394.48 (2308.9-4692.85)	-0.1 (-0.13--0.07)
Guyana	12546 (8599-17406)	3122.83 (2145.07-4330.04)	15237 (10420-21192)	3095.51 (2119.03-4304.28)	-0.03 (-0.03--0.02)
Haiti	98001 (67099-135504)	3128.72 (2142.96-4322.81)	232599 (158337-323298)	3112.58 (2119.86-4318.75)	-0.04 (-0.06--0.02)
Honduras	82541 (56526-114272)	3916.68 (2685.41-5417.77)	232515 (159590-319749)	3946 (2709.52-5426.38)	0.04 (0.03-0.04)
Hungary	479821 (336875-653838)	6516.68 (4567.47-8896.51)	449701 (315098-609016)	6424.08 (4490.96-8718.94)	-0.04 (-0.05--0.04)
Iceland	8079 (5572-11060)	5017.99 (3464.54-6864.61)	11545 (8009-15759)	4735.49 (3283.86-6471.19)	-0.19 (-0.2--0.17)
India	16943862 (11647210-23353031)	3756.76 (2587.2-5168.47)	29828629 (20522412-41141295)	3277.53 (2256.57-4515.2)	-0.46 (-0.57--0.35)
Indonesia	3095198 (2116078-4282863)	3127.14 (2141.25-4318.73)	5983552 (4100195-8265177)	3031.56 (2076.43-4187.27)	-0.06 (-0.08--0.05)
Iran (Islamic Republic of)	1327012 (917515-1820351)	4885.48 (3383.66-6702.94)	2694286 (1859639-3705654)	4441.96 (3062.71-6098.67)	-0.25 (-0.3--0.2)
Iraq	351694 (240483-483230)	4113.51 (2815.42-5648.08)	1006988 (688763-1389501)	4061.62 (2777.53-5602.6)	-0.03 (-0.04--0.03)
Ireland	107038 (73928-146117)	4891.68 (3376.9-6679.93)	166792 (117450-228476)	4826.51 (3390.29-6620.18)	-0.08 (-0.09--0.06)
Israel	144781 (101517-195901)	5038.33 (3533.23-6817.23)	280433 (194093-385905)	4767.14 (3296.3-6566.58)	-0.12 (-0.14--0.1)
Italy	1984795 (1375535-2712819)	4849.06 (3361.28-6638.2)	2114689 (1463592-2880449)	4797.52 (3314.72-6559.71)	-0.08 (-0.1--0.07)
Jamaica	39126 (26642-54193)	3190.73 (2176.8-4417.29)	61973 (42382-85644)	3181.36 (2178.06-4396.23)	-0.03 (-0.05--0.01)
Japan	5315775 (3719602-7213780)	5769.73 (4028.68-7840.23)	4492810 (3142580-6119229)	5362.57 (3742.75-7316.79)	-0.16 (-0.19--0.13)
Jordan	73016 (50078-100453)	4131.84 (2836.4-5689.52)	321342 (220088-439324)	4097.37 (2806.03-5604.05)	-0.03 (-0.04--0.02)
Kazakhstan	444981 (310866-606138)	4523.57 (3168.86-6159.53)	580799 (401571-796986)	4573.6 (3159.95-6276.75)	0.01 (-0.02-0.04)
Kenya	366012 (252070-504726)	3985.4 (2757.03-5480.93)	991729 (683123-1366786)	3890.72 (2686.48-5348.88)	-0.09 (-0.11--0.07)
Kiribati	1201 (822-1669)	3242.19 (2226.55-4494.21)	2310 (1579-3191)	3301.55 (2258.98-4554.74)	0.11 (0.09-0.15)
Kuwait	41188 (28276-56855)	3909.17 (2688.78-5382.83)	153022 (105662-211599)	4094.32 (2815.62-5637.3)	0.15 (0.13-0.16)
Kyrgyzstan	107310 (74135-148563)	4594.37 (3184.01-6346.67)	185236 (127147-253086)	4477.03 (3075.96-6110.78)	-0.07 (-0.08--0.07)
Lao People's Democratic Republic	58023 (40053-80381)	3007.28 (2078.34-4166.73)	127509 (86766-177015)	2866.3 (1952.66-3970.96)	-0.17 (-0.19--0.15)
Latvia	95702 (66501-130844)	5006.72 (3477.77-6858.21)	68484 (47425-93291)	4947.44 (3420.19-6751.63)	-0.03 (-0.04--0.02)
Lebanon	69805 (47857-95942)	4054.77 (2779.91-5576.63)	151295 (103806-208016)	4050.46 (2780.37-5560.75)	0.04 (0.03-0.05)
Lesotho	22651 (15501-31413)	3288.21 (2252.79-4550.68)	31035 (21212-43056)	3116.99 (2134.09-4312.62)	-0.21 (-0.24--0.19)
Liberia	36862 (25166-51140)	3507.25 (2396.92-4860.38)	91058 (62078-126855)	3374.68 (2299.34-4680.89)	-0.15 (-0.17--0.13)

Libya	82812 (56637-114346)	4053.67 (2777.71-5599.26)	205113 (140927-284562)	4073.45 (2793.37-5646.19)	0 (-0.01-0)
Lithuania	131151 (90718-179186)	5121.59 (3547.3-7002.4)	103370 (71259-141484)	5025.97 (3458.99-6895.52)	-0.06 (-0.08--0.05)
Luxembourg	13465 (9285-18429)	4840.17 (3342.45-6625.12)	22880 (15914-31259)	4677.07 (3251.55-6403.15)	-0.09 (-0.1--0.09)
Madagascar	200129 (137519-276261)	3894.36 (2680.46-5367.99)	503985 (345301-702959)	3646.1 (2502.92-5074.91)	-0.24 (-0.26--0.22)
Malawi	154301 (107204-211271)	3732.04 (2604.59-5097.48)	322033 (220404-446836)	3663.71 (2511.4-5072.6)	-0.07 (-0.08--0.06)
Malaysia	267904 (190666-360174)	2853.81 (2029.62-3841.12)	588759 (400893-819434)	2743.34 (1868.91-3814.96)	-0.2 (-0.24--0.15)
Maldives	2639 (1785-3679)	2737.92 (1858.44-3808.39)	9795 (6704-13625)	2515.9 (1717.41-3491.59)	-0.27 (-0.32--0.23)
Mali	117742 (81417-162168)	3148.08 (2180.24-4327.04)	315904 (215298-440036)	3221.02 (2205.88-4463.49)	0.03 (0.01-0.06)
Malta	12494 (8676-17131)	4978.51 (3454.32-6824.46)	15133 (10573-20577)	4880.31 (3401.89-6650.07)	-0.09 (-0.1--0.08)
Marshall Islands	576 (393-799)	3137.15 (2141-4343.43)	1067 (735-1485)	3078.79 (2121.42-4279.68)	-0.06 (-0.07--0.06)
Mauritania	28890 (20125-39612)	3227.04 (2254.83-4416.73)	65885 (44758-91316)	3213.01 (2188.79-4445.43)	-0.06 (-0.09--0.04)
Mauritius	19725 (13656-26948)	3022.23 (2096.35-4126.79)	28050 (19116-39069)	2837.25 (1934.97-3957.08)	-0.15 (-0.19--0.11)
Mexico	1579749 (1075637-2187429)	3603.22 (2455.55-4981.54)	3282081 (2246712-4530877)	3767.32 (2578.34-5200.83)	0.11 (0-0.21)
Micronesia (Federated States of)	1495 (1020-2072)	3269.99 (2235.63-4519.16)	2117 (1451-2948)	3264.5 (2238.61-4547.75)	0 (-0.01-0.01)
Monaco	1043 (724-1432)	4753.89 (3297.3-6534.1)	1247 (862-1703)	4587.14 (3164.73-6288.2)	-0.09 (-0.11--0.08)
Mongolia	44555 (30737-61548)	4513.35 (3126.12-6209.07)	94671 (65647-129860)	4425.63 (3068.26-6063.35)	-0.08 (-0.09--0.07)
Montenegro	25129 (17597-34289)	6008.7 (4210.57-8207.18)	27322 (19092-37137)	6026.15 (4202.35-8213.32)	0.01 (0.01-0.03)
Morocco	610112 (426070-834871)	4585.8 (3206.03-6276.64)	1118555 (770280-1537448)	4505.37 (3101.87-6191.97)	-0.03 (-0.1-0.04)
Mozambique	230499 (157673-319203)	3883.69 (2661.13-5369.77)	508399 (348376-704299)	3825.63 (2627.78-5289.47)	-0.04 (-0.05--0.03)
Myanmar	545408 (372549-753900)	2564.36 (1754.37-3540.74)	939482 (639938-1294421)	2560.01 (1743.98-3527.08)	-0.05 (-0.07--0.02)
Namibia	20260 (14045-27783)	3264.86 (2269.51-4469.85)	43251 (29486-60209)	3247.24 (2216.37-4507.49)	0.01 (0-0.03)
Nauru	164 (112-228)	3244.51 (2215.82-4494.25)	196 (134-272)	3283.22 (2244.09-4552.04)	0.06 (0.04-0.09)
Nepal	482054 (336848-653636)	5153.13 (3610.47-6978.14)	902969 (620642-1245004)	4854.96 (3340.75-6684.49)	-0.14 (-0.2--0.08)
Netherlands	462093 (319802-629477)	4424.77 (3062.27-6026.32)	521652 (359931-711714)	4228.67 (2921.15-5778.51)	-0.1 (-0.13--0.08)
New Zealand	144998 (102106-197063)	6496.47 (4575.89-8826.56)	218430 (153151-296094)	6121.08 (4292.01-8304.7)	-0.11 (-0.13--0.08)
Nicaragua	68846 (47644-94928)	3990.65 (2758.38-5500.2)	162393 (112030-225284)	3930.72 (2712.31-5444.76)	-0.02 (-0.03--0.01)

Niger	110896 (75721-154337)	3482.01 (2383.68-4828.74)	333107 (227562-463505)	3519.53 (2409.56-4877.91)	0.07 (0.03-0.11)
Nigeria	1452072 (996893-2006272)	3523.75 (2425.39-4859.81)	3646822 (2498506-5041317)	3501.99 (2404.19-4827.3)	-0.02 (-0.06-0.03)
Niue	41 (28-56)	3210.33 (2207.88-4427.77)	38 (26-52)	3163.88 (2148.58-4386.86)	-0.04 (-0.05--0.02)
North Macedonia	75973 (52825-103258)	5732.61 (3987.17-7792.96)	96895 (67452-131907)	5679.84 (3947.84-7744.42)	-0.02 (-0.03--0.02)
Northern Mariana Islands	913 (625-1272)	3175.61 (2174.34-4405.13)	1178 (807-1620)	3154.78 (2155.62-4347.75)	-0.03 (-0.06-0)
Norway	132117 (91645-180335)	4725.14 (3278.04-6449.33)	171042 (118266-234063)	4455.28 (3081.34-6111.18)	-0.2 (-0.21--0.18)
Oman	39469 (26992-54399)	3919.4 (2680.03-5397.81)	126720 (87362-175313)	3855.67 (2655.65-5323.13)	-0.04 (-0.06--0.03)
Pakistan	1794056 (1189421-2523413)	3601.66 (2392.48-5054.56)	5024643 (3351343-7064784)	3956.1 (2647.06-5544.48)	0.39 (0.33-0.46)
Palau	283 (193-393)	3198.18 (2184.12-4427.9)	474 (325-654)	3114.31 (2135.1-4309.97)	-0.07 (-0.09--0.04)
Palestine	37471 (25529-51391)	4194.12 (2863.23-5757.11)	117161 (80176-162168)	4081.47 (2793.85-5646.5)	-0.07 (-0.08--0.07)
Panama	50658 (34660-69876)	3825.71 (2619.04-5267.71)	105185 (72199-145821)	3821.11 (2623.61-5297.2)	0 (-0.01-0.01)
Papua New Guinea	63242 (43161-87903)	3178.72 (2171.13-4406.63)	179570 (121999-247393)	3160.08 (2151.03-4344.64)	0 (-0.02-0.02)
Paraguay	77487 (54292-105178)	3859.39 (2704.09-5241.1)	178149 (122496-245665)	3939.21 (2709.84-5427.82)	0.06 (0-0.13)
Peru	323180 (220621-447619)	2906.13 (1987.26-4022.79)	703007 (482245-970698)	2986 (2049.29-4120.59)	0.13 (0.1-0.15)
Philippines	956865 (654211-1321584)	3073.51 (2105.51-4235.55)	2053974 (1405987-2834293)	2944.44 (2016.94-4059.49)	-0.17 (-0.19--0.15)
Poland	1649054 (1161981-2238073)	6419.03 (4519.67-8714.55)	1781051 (1256716-2415904)	6266.64 (4407.29-8513.68)	-0.09 (-0.11--0.08)
Portugal	351490 (242624-478728)	5074.08 (3502.78-6922.41)	385083 (269297-525209)	4949.53 (3450.36-6770.72)	-0.1 (-0.12--0.08)
Puerto Rico	69459 (47590-95835)	3104.02 (2126.28-4282.85)	72403 (49305-100332)	3085.58 (2101.75-4285.8)	-0.03 (-0.04--0.02)
Qatar	11321 (7751-15652)	3815.87 (2616.14-5248.07)	94232 (64643-130579)	3884.94 (2665.33-5363.64)	-0.01 (-0.03-0.01)
Republic of Korea	1450458 (1000154-1986834)	4965.47 (3427.38-6792.21)	1926441 (1331141-2643371)	4637.8 (3205.04-6368.11)	-0.17 (-0.2--0.14)
Republic of Moldova	148096 (102965-202723)	5091.29 (3539.44-6970.38)	143608 (99666-195723)	5001.22 (3468.51-6839.49)	-0.07 (-0.08--0.06)
Romania	1022648 (714857-1381195)	6347.85 (4435.04-8592.46)	837317 (581561-1140940)	6080.75 (4213.72-8308.95)	-0.16 (-0.18--0.15)
Russian Federation	5715543 (3989388-7777750)	5276.8 (3685.77-7188.83)	5651987 (3958138-7689772)	5095.53 (3555.74-6951.43)	-0.01 (-0.04-0.02)
Rwanda	119620 (81683-165789)	3973.3 (2719.74-5495.28)	262854 (180236-363055)	3850.69 (2643.09-5302.04)	-0.13 (-0.16--0.11)
Saint Kitts and Nevis	659 (447-918)	3125.82 (2126.92-4341.2)	1421 (963-1962)	3046.51 (2066.63-4208.83)	-0.09 (-0.09--0.08)
Saint Lucia	2228 (1524-3077)	3259.4 (2233.1-4501.09)	4276 (2923-5876)	3131.27 (2139.92-4308.75)	-0.13 (-0.13--0.13)

Saint Vincent and the Grenadines	1682 (1149-2333)	3095.18 (2118.61-4290.69)	2462 (1668-3412)	3053.72 (2069.59-4240.31)	-0.03 (-0.04--0.03)
Samoa	2634 (1793-3633)	3389.71 (2312.03-4671.35)	3735 (2554-5184)	3233.28 (2212.53-4487.79)	-0.2 (-0.22--0.17)
San Marino	796 (550-1094)	4779.75 (3307.35-6578.03)	1124 (776-1536)	4618.94 (3184.24-6338.71)	-0.08 (-0.09--0.07)
Sao Tome and Principe	1623 (1110-2249)	3197.91 (2196.25-4417.56)	3602 (2464-4997)	3088.06 (2113.93-4275.59)	-0.12 (-0.15--0.1)
Saudi Arabia	314566 (215737-433718)	3903.18 (2673.16-5374.61)	1151366 (789398-1596069)	3986.35 (2732.86-5508.31)	0.09 (0.08-0.09)
Senegal	103453 (71235-142435)	3323.12 (2292.18-4564.03)	245261 (167754-341042)	3197.8 (2192.07-4434.9)	-0.18 (-0.21--0.15)
Serbia	421726 (294719-573215)	6089.53 (4253.1-8292.74)	396416 (276372-541724)	6075.45 (4227.93-8317.42)	-0.01 (-0.02-0)
Seychelles	1113 (757-1550)	2807.82 (1914.44-3912.94)	2096 (1418-2888)	2655.81 (1796.66-3664.72)	-0.18 (-0.19--0.18)
Sierra Leone	67084 (45826-93131)	3595.11 (2460.83-4975.24)	143065 (97737-198888)	3392.92 (2322.7-4704.93)	-0.19 (-0.22--0.16)
Singapore	92450 (63957-125824)	4313.8 (2985.01-5864.34)	182613 (125824-249871)	3973 (2736.27-5446.26)	-0.13 (-0.19--0.07)
Slovakia	214122 (150510-288383)	6167.56 (4334.25-8307.98)	244424 (170086-333836)	5977.86 (4150.13-8181.17)	-0.09 (-0.09--0.08)
Slovenia	80956 (57166-109758)	5750.19 (4060.67-7800.45)	87177 (60737-118449)	5681.55 (3952.28-7743.18)	-0.06 (-0.07--0.05)
Solomon Islands	4698 (3213-6501)	3172.22 (2175.69-4385.49)	12067 (8256-16747)	3317.98 (2269.44-4594.61)	0.2 (0.18-0.21)
Somalia	121219 (83378-167627)	3691.55 (2542.56-5078.59)	321436 (219440-446363)	3688.04 (2526.17-5110.81)	-0.02 (-0.03-0)
South Africa	615049 (421297-850728)	3327.38 (2286.63-4589.67)	1140105 (782188-1573270)	3099.71 (2127.21-4272.82)	-0.2 (-0.21--0.19)
South Sudan	90860 (61813-126515)	3621.46 (2474.28-5030.89)	159578 (109304-221107)	3596.93 (2463.95-4975.35)	-0.03 (-0.05--0.02)
Spain	1173133 (874820-1517674)	4434.81 (3306.54-5741.96)	1376815 (932382-1925514)	4017.81 (2720.13-5617.65)	-0.06 (-0.18-0.05)
Sri Lanka	281694 (194474-386787)	2844.37 (1964.37-3900.18)	434209 (296390-600824)	2798.79 (1909.7-3876.08)	-0.06 (-0.07--0.04)
Sudan	391475 (267571-543065)	4158.5 (2841.27-5774.22)	968067 (662824-1336629)	4153.82 (2845.14-5737.23)	0 (-0.01-0)
Suriname	6784 (4622-9414)	3101.99 (2118.12-4300.58)	12547 (8585-17445)	3141.48 (2149.95-4373.64)	0.05 (0.04-0.06)
Sweden	159123 (114568-212291)	2743.9 (1973.47-3665.77)	255765 (170190-359481)	3629.76 (2415.72-5107.11)	0.93 (0.68-1.18)
Switzerland	264643 (196064-344563)	5437.42 (4024.78-7079.09)	320761 (221209-441082)	4883.64 (3366.37-6731.68)	-0.17 (-0.28--0.06)
Syrian Arab Republic	241760 (165325-332830)	4200.65 (2875.38-5784.52)	394707 (272054-541154)	4206.57 (2889.35-5782.99)	-0.02 (-0.03-0)
Taiwan (Province of China)	455704 (352052-563155)	3530.67 (2732.47-4361.43)	744631 (597243-876722)	3854.88 (3119.18-4529.98)	0.33 (0.27-0.39)
Tajikistan	107213 (73614-148140)	4377.39 (3019.29-6037.88)	249453 (171587-342770)	4270.22 (2943.97-5856.01)	-0.07 (-0.08--0.06)
Thailand	854713 (587163-1180646)	2506.23 (1723.51-3458.33)	1428356 (972455-1982825)	2615.75 (1781.86-3635.44)	0.19 (0.12-0.27)

Timor-Leste	10639 (7229-14700)	2831.63 (1929.98-3902.47)	18917 (12781-26204)	2696.3 (1826.82-3727.48)	-0.19 (-0.21--0.17)
Togo	53140 (36229-73963)	3621.69 (2478.67-5018.32)	145755 (99212-202366)	3424.33 (2335.25-4739.62)	-0.22 (-0.27--0.17)
Tokelau	27 (19-38)	3250.2 (2212.44-4515.03)	27 (19-38)	3181.45 (2177.2-4413.72)	-0.07 (-0.08--0.06)
Tonga	1563 (1070-2152)	3360.11 (2305.1-4626.45)	1875 (1282-2597)	3262.7 (2231.33-4519.96)	-0.11 (-0.12--0.09)
Trinidad and Tobago	21194 (14425-29482)	3147.57 (2143-4367.39)	31751 (21725-43908)	3122.99 (2134.07-4325.43)	-0.02 (-0.03--0.02)
Tunisia	183161 (127524-248851)	4081.54 (2845.25-5547.48)	345592 (237244-477125)	4168.22 (2860.5-5753.3)	0.05 (0.03-0.07)
Turkey	1405044 (1015857-1856501)	4326.82 (3126.81-5730.16)	2497299 (1720128-3461818)	4268.65 (2938.66-5918.17)	0.03 (-0.04-0.1)
Turkmenistan	79856 (54956-109966)	4471.93 (3094.26-6130.25)	144204 (99757-198153)	4354.72 (3014.1-5981.21)	-0.06 (-0.07--0.05)
Tuvalu	173 (118-241)	3299.04 (2248.6-4580.07)	240 (163-333)	3205.18 (2183.21-4435.21)	-0.09 (-0.11--0.08)
Uganda	263548 (180013-365277)	3834.75 (2630.99-5297.3)	677012 (464863-936236)	3715.14 (2561.51-5127.12)	-0.1 (-0.12--0.09)
Ukraine	2236587 (1556395-3041803)	5871.74 (4087.21-8017.37)	1946613 (1367784-2637619)	5741.26 (4023.28-7810.48)	-0.05 (-0.07--0.04)
United Arab Emirates	41598 (28743-57303)	3517.95 (2433.5-4831.39)	318242 (218873-440174)	3672.55 (2505.15-5069.88)	0.09 (0.04-0.14)
United Kingdom	1893759 (1324066-2577308)	4911.09 (3436.03-6687.84)	2259012 (1569144-3087965)	4733.56 (3287.48-6483.96)	-0.02 (-0.05-0.01)
United Republic of Tanzania	408783 (279606-565053)	3768.04 (2585.23-5195.27)	1006923 (683586-1394502)	3649.33 (2482.91-5040.8)	-0.08 (-0.09--0.07)
United States of America	10049670 (7145591-13526146)	5958.81 (4238.34-8014.56)	12554580 (9549066-15886818)	5438.44 (4138.58-6887.38)	-0.15 (-0.22--0.08)
United States Virgin Islands	2109 (1439-2917)	3121.4 (2127.59-4314.51)	1934 (1323-2670)	3102.69 (2126.39-4296.28)	-0.01 (-0.03-0)
Uruguay	94901 (66358-128746)	4783.21 (3347.54-6497.77)	117624 (81821-160015)	5069.56 (3525.31-6908.32)	0.18 (0.12-0.23)
Uzbekistan	454338 (313151-626819)	4518.67 (3121.73-6212.64)	1003682 (692615-1378913)	4480.09 (3091.32-6151.92)	0.01 (0-0.02)
Vanuatu	2358 (1610-3270)	3410.2 (2336.02-4718.38)	5797 (3957-8028)	3430.92 (2344.32-4746.36)	0.02 (0-0.04)
Venezuela (Bolivarian Republic of)	375599 (258094-516286)	3748.7 (2576.28-5142.83)	661485 (455191-911103)	3631.4 (2495.23-5002.15)	-0.12 (-0.13--0.11)
Viet Nam	975207 (670662-1342429)	2934.11 (2020.48-4035.55)	2061882 (1406276-2856326)	2885.79 (1966.63-3999.53)	-0.01 (-0.04-0)
Yemen	238872 (163981-329875)	4245.26 (2911.64-5864.58)	715466 (488124-988507)	4143.08 (2823.88-5731.7)	-0.1 (-0.11--0.09)
Zambia	102755 (71302-141393)	3191.36 (2217.6-4382.89)	305634 (208282-426281)	3414.39 (2335.68-4741.71)	0.09 (0.03-0.14)
Zimbabwe	139387 (96156-191719)	3332.49 (2311.13-4584.09)	261920 (178832-362532)	3508.64 (2399.39-4831.5)	0.19 (0.18-0.2)

Abbreviations: ASIR, age-standardized incidence rate; EAPC, estimated annual percentage change; UI, uncertainty interval; CI, confidence interval.

Table S3. The case number and ASR of prevalence of LBP in working-age group between 1990 and 2021 by countries/territories, with EAPC from 1990 to 2021.

Location	Cases in 1990 (95%UI)	ASPR (1/100,000) (95%UI)	Cases in 2021 (95%UI)	ASPR (1/100,000) (95%UI)	EAPC (95%CI)
Afghanistan	482935 (345071-653468)	9954.22 (7117.03-13469.1)	1427141 (1022338-1926989)	9936.2 (7112.36-13425.28)	-0.01 (-0.02-0)
Albania	283510 (204585-378224)	15285.5 (11046.29-20367.87)	305378 (221424-405558)	15519.16 (11243.5-20649.5)	0.04 (0.03-0.04)
Algeria	1192621 (855972-1614522)	9786.4 (7028.27-13265.69)	2740575 (1948430-3719412)	9693.46 (6887.28-13167.24)	-0.05 (-0.06--0.04)
American Samoa	1830 (1293-2490)	7576.05 (5359.06-10312.28)	2483 (1752-3358)	7457.5 (5266.57-10085.83)	-0.01 (-0.03-0)
Andorra	4381 (3159-5913)	11116.37 (8005.76-15002.65)	7663 (5439-10401)	10780.05 (7676.57-14615.88)	-0.08 (-0.09--0.07)
Angola	410794 (293958-555254)	9089.62 (6513.77-12262.39)	1233626 (878357-1669545)	8703 (6197.89-11770.69)	-0.17 (-0.2--0.13)
Antigua and Barbuda	2410 (1708-3274)	7103.82 (5032.84-9648.14)	4783 (3400-6498)	7097.85 (5042.38-9628.41)	0 (-0.02-0.01)
Argentina	2384067 (1719903-3207500)	11928.78 (8605.54-16049.39)	3608726 (2592512-4846810)	11776.96 (8461.38-15815.72)	-0.03 (-0.07-0.01)
Armenia	240365 (172464-322246)	11261.73 (8082.71-15090.78)	251288 (182281-336450)	11313.07 (8189.49-15155.24)	0.05 (0.04-0.06)
Australia	1716731 (1268091-2258957)	15238.86 (11254.8-20060.08)	2478659 (1788007-3316173)	13879.2 (10018.88-18561.75)	-0.2 (-0.24--0.16)
Austria	549924 (397862-741192)	10082.07 (7298.7-13577.92)	619531 (443752-842286)	9232.11 (6624.52-12501.3)	-0.05 (-0.13-0.02)
Azerbaijan	429831 (308220-578640)	10316.12 (7406.78-13865.39)	833493 (598072-1119939)	10525.43 (7547.11-14154.58)	0.11 (0.1-0.13)
Bahamas	10452 (7448-14266)	7114.99 (5070.17-9724.31)	20222 (14348-27559)	7105.72 (5043.35-9673.43)	-0.02 (-0.03--0.01)
Bahrain	28543 (20463-38427)	9358.78 (6692.2-12649.17)	108581 (77531-147427)	9159.46 (6532.1-12436.37)	-0.09 (-0.11--0.08)
Bangladesh	5533172 (4045350-7313629)	11564.09 (8478.54-15241.09)	11011863 (7951940-14828991)	10698.69 (7728.76-14403.18)	-0.19 (-0.25--0.12)
Barbados	11085 (7924-15031)	7226.59 (5165.7-9800.32)	16233 (11469-22218)	7176.98 (5078.08-9809.86)	-0.01 (-0.02--0.01)
Belarus	905066 (650673-1214573)	12212.12 (8769.69-16398.32)	895119 (649450-1194289)	12158.75 (8801.03-16269.57)	0 (-0.01-0)
Belgium	804645 (576941-1084240)	11420.54 (8199.48-15388.3)	909189 (648038-1229785)	11177.47 (7992.39-15102.74)	-0.06 (-0.07--0.04)
Belize	6093 (4325-8292)	7270.31 (5155.68-9899.89)	19578 (13910-26634)	7342.35 (5217.66-9997)	0.06 (0.04-0.07)
Benin	159285 (112891-215986)	8451.96 (5985.07-11468.24)	468438 (330901-637224)	8043.23 (5688.04-10953.93)	-0.17 (-0.2--0.14)
Bermuda	3126 (2218-4263)	7351.9 (5210.99-10024.44)	3596 (2534-4895)	7272.06 (5146.45-9876.91)	-0.04 (-0.05--0.03)
Bhutan	29441 (21181-39549)	10323.67 (7453.37-13864.86)	49450 (35438-66725)	10167.96 (7295.39-13719.58)	-0.04 (-0.04--0.03)
Bolivia (Plurinational State of)	221207 (157009-301334)	7180 (5085.76-9804.93)	524799 (371712-715720)	7242.36 (5128.82-9887.06)	0 (-0.03-0.04)
Bosnia and Herzegovina	438571 (319251-582148)	14001.6 (10188.3-18589.59)	369652 (266376-494949)	14574.87 (10493.04-19499.22)	0.13 (0.1-0.15)

Botswana	40492 (28797-55253)	7268.7 (5163.15-9914.67)	105045 (74705-143403)	7222.22 (5131.11-9863.12)	0 (-0.01-0.02)
Brazil	9074599 (6523833-12209638)	11002.49 (7909.38-14805.17)	17337059 (12488691-23321507)	11154.37 (8033.27-15003.61)	0.02 (0-0.04)
Brunei Darussalam	15936 (11439-21404)	10955.28 (7857.61-14747.79)	34802 (25003-46875)	10448.91 (7502.13-14071.85)	-0.13 (-0.15--0.11)
Bulgaria	951490 (690505-1261562)	15002.3 (10877.53-19912.59)	740159 (540358-980956)	14664.53 (10696.66-19441.47)	-0.06 (-0.06--0.05)
Burkina Faso	317113 (229954-424047)	8250.6 (5986-11040.85)	789957 (559065-1070423)	8070.78 (5706.45-10925.44)	-0.14 (-0.17--0.1)
Burundi	206817 (147316-279907)	9014.46 (6422.47-12212.68)	500536 (357789-677719)	8492.1 (6057.63-11526.98)	-0.23 (-0.24--0.21)
Cabo Verde	11656 (8277-15821)	7936.18 (5652.9-10751.45)	26945 (19124-36778)	7341.99 (5208.45-10024.2)	-0.28 (-0.32--0.24)
Cambodia	350920 (249450-479472)	7720.59 (5484.7-10552.53)	741149 (524693-1011427)	7056.35 (4991.57-9635.58)	-0.31 (-0.33--0.29)
Cameroon	392815 (280383-533899)	8772.37 (6270.53-11908.02)	1216851 (865963-1654605)	8298.01 (5910.04-11286.2)	-0.2 (-0.23--0.17)
Canada	2217072 (1601215-2974555)	11815.33 (8532.19-15852.67)	2878639 (2069327-3860392)	10812.02 (7787.79-14485.13)	-0.19 (-0.22--0.16)
Central African Republic	109934 (78379-148855)	8926.68 (6361.72-12073.42)	233874 (165929-317757)	8785.43 (6235.15-11924.8)	-0.07 (-0.08--0.06)
Chad	238629 (174508-316414)	9814.15 (7182.51-13004.2)	599670 (428033-816082)	8894.23 (6343.37-12121.73)	-0.27 (-0.36--0.17)
Chile	966979 (696396-1298432)	12089.82 (8705.7-16223.53)	1617940 (1164662-2165012)	11970.93 (8621.6-16016.03)	-0.04 (-0.08-0)
China	53976566 (38229185-73441772)	7564.29 (5361.07-10293.15)	68311808 (48740040-92641612)	6076.56 (4330.16-8238.33)	-0.5 (-0.62--0.38)
Colombia	1762763 (1251835-2377538)	9908.32 (7038.04-13389.96)	3394377 (2495426-4503091)	10001.64 (7352.5-13267.75)	0.05 (0.03-0.06)
Comoros	15822 (11464-21047)	7987.77 (5783.39-10627.46)	34547 (24556-46745)	8009.93 (5695.24-10837.9)	-0.05 (-0.08--0.02)
Congo	88325 (63420-119246)	8301.41 (5951.68-11220.17)	246910 (175734-335085)	8269.57 (5877.33-11231.95)	-0.04 (-0.06--0.02)
Cook Islands	774 (551-1052)	7457.93 (5311.07-10141.01)	986 (699-1342)	7652.87 (5444.18-10417.48)	0.11 (0.1-0.13)
Costa Rica	153487 (109877-207712)	9456.63 (6760.49-12832.11)	306840 (218516-414672)	9203.53 (6557.07-12435.98)	-0.07 (-0.08--0.07)
Coted'Ivoire	444343 (324495-593424)	8627.21 (6310.89-11522.79)	1110022 (785405-1514086)	8260.45 (5843.65-11263.21)	-0.12 (-0.17--0.07)
Croatia	529143 (393866-688690)	14670.99 (10902.76-19123.46)	442932 (320063-588088)	14201.38 (10264.79-18873)	-0.08 (-0.11--0.04)
Cuba	511716 (362353-695861)	7309.61 (5175.38-9940.53)	595355 (452351-775567)	6743.74 (5120.55-8785.87)	-0.11 (-0.15--0.06)
Cyprus	56092 (40160-75526)	11245.45 (8053.25-15142.08)	112863 (81147-151834)	11162.83 (8025.53-15011.32)	-0.05 (-0.07--0.03)
Czechia	1164044 (858517-1521138)	16234.95 (11956.9-21236.18)	1217849 (887362-1618123)	15789.62 (11481.79-21013.24)	-0.08 (-0.09--0.08)
Democratic People's Republic of Korea	1082544 (773381-1458151)	8195.29 (5859.61-11030.61)	1594113 (1125599-2177594)	7705.35 (5449.6-10512.86)	-0.22 (-0.24--0.2)
Democratic Republic of the Congo	1487675 (1063418-2019088)	9071.53 (6484.18-12308.62)	3702314 (2634067-5038197)	8731.44 (6217.13-11872.22)	-0.15 (-0.18--0.12)

Denmark	556732 (436062-693418)	15442.88 (12075.38-19259.46)	545400 (380172-747335)	13309.95 (9295.03-18181.93)	-0.75 (-0.89--0.62)
Djibouti	14955 (10666-20317)	7975.93 (5679.87-10823.04)	55849 (39684-75764)	7541.38 (5348.68-10224.5)	-0.2 (-0.22--0.19)
Dominica	2836 (2013-3845)	7449.68 (5287.14-10110.44)	3469 (2455-4723)	7179.14 (5087.02-9767)	-0.15 (-0.16--0.14)
Dominican Republic	261468 (188742-350242)	7130.23 (5146.3-9566.31)	520391 (369606-708512)	7345.01 (5216.34-10001.21)	0.08 (0.05-0.11)
Ecuador	344826 (250000-460884)	6854.7 (4972.84-9177.17)	713586 (528264-949309)	6322.51 (4680.07-8410.39)	-0.27 (-0.4--0.15)
Egypt	2850873 (2041973-3857527)	9782.98 (7010.7-13256.56)	6270610 (4496376-8439632)	10062.07 (7211.76-13553.46)	0.1 (0.06-0.14)
El Salvador	241142 (173165-326339)	9240.19 (6640.14-12528.28)	374758 (267409-507167)	9468.18 (6755.14-12816.83)	0.13 (0.11-0.14)
Equatorial Guinea	16597 (11803-22455)	8925.64 (6345.92-12075.45)	59658 (42394-81410)	8414.14 (5978.04-11468.3)	-0.22 (-0.24--0.2)
Eritrea	109269 (77397-149712)	7492.64 (5312.79-10263.86)	253247 (179977-343586)	7569.86 (5369.74-10263.62)	0.06 (0.05-0.08)
Estonia	135431 (99645-178142)	12070.19 (8869.2-15880.87)	113849 (82051-152446)	11943.19 (8600.45-15969.06)	0 (-0.02-0.02)
Eswatini	20629 (14836-27906)	6499.87 (4678.49-8788.21)	38189 (27128-52008)	6465.67 (4593.52-8798.52)	-0.07 (-0.11--0.03)
Ethiopia	1899742 (1360338-2551890)	9106.14 (6526.61-12222.73)	4192638 (2995215-5662882)	8389.99 (5991.87-11332.65)	-0.27 (-0.29--0.25)
Fiji	29876 (21193-40628)	7509 (5318.1-10220.17)	43151 (30474-58697)	7181.76 (5071.08-9770.12)	-0.13 (-0.14--0.13)
Finland	364383 (268575-482570)	10229.78 (7580.34-13483.41)	369111 (266875-496775)	9749.84 (7090.64-13039.3)	-0.09 (-0.11--0.07)
France	4343993 (3171521-5824028)	11101.55 (8106.37-14886.33)	5048508 (3624224-6809404)	11151.87 (8023.38-15022.2)	0 (-0.02-0.03)
Gabon	37997 (26925-51475)	8202.4 (5821.4-11108)	82143 (58659-111535)	8194.43 (5851.77-11117.88)	0 (0-0)
Gambia	30939 (21972-42072)	7700.29 (5476-10477.58)	81571 (57661-111432)	7482.54 (5276.57-10219.62)	-0.1 (-0.14--0.06)
Georgia	371396 (267948-495109)	9726.74 (7025.06-12983.51)	255048 (182935-344118)	9671.5 (6932.78-13053.22)	-0.11 (-0.16--0.06)
Germany	8034340 (5872596-10692430)	13674.16 (10000.48-18189.85)	8167541 (5921013-10879377)	13030.76 (9452.39-17335.88)	-0.07 (-0.1--0.05)
Ghana	496237 (360405-662860)	7523.99 (5457.89-10041.53)	1263945 (933175-1667730)	7191.57 (5308.82-9491.37)	-0.18 (-0.21--0.15)
Greece	782921 (564449-1038564)	10613.82 (7667.22-14084.25)	778910 (558037-1054522)	10524.25 (7535.22-14230.13)	-0.06 (-0.09--0.03)
Greenland	4217 (3032-5689)	11147.23 (8020.3-15034.06)	4480 (3204-6056)	10619.92 (7619.29-14334.19)	-0.08 (-0.11--0.04)
Grenada	3077 (2188-4169)	7243.72 (5153.06-9817.87)	5271 (3740-7193)	7132.18 (5066.27-9713.03)	-0.03 (-0.04--0.03)
Guam	5911 (4209-8037)	7326.62 (5211.89-9957.77)	8138 (5791-11075)	7272.94 (5178.88-9881.19)	-0.02 (-0.03-0)
Guatemala	404879 (297060-536074)	11031.33 (8094.25-14624.75)	964366 (690080-1301373)	10453.79 (7477.45-14122.23)	-0.1 (-0.15--0.05)
Guinea	221963 (158523-303104)	8357.2 (5966.74-11426.33)	481889 (343345-651983)	8272.78 (5887.79-11203.17)	-0.07 (-0.11--0.03)

Guinea-Bissau	32774 (23250-44619)	7995.95 (5668.61-10887.63)	72030 (51035-97939)	7809.81 (5536.94-10645.88)	-0.1 (-0.12--0.07)
Guyana	28718 (20466-38891)	7215.3 (5141.37-9782.62)	35182 (24825-47998)	7150.09 (5048.67-9750.57)	-0.03 (-0.04--0.03)
Haiti	225286 (159901-305672)	7238.54 (5135.93-9831.37)	534316 (379033-725919)	7192.33 (5095.94-9789.39)	-0.04 (-0.06--0.02)
Honduras	194065 (139102-261822)	9303.68 (6656.34-12568.32)	554985 (395104-749806)	9483.44 (6744.05-12825.41)	0.07 (0.07-0.08)
Hungary	1257141 (922589-1653203)	17023.52 (12463.35-22403.67)	1186787 (866529-1564155)	16704.84 (12202.96-22041.11)	-0.06 (-0.06--0.05)
Iceland	19388 (13943-26043)	12056.5 (8670.87-16203.66)	27655 (19876-37355)	11317.16 (8150.31-15273.95)	-0.23 (-0.25--0.22)
India	39122846 (27972883-52696447)	8731.45 (6244.53-11755.38)	68473989 (48796759-92518937)	7546.36 (5379.21-10194.56)	-0.49 (-0.61--0.37)
Indonesia	7197341 (5140382-9723585)	7345.75 (5239.92-9922.12)	14090216 (10074690-19029966)	7127.62 (5096.24-9626.06)	-0.05 (-0.07--0.04)
Iran (Islamic Republic of)	3229149 (2342484-4300989)	11974.35 (8681.17-15963.13)	6553801 (4725164-8797711)	10787.03 (7773.14-14496.93)	-0.27 (-0.32--0.22)
Iraq	835609 (596362-1128312)	9811.86 (7001.5-13251.54)	2385009 (1710384-3223497)	9628.76 (6895.58-13026)	-0.05 (-0.06--0.04)
Ireland	253132 (182606-340132)	11579.84 (8350.25-15567.47)	400805 (292185-536638)	11557.45 (8423.17-15468.11)	-0.08 (-0.11--0.05)
Israel	344333 (250864-456544)	12002.24 (8740.26-15929.55)	670786 (481085-903340)	11395.2 (8174.79-15344.77)	-0.13 (-0.16--0.1)
Italy	4694737 (3374123-6309630)	11436.17 (8219.94-15356.19)	5039890 (3619670-6785689)	11348.91 (8160.68-15270.71)	-0.09 (-0.11--0.07)
Jamaica	90237 (63918-123009)	7411.79 (5254.18-10118.66)	144365 (102745-195789)	7407.91 (5274.42-10044.87)	-0.03 (-0.05-0)
Japan	13037150 (9461053-17306216)	14083.37 (10228.87-18697.08)	10995450 (7946609-14664692)	12987.43 (9396.21-17305.74)	-0.19 (-0.22--0.15)
Jordan	174459 (125120-235912)	9900.55 (7101.57-13382.52)	765160 (546450-1033126)	9754.94 (6960.32-13182.04)	-0.04 (-0.05--0.03)
Kazakhstan	1032480 (749047-1372228)	10544.17 (7653.65-14012.64)	1368969 (982459-1846347)	10777.02 (7733.38-14532.73)	0.02 (-0.01-0.07)
Kenya	845205 (609400-1134184)	9337.56 (6736.06-12523.29)	2296001 (1649464-3087889)	9100.3 (6535.68-12237.35)	-0.1 (-0.12--0.09)
Kiribati	2744 (1945-3732)	7486.6 (5308.23-10183.73)	5353 (3810-7258)	7695 (5472.27-10433.39)	0.17 (0.13-0.19)
Kuwait	96833 (69682-129955)	9229.38 (6634.67-12429.27)	365561 (262028-496221)	9758.17 (6978.8-13245.52)	0.19 (0.17-0.2)
Kyrgyzstan	250318 (179185-337476)	10784.77 (7728.23-14514.36)	431087 (307921-582221)	10456.54 (7473.52-14127.78)	-0.1 (-0.1--0.09)
Lao People's Democratic Republic	133192 (95786-179550)	6965.14 (5007.8-9388.9)	294325 (208570-400270)	6658.5 (4719.21-9055.93)	-0.17 (-0.21--0.14)
Latvia	229602 (166020-308120)	11932.29 (8631.76-15999.31)	163722 (118015-220021)	11716.49 (8459.7-15719.71)	-0.06 (-0.07--0.05)
Lebanon	165700 (119046-224789)	9634.08 (6920.94-13081.24)	358811 (258065-485747)	9622.32 (6916.2-13033.66)	0.06 (0.03-0.08)
Lesotho	51212 (36347-69561)	7476.77 (5307.08-10155.71)	69855 (49573-95096)	7067.21 (5014.48-9617.64)	-0.23 (-0.26--0.2)
Liberia	83702 (59384-113930)	8051.73 (5706.1-10978.64)	208034 (148228-282417)	7769.94 (5541.51-10539.7)	-0.14 (-0.15--0.13)

Libya	196662 (140551-266012)	9658.35 (6892.27-13103.55)	488666 (347241-664520)	9682.91 (6877.23-13178.8)	-0.01 (-0.02-0)
Lithuania	315672 (227404-421876)	12277.67 (8848.1-16413.46)	248872 (178717-335342)	11966.63 (8610.21-16093.56)	-0.1 (-0.11--0.08)
Luxembourg	31891 (22979-42954)	11440.47 (8248.19-15416.15)	54501 (39299-73453)	11119.24 (8023.99-14969.79)	-0.09 (-0.1--0.08)
Madagascar	465195 (330026-632987)	9147.28 (6480.45-12449.86)	1159601 (823070-1576435)	8468.96 (6001.97-11501.93)	-0.28 (-0.31--0.26)
Malawi	353866 (257357-471639)	8658.91 (6296.67-11539.47)	739993 (525849-1003019)	8517.53 (6053.02-11547.7)	-0.07 (-0.09--0.06)
Malaysia	609805 (455893-796648)	6543.33 (4888.83-8561.34)	1351018 (950643-1848867)	6309.67 (4438.63-8636.37)	-0.2 (-0.26--0.15)
Maldives	5931 (4196-8142)	6215.99 (4399.09-8539.36)	22079 (15574-30161)	5704.79 (4011.05-7825.33)	-0.28 (-0.33--0.24)
Mali	261519 (188848-351892)	7036.4 (5084.75-9464.34)	709785 (502187-967294)	7307.13 (5180.78-9959.99)	0.08 (0.05-0.11)
Malta	29761 (21379-40032)	11854.93 (8511.14-15946.34)	36730 (26571-49019)	11790.76 (8516.47-15744.32)	-0.08 (-0.1--0.06)
Marshall Islands	1300 (926-1764)	7203.46 (5135.28-9777.86)	2430 (1719-3307)	7044.51 (4983.45-9591.32)	-0.06 (-0.07--0.06)
Mauritania	64499 (47160-86247)	7265.91 (5311.09-9723.92)	148535 (105126-202182)	7302.7 (5165.09-9940.24)	-0.05 (-0.08--0.01)
Mauritius	45669 (33117-61068)	7056.21 (5115.7-9437.79)	65541 (46302-89746)	6576.8 (4649.1-8988.71)	-0.17 (-0.22--0.12)
Mexico	3690222 (2625853-4996361)	8514.36 (6053.65-11548.04)	7747362 (5513423-10506925)	8884.48 (6323.11-12045.54)	0.1 (-0.01-0.22)
Micronesia (Federated States of)	3423 (2433-4664)	7599.04 (5396.39-10362.08)	4915 (3488-6676)	7593.18 (5392.26-10312.99)	0.02 (0-0.03)
Monaco	2459 (1764-3305)	11147.18 (8013.23-14991.23)	2953 (2112-3994)	10785.82 (7721.1-14562.06)	-0.1 (-0.11--0.09)
Mongolia	103250 (73996-138161)	10566.56 (7568.95-14126.22)	219172 (157220-294015)	10271.51 (7367.17-13776.55)	-0.11 (-0.12--0.1)
Montenegro	62220 (45018-82700)	14863.99 (10753.78-19762.13)	68944 (49833-91399)	15063.61 (10878.92-20001.04)	0.07 (0.06-0.08)
Morocco	1516374 (1108464-2023301)	11462.29 (8370.18-15309.21)	2770112 (1984353-3737425)	11154.75 (7988.35-15050.23)	-0.04 (-0.16-0.06)
Mozambique	537570 (382072-729411)	9131.41 (6495.15-12392.54)	1184485 (843091-1601639)	9027.81 (6426.61-12214.33)	-0.02 (-0.03--0.01)
Myanmar	1206898 (862176-1636942)	5730.47 (4094.34-7781.71)	2114524 (1496120-2892765)	5762.71 (4077.64-7883.48)	-0.03 (-0.06-0)
Namibia	45780 (33182-61444)	7440.52 (5395.35-9988.29)	99082 (70201-134456)	7493.34 (5305.01-10159.56)	0.05 (0.04-0.08)
Nauru	376 (267-510)	7518.71 (5338.39-10193.3)	452 (321-616)	7664.08 (5439.17-10443.2)	0.1 (0.06-0.13)
Nepal	1265119 (928171-1671526)	13689.36 (10056.08-18076.62)	2280727 (1644073-3068790)	12335.94 (8902.04-16589.71)	-0.24 (-0.34--0.14)
Netherlands	1063931 (763566-1432101)	10179.37 (7305.68-13700.78)	1201658 (858305-1628690)	9695.87 (6945.26-13106.17)	-0.13 (-0.15--0.11)
New Zealand	349992 (254976-463627)	15699.74 (11435.38-20802.29)	529988 (384814-700321)	14744.14 (10705.38-19477.2)	-0.12 (-0.15--0.1)
Nicaragua	162752 (116787-220358)	9552.19 (6852.01-12957.1)	388927 (279537-527339)	9447.18 (6788.06-12819.14)	0 (-0.01-0.01)

Niger	251736 (178974-340974)	7974.56 (5676.22-10777.64)	766350 (545288-1046168)	8195.96 (5836.33-11183.75)	0.14 (0.1-0.18)
Nigeria	3315603 (2374432-4478726)	8115.89 (5812.82-10968.34)	8352058 (5965162-11275679)	8096.9 (5786.13-10936.03)	0 (-0.05-0.04)
Niue	93 (67-127)	7405.68 (5290.95-10056.6)	88 (62-120)	7313.22 (5180.39-9966.26)	-0.04 (-0.05--0.02)
North Macedonia	183659 (132383-246298)	13852.35 (9984.87-18576.66)	236645 (171442-315853)	13761.43 (9955.43-18372.84)	-0.01 (-0.01-0)
Northern Mariana Islands	2088 (1480-2839)	7346.25 (5199.41-9997.62)	2759 (1955-3763)	7314.16 (5189.94-9969.03)	-0.04 (-0.07--0.01)
Norway	310284 (223155-415362)	11085.06 (7974.11-14845.13)	401749 (288169-540342)	10418.6 (7478.48-13999.71)	-0.21 (-0.23--0.2)
Oman	92434 (66036-124888)	9236.93 (6579.44-12528.82)	296270 (211797-399721)	9054.03 (6447.72-12245.95)	-0.05 (-0.07--0.04)
Pakistan	4084565 (2807193-5675380)	8274.12 (5696.18-11473.69)	11597046 (7954411-16128741)	9215.2 (6326.9-12810.85)	0.47 (0.38-0.55)
Palau	645 (457-876)	7368.33 (5212.25-10011.76)	1105 (783-1503)	7178.18 (5089.7-9755.9)	-0.06 (-0.08--0.03)
Palestine	89430 (64216-120613)	10061.62 (7222.63-13586.53)	277660 (199215-375846)	9697.72 (6947-13142.53)	-0.11 (-0.12--0.1)
Panama	118976 (84895-161190)	9049.2 (6447.72-12287.81)	250856 (178751-341835)	9110.26 (6494.67-12409.6)	0.03 (0.01-0.04)
Papua New Guinea	143914 (101833-195697)	7309.52 (5168.98-9941.79)	410991 (291823-557420)	7285.26 (5174.26-9887.4)	0.02 (0-0.04)
Paraguay	181177 (132594-240465)	9076.37 (6641.46-12058.24)	424350 (303654-573893)	9408.06 (6729.07-12734.99)	0.11 (0.03-0.19)
Peru	734053 (518286-1003183)	6641.27 (4684.81-9105.36)	1608709 (1140780-2188836)	6838.66 (4849.84-9304.33)	0.15 (0.11-0.17)
Philippines	2221520 (1590406-2996368)	7229.13 (5173.53-9747.27)	4797015 (3426348-6473295)	6903.78 (4931.85-9317.2)	-0.19 (-0.21--0.16)
Poland	4013363 (2931494-5282189)	15622.1 (11394.42-20590.87)	4331376 (3155819-5708612)	15119.88 (11001.51-19961.88)	-0.12 (-0.13--0.11)
Portugal	844289 (607946-1134342)	12152.37 (8750.94-16330.39)	934266 (680585-1246548)	11924.19 (8677.9-15900.33)	-0.12 (-0.14--0.09)
Puerto Rico	159847 (114325-216704)	7152.38 (5114.42-9698.13)	168474 (118809-230251)	7127.07 (5039.01-9733.71)	-0.03 (-0.04--0.01)
Qatar	26457 (18939-35706)	8963.83 (6388.93-12122.27)	221945 (158622-300654)	9171.11 (6535.49-12486.47)	-0.01 (-0.04-0.02)
Republic of Korea	3410453 (2457219-4577089)	11743.86 (8462.17-15778.01)	4586615 (3289783-6208326)	10930.81 (7849.49-14759.47)	-0.2 (-0.23--0.18)
Republic of Moldova	354455 (256308-476213)	12204.35 (8821.29-16397.55)	344884 (248374-464404)	11934.97 (8588.99-16087.52)	-0.1 (-0.12--0.09)
Romania	2631134 (1909764-3494129)	16240.69 (11780.58-21566.09)	2129834 (1532601-2840070)	15296.12 (11026.96-20395.23)	-0.24 (-0.26--0.22)
Russian Federation	13617663 (9882690-18088599)	12552.46 (9102.15-16687.43)	13454046 (9777722-17902148)	12074.39 (8758.33-16092.74)	0 (-0.04-0.03)
Rwanda	280485 (201105-378318)	9432.77 (6771.43-12723.49)	616651 (438957-836394)	9110.04 (6482.87-12368.77)	-0.15 (-0.17--0.12)
Saint Kitts and Nevis	1511 (1071-2056)	7215.94 (5115.16-9835.34)	3290 (2325-4487)	7018.23 (4960.02-9553.36)	-0.1 (-0.1--0.09)
Saint Lucia	5171 (3682-7041)	7630.54 (5427.57-10396.78)	9985 (7072-13685)	7274.26 (5155.4-9957.01)	-0.15 (-0.16--0.15)

Saint Vincent and the Grenadines	3848 (2740-5224)	7140.8 (5086.65-9703.5)	5706 (4041-7811)	7048.23 (4998.33-9652.07)	-0.03 (-0.03--0.02)
Samoa	6112 (4333-8261)	7963.69 (5650.6-10768.81)	8657 (6144-11835)	7523.67 (5343.56-10288.61)	-0.24 (-0.28--0.21)
San Marino	1875 (1351-2542)	11243.21 (8102.77-15235.28)	2662 (1903-3603)	10864.88 (7761.84-14684.66)	-0.09 (-0.1--0.09)
Sao Tome and Principe	3606 (2546-4904)	7157.91 (5054.33-9721.61)	8051 (5706-11033)	6939.74 (4915.85-9510.01)	-0.11 (-0.13--0.09)
Saudi Arabia	737985 (528704-999888)	9186.42 (6564.95-12501.28)	2730981 (1948084-3693988)	9454.33 (6721.56-12817.79)	0.12 (0.1-0.13)
Senegal	231230 (165678-310800)	7496.4 (5372.31-10080.14)	550463 (389609-751506)	7234.97 (5124.6-9874.52)	-0.19 (-0.22--0.15)
Serbia	1060257 (773947-1410150)	15222.1 (11099.45-20252.07)	1004357 (728278-1339680)	15266.95 (11053.82-20373.54)	0.01 (0-0.01)
Seychelles	2515 (1772-3417)	6402.43 (4511.82-8705.04)	4822 (3392-6562)	6073.83 (4273.04-8259.15)	-0.19 (-0.19--0.18)
Sierra Leone	153645 (109365-208292)	8319.75 (5920.74-11281.67)	326019 (231361-444587)	7815.25 (5536.22-10673.95)	-0.21 (-0.23--0.18)
Singapore	208997 (149937-281141)	9794.36 (7020.2-13192.09)	414091 (295684-562481)	8959.58 (6404.27-12156.55)	-0.14 (-0.21--0.08)
Slovakia	542378 (402725-710706)	15633.65 (11603.33-20500.02)	617498 (446715-825391)	14985.61 (10829.58-20023.82)	-0.12 (-0.13--0.11)
Slovenia	197937 (145252-261497)	14007.64 (10272.05-18509.11)	213527 (154697-286140)	13738.61 (9939.19-18404.03)	-0.07 (-0.09--0.05)
Solomon Islands	10683 (7590-14549)	7301.31 (5197.5-9943.62)	28005 (19920-38042)	7759.31 (5517.97-10549.58)	0.27 (0.25-0.3)
Somalia	276711 (197777-376132)	8518.57 (6084.03-11552.54)	740054 (528363-1000107)	8611.29 (6140.12-11654.05)	0 (-0.01-0.02)
South Africa	1403801 (1001160-1899687)	7658.51 (5467.34-10356.22)	2601847 (1851389-3516537)	7088.97 (5043.1-9588.84)	-0.21 (-0.23--0.2)
South Sudan	207435 (147529-280810)	8362.68 (5939.4-11324.23)	366859 (261982-497433)	8327.29 (5949.07-11288.78)	-0.03 (-0.04--0.01)
Spain	2533802 (1983643-3210919)	9561.95 (7480.73-12127.4)	2967502 (2083921-4076494)	8638.41 (6092.7-11854.96)	-0.06 (-0.18-0.06)
Sri Lanka	638078 (462393-860849)	6494.46 (4702.54-8763.14)	1002833 (706687-1363189)	6435.05 (4537.31-8740.86)	-0.04 (-0.06--0.03)
Sudan	935690 (667912-1268107)	9967.95 (7104.42-13526.59)	2303237 (1656708-3093624)	9912.89 (7112.41-13332.54)	-0.01 (-0.01-0)
Suriname	15589 (11017-21235)	7159.92 (5062.66-9753.19)	29205 (20663-39894)	7287.46 (5162.14-9943.43)	0.07 (0.05-0.09)
Sweden	362250 (274432-472349)	6268.91 (4738.36-8185.38)	576312 (396702-804649)	8161.97 (5614.9-11405.4)	0.86 (0.63-1.08)
Switzerland	664526 (535676-818753)	13643.14 (10977.8-16827.63)	779962 (557193-1053068)	11816.64 (8443.18-15918.58)	-0.23 (-0.4--0.07)
Syrian Arab Republic	579196 (414977-781078)	10102.52 (7236.33-13649.08)	949096 (678826-1279709)	10069.79 (7205.51-13587.1)	-0.03 (-0.05--0.01)
Taiwan (Province of China)	1048888 (888336-1234858)	8152.77 (6917.35-9579.83)	1831441 (1697905-1952602)	9354.74 (8652.62-9971.57)	0.52 (0.44-0.59)
Tajikistan	245749 (176467-331571)	10112.34 (7275.91-13666.65)	568781 (407700-767808)	9791.48 (7020.78-13217.02)	-0.1 (-0.1--0.09)
Thailand	1886291 (1349217-2556948)	5564.27 (3976.09-7557.2)	3266880 (2317906-4522944)	5917.46 (4201.68-8163.62)	0.27 (0.17-0.36)

Timor-Leste	24052 (17070-32643)	6481.4 (4593.18-8806.8)	42790 (30127-58558)	6160.51 (4344.15-8423.68)	-0.2 (-0.23--0.17)
Togo	121682 (86601-165324)	8401.19 (5985.67-11442.61)	333203 (237131-452061)	7880.72 (5615.04-10687.37)	-0.25 (-0.31--0.2)
Tokelau	63 (44-85)	7489.88 (5316.61-10201.56)	63 (44-85)	7350.25 (5195.33-9976.44)	-0.06 (-0.07--0.04)
Tonga	3624 (2580-4943)	7857 (5601.41-10703.65)	4351 (3097-5919)	7594.15 (5406.22-10325.29)	-0.14 (-0.15--0.12)
Trinidad and Tobago	48916 (34940-66841)	7304.28 (5217.68-9988.54)	73908 (52794-100846)	7235 (5165.06-9864.55)	-0.02 (-0.03--0.01)
Tunisia	436564 (318978-578423)	9765.26 (7138.18-12939.22)	832215 (594961-1123790)	10027.54 (7173.37-13538.64)	0.06 (0.03-0.08)
Turkey	3368992 (2586846-4334282)	10407.96 (7994.75-13404.22)	6018664 (4302457-8131428)	10272.89 (7348.2-13866.8)	0.05 (-0.04-0.14)
Turkmenistan	183968 (131756-248463)	10395.6 (7460.99-14027.02)	333494 (238457-452939)	10084.49 (7210.56-13692.04)	-0.07 (-0.08--0.06)
Tuvalu	399 (283-543)	7639.69 (5412.97-10385.03)	554 (392-755)	7407.8 (5245.43-10097.31)	-0.09 (-0.11--0.07)
Uganda	609324 (434112-827807)	8988.98 (6410.47-12209.41)	1561834 (1110450-2118872)	8683.1 (6177.26-11765.42)	-0.12 (-0.14--0.1)
Ukraine	5547812 (4035932-7355418)	14448.7 (10505.6-19180.94)	4776840 (3475029-6335335)	13963.63 (10125.27-18557.79)	-0.08 (-0.11--0.06)
United Arab Emirates	95241 (69053-127817)	8082.54 (5824.56-10888.6)	740909 (527953-1002641)	8512.95 (6043.88-11497.75)	0.11 (0.04-0.17)
United Kingdom	4521327 (3284677-6017248)	11680.26 (8488.66-15537.29)	5353338 (3854643-7175955)	11162.69 (8038.26-14951)	-0.06 (-0.09--0.02)
United Republic of Tanzania	938751 (670213-1265312)	8744.12 (6233.44-11791.09)	2313193 (1635413-3146651)	8462.07 (5991.45-11528.12)	-0.08 (-0.09--0.07)
United States of America	24256520 (18049433-31622705)	14388.98 (10700.23-18773.18)	30281196 (24806083-36591604)	13011.22 (10657.66-15724.03)	-0.15 (-0.23--0.07)
United States Virgin Islands	4872 (3467-6630)	7212.48 (5127.09-9813.32)	4532 (3204-6191)	7193.37 (5098.52-9804.16)	0 (-0.02-0.01)
Uruguay	220948 (161421-294592)	11108.54 (8114.49-14813.24)	280182 (200553-375196)	12009.23 (8605.11-16088.24)	0.22 (0.16-0.28)
Uzbekistan	1050020 (752368-1418877)	10542.02 (7561.8-14226.76)	2332477 (1671222-3138230)	10438.23 (7479.05-14049.38)	0.01 (0-0.04)
Vanuatu	5474 (3902-7422)	8018.34 (5719.35-10874.03)	13653 (9676-18473)	8137.99 (5770.13-11018.37)	0.05 (0.01-0.08)
Venezuela (Bolivarian Republic of)	873020 (625072-1175720)	8787.52 (6285.49-11859.83)	1548541 (1108897-2102261)	8468.67 (6064.85-11492.11)	-0.14 (-0.15--0.12)
Viet Nam	2211124 (1592553-2984118)	6746.05 (4863.79-9103.8)	4823821 (3390498-6581701)	6729.33 (4730.33-9181.84)	0.04 (0.02-0.07)
Yemen	574615 (411021-773090)	10252.39 (7325.57-13821.92)	1697904 (1214762-2298456)	9869.1 (7060.49-13355.11)	-0.15 (-0.16--0.13)
Zambia	225550 (162141-302542)	7074.42 (5097.89-9488.68)	688945 (488625-938204)	7779.46 (5519.55-10591.92)	0.15 (0.08-0.21)
Zimbabwe	316513 (228321-426043)	7649.59 (5536.71-10303.59)	611882 (435710-833474)	8279.58 (5894.06-11278.42)	0.29 (0.28-0.31)

Abbreviations: ASPR, age-standardized prevalence rate; EAPC, estimated annual percentage change; UI, uncertainty interval; CI, confidence interval.

Table S4. The case number and ASR of DALYs of LBP in working-age group between 1990 and 2021 by countries/territories, with EAPC from 1990 to 2021.

Location	Cases in 1990 (95%UI)	ASDR(1/100,000) (95%UI)	Cases in 2021 (95%UI)	ASDR(1/100,000) (95%UI)	EAPC (95%CI)
Afghanistan	53869 (33203-81287)	1113.19 (683.69-1677.25)	159216 (97122-242408)	1104.58 (675.07-1673.19)	-0.01 (-0.03-0)
Albania	32456 (19880-48898)	1750.3 (1075.6-2634.68)	34915 (21684-52254)	1776.37 (1103.46-2658.55)	0.05 (0.04-0.05)
Algeria	135476 (82858-205480)	1113.78 (684.21-1686.6)	311909 (189999-472439)	1101.84 (671.64-1670.69)	-0.05 (-0.07--0.04)
American Samoa	210 (127-319)	868.34 (526.39-1317.26)	281 (171-425)	845.37 (513.98-1278.36)	-0.04 (-0.06--0.02)
Andorra	503 (307-758)	1273.76 (780.38-1920.09)	877 (538-1330)	1230.86 (753.55-1870.32)	-0.08 (-0.09--0.08)
Angola	46345 (27905-70110)	1026.69 (621.93-1553.09)	139128 (85039-210348)	981.25 (603.04-1477.94)	-0.15 (-0.19--0.12)
Antigua and Barbuda	275 (165-416)	811.61 (488.23-1225.67)	544 (331-820)	807.07 (489.6-1217.04)	-0.01 (-0.03-0)
Argentina	271742 (166502-407584)	1360.22 (833.17-2040.24)	410709 (251786-618252)	1340.03 (821.04-2016.82)	-0.03 (-0.07-0.01)
Armenia	27533 (16796-41199)	1290.73 (789.82-1932.86)	28748 (17740-43083)	1295.08 (798.51-1941.01)	0.06 (0.05-0.07)
Australia	194814 (121636-289106)	1728.8 (1080.27-2566.46)	281841 (174999-421473)	1576.48 (978.57-2358.26)	-0.2 (-0.24--0.16)
Austria	62785 (38757-94386)	1150.76 (709.64-1729.04)	70888 (43546-106746)	1054.83 (647.09-1585.35)	-0.04 (-0.12-0.03)
Azerbaijan	49288 (30112-74058)	1183.97 (725.34-1780.39)	95579 (58414-142929)	1206.74 (737.97-1805.41)	0.12 (0.1-0.14)
Bahamas	1194 (718-1822)	813.03 (491.21-1238.95)	2299 (1390-3473)	807.78 (488.11-1220.05)	-0.02 (-0.03--0.01)
Bahrain	3267 (1985-4962)	1067.33 (653.07-1621.24)	12371 (7516-18624)	1039.66 (632.37-1565.88)	-0.09 (-0.1--0.08)
Bangladesh	625430 (388006-930871)	1307.4 (816.06-1943)	1247861 (767777-1878082)	1211.82 (746.6-1822.02)	-0.17 (-0.24--0.1)
Barbados	1268 (770-1908)	826.92 (503.23-1244.21)	1847 (1131-2793)	817.41 (498.88-1237.74)	-0.02 (-0.03--0.02)
Belarus	102973 (63579-154168)	1391.16 (858.04-2086.13)	101655 (62754-152241)	1383.17 (851-2077.65)	0 (-0.01-0.01)
Belgium	91953 (56451-137875)	1304.73 (800.4-1958.36)	103552 (63614-155756)	1272.71 (781.55-1913.63)	-0.06 (-0.07--0.05)
Belize	696 (419-1049)	832.29 (503.56-1257.13)	2227 (1348-3404)	835.28 (506.72-1276.34)	0.04 (0.03-0.06)
Benin	17969 (10885-27103)	953.88 (580.84-1435.25)	53275 (32075-81159)	914.59 (554.58-1393.88)	-0.14 (-0.17--0.11)
Bermuda	359 (218-544)	843.69 (512.81-1276.81)	412 (253-626)	833.69 (508.85-1265.23)	-0.04 (-0.05--0.03)
Bhutan	3341 (2055-5014)	1171.86 (725.87-1754.46)	5641 (3467-8479)	1158.27 (714.69-1739.4)	-0.02 (-0.03--0.01)
Bolivia (Plurinational State of)	25149 (15149-38200)	816.92 (494.74-1240.83)	59622 (36134-90828)	822.42 (499.31-1252.21)	0.01 (-0.03-0.05)
Bosnia and Herzegovina	50039 (31202-74127)	1598.03 (996.26-2368.44)	42024 (26155-62582)	1660.7 (1030.76-2478.62)	0.13 (0.11-0.16)

Botswana	4594 (2775-6914)	826.03 (501.91-1243.91)	11780 (7129-17800)	808.49 (491.13-1220.78)	-0.02 (-0.04--0.01)
Brazil	1022345 (625489-1535151)	1239.27 (759.67-1860.61)	1951785 (1203591-2914640)	1255.3 (773.68-1875.17)	0.03 (0.01-0.04)
Brunei Darussalam	1836 (1112-2759)	1260.56 (768.04-1889.7)	4005 (2441-6072)	1200.22 (732.46-1818.78)	-0.13 (-0.16--0.11)
Bulgaria	108504 (66823-161886)	1712.99 (1052.42-2559.33)	84340 (52533-126154)	1672.83 (1037.47-2503.65)	-0.05 (-0.06--0.04)
Burkina Faso	35800 (22180-53380)	932.65 (580.25-1389.72)	90094 (54690-136847)	921.62 (562.24-1400.86)	-0.1 (-0.13--0.06)
Burundi	23468 (14196-35574)	1023.25 (624.09-1546.76)	56818 (34225-85837)	964.84 (584.76-1460.34)	-0.21 (-0.23--0.2)
Cabo Verde	1331 (807-2018)	907.91 (553.52-1376.25)	3078 (1879-4658)	838.14 (512.79-1267.65)	-0.28 (-0.32--0.24)
Cambodia	39880 (24118-60397)	878.69 (534.12-1328.46)	84892 (51643-129048)	807.8 (492-1228.38)	-0.28 (-0.3--0.26)
Cameroon	44364 (26966-67326)	991.66 (605.45-1503.95)	138278 (83632-209821)	942.87 (572.84-1426.32)	-0.18 (-0.21--0.15)
Canada	254290 (156984-381286)	1353.93 (836.1-2029.67)	328830 (202705-496108)	1234.73 (759.16-1861.23)	-0.2 (-0.23--0.17)
Central African Republic	12268 (7550-18475)	996.24 (616.38-1497.82)	26239 (15907-39674)	985.32 (598.85-1490)	-0.04 (-0.05--0.03)
Chad	27016 (16723-40309)	1112.17 (690.51-1653.81)	67962 (41065-102498)	1008.95 (614.29-1516.63)	-0.26 (-0.36--0.17)
Chile	109787 (67246-164801)	1372.67 (843.03-2057.05)	183454 (112643-274830)	1357.25 (833.03-2031.54)	-0.04 (-0.08-0)
China	6225364 (3763691-9406580)	872.65 (529.03-1318.05)	7893310 (4825939-11911828)	702.07 (427.71-1061.07)	-0.49 (-0.61--0.37)
Colombia	201256 (122116-304828)	1131.86 (689.16-1710.35)	387794 (239197-577252)	1142.58 (704.77-1700.34)	0.06 (0.04-0.08)
Comoros	1798 (1106-2674)	908.99 (561.79-1351.52)	3941 (2405-5951)	913.46 (559.21-1378.6)	-0.03 (-0.05-0)
Congo	9942 (6069-14939)	935.84 (575.53-1404.34)	27880 (16916-42040)	933.27 (567.37-1407.42)	-0.02 (-0.04--0.01)
Cook Islands	89 (54-135)	856.38 (518.75-1306.54)	112 (68-170)	871.74 (529.57-1321.16)	0.09 (0.08-0.11)
Costa Rica	17558 (10620-26360)	1082.08 (657.04-1625.27)	34948 (21342-52536)	1048.01 (640.04-1575.47)	-0.08 (-0.09--0.08)
Coted'Ivoire	49928 (30887-74161)	969.13 (603.54-1439.19)	126166 (76075-192022)	938.58 (568.16-1427.83)	-0.08 (-0.13--0.03)
Croatia	60487 (37942-88806)	1678.12 (1050.03-2466.29)	50655 (31373-75718)	1626.51 (1003.68-2434.57)	-0.06 (-0.1--0.03)
Cuba	58468 (35583-88969)	835.85 (508.6-1270.69)	67973 (43072-99187)	770.18 (486.36-1124.72)	-0.1 (-0.15--0.05)
Cyprus	6424 (3930-9728)	1287.52 (787.92-1949.42)	12940 (8007-19537)	1277.49 (789.03-1930.65)	-0.05 (-0.07--0.03)
Czechia	132779 (82943-197001)	1852.94 (1156.07-2750.96)	139007 (86505-207605)	1802.63 (1117.71-2694.57)	-0.08 (-0.08--0.07)
Democratic People's Republic of Korea	125128 (75802-189545)	947.28 (574.4-1433.11)	184716 (111908-281672)	892.73 (538.95-1362.01)	-0.21 (-0.23--0.19)
Democratic Republic of the Congo	165533 (101112-250291)	1010.44 (618.01-1528.94)	417377 (254784-631346)	984.82 (603.54-1485.67)	-0.1 (-0.12--0.07)

Denmark	63528 (41742-91094)	1761.53 (1154.94-2527.42)	62348 (37366-95862)	1520.68 (914.08-2337.24)	-0.74 (-0.88--0.61)
Djibouti	1708 (1033-2580)	912.46 (554.47-1373.01)	6393 (3875-9672)	862.48 (524.26-1303.69)	-0.2 (-0.21--0.18)
Dominica	323 (197-489)	850.59 (518.66-1287.58)	394 (241-593)	816.16 (499.74-1229.33)	-0.15 (-0.17--0.14)
Dominican Republic	29819 (18158-44854)	814.89 (498.03-1226.18)	59198 (36076-90078)	835.21 (509.57-1270.09)	0.07 (0.05-0.09)
Ecuador	39388 (24192-59260)	783.86 (482.62-1181.47)	81358 (50941-120364)	720.72 (451.57-1065.92)	-0.28 (-0.4--0.16)
Egypt	324291 (198855-488057)	1114.51 (684.9-1676.53)	712866 (434541-1072876)	1143.63 (697.68-1722.05)	0.1 (0.06-0.15)
El Salvador	27315 (16614-41004)	1049.54 (639.11-1574.64)	42588 (25934-64805)	1075.89 (655.97-1635.7)	0.13 (0.12-0.15)
Equatorial Guinea	1855 (1138-2795)	998.27 (614.52-1502.15)	6734 (4083-10174)	948.47 (579.33-1433.75)	-0.18 (-0.2--0.16)
Eritrea	12306 (7455-18629)	846.1 (515.39-1279.36)	28727 (17406-43487)	856.69 (520.65-1294.79)	0.08 (0.06-0.09)
Estonia	15372 (9523-22800)	1371.54 (848.9-2036.67)	12964 (8018-19493)	1360.92 (840.22-2043.9)	0.02 (0-0.04)
Eswatini	2346 (1436-3526)	740.29 (456.56-1111.63)	4252 (2588-6452)	718.72 (439.44-1087.99)	-0.16 (-0.2--0.11)
Ethiopia	214495 (130968-321574)	1028.32 (631.11-1538.82)	476337 (290131-715121)	952.96 (583.06-1426.53)	-0.24 (-0.25--0.22)
Fiji	3420 (2074-5173)	858.91 (521.75-1297.74)	4913 (2966-7443)	817.9 (493.55-1239.17)	-0.14 (-0.14--0.13)
Finland	41632 (26116-61661)	1167.83 (733.65-1727.37)	42132 (26184-63219)	1112.84 (691.18-1665.24)	-0.09 (-0.11--0.07)
France	494981 (307766-743010)	1264.91 (786.33-1900.56)	574903 (354383-865093)	1269.44 (779.6-1913.08)	0 (-0.02-0.03)
Gabon	4280 (2582-6417)	924.87 (560.59-1385.49)	9252 (5657-13996)	922.9 (565.26-1395.35)	0 (0-0.01)
Gambia	3514 (2134-5300)	876.15 (536.24-1318.03)	9240 (5568-13974)	847.52 (513.89-1280.61)	-0.11 (-0.15--0.07)
Georgia	42571 (26014-63294)	1116.17 (680.85-1661.62)	29046 (18048-43609)	1103.25 (682.6-1655.53)	-0.12 (-0.17--0.07)
Germany	917518 (573102-1373124)	1559.85 (973.23-2336.77)	931201 (582779-1396647)	1485.12 (925.16-2231.24)	-0.08 (-0.1--0.05)
Ghana	56286 (34748-83800)	854.54 (530.48-1270.05)	144071 (89047-214571)	819.6 (510.34-1218.65)	-0.17 (-0.2--0.14)
Greece	89397 (55301-133317)	1212.11 (748.16-1805.33)	88908 (54763-133269)	1199.96 (735.76-1804.19)	-0.06 (-0.09--0.03)
Greenland	479 (292-724)	1263.98 (773.39-1910.8)	509 (311-770)	1205.88 (737.75-1822.75)	-0.07 (-0.1--0.03)
Grenada	350 (210-531)	824.57 (498.05-1251.35)	598 (365-905)	809.82 (493.58-1225.02)	-0.04 (-0.04--0.03)
Guam	683 (414-1037)	846.33 (515.29-1282.23)	935 (578-1411)	836.97 (516.72-1262.93)	-0.03 (-0.04--0.01)
Guatemala	45560 (28294-67502)	1243.89 (775.19-1842.9)	108797 (66196-163450)	1179.04 (718.71-1771.47)	-0.09 (-0.14--0.04)
Guinea	25190 (15346-37847)	949.18 (579.8-1424.81)	54793 (33073-82489)	941.05 (571.92-1417.95)	-0.06 (-0.1--0.02)

Guinea-Bissau	3703 (2241-5615)	904.05 (550.02-1368.41)	8171 (4927-12368)	885.58 (537.53-1338.11)	-0.08 (-0.11--0.05)
Guyana	3237 (1963-4896)	812.64 (495.5-1226.58)	3939 (2414-5974)	800.64 (490.87-1212.62)	-0.04 (-0.05--0.04)
Haiti	25261 (15302-38104)	812.44 (494.08-1227.15)	60025 (36766-91055)	807.27 (495.76-1225.99)	-0.03 (-0.06--0.01)
Honduras	22096 (13273-33476)	1061.04 (640.39-1609.25)	62971 (38439-95148)	1075.66 (658.02-1622.37)	0.07 (0.06-0.08)
Hungary	142931 (89252-212435)	1937.43 (1208.16-2886.55)	135598 (84905-201583)	1910.13 (1191.92-2844.71)	-0.03 (-0.04--0.03)
Iceland	2219 (1364-3345)	1380.29 (849.37-2078.98)	3170 (1949-4786)	1295.96 (795.93-1954.3)	-0.23 (-0.24--0.22)
India	4392500 (2695202-6570556)	979.65 (603.11-1463.83)	7702009 (4714781-11568428)	848.26 (519.97-1273.22)	-0.48 (-0.6--0.36)
Indonesia	826439 (502404-1246294)	843.65 (514.6-1271.06)	1621657 (986468-2443824)	819.88 (498.44-1235.68)	-0.04 (-0.06--0.02)
Iran (Islamic Republic of)	365084 (225474-544676)	1356.55 (839.97-2023.12)	741534 (458230-1114327)	1218.32 (752.8-1832.46)	-0.28 (-0.33--0.22)
Iraq	94028 (57194-141597)	1106.65 (676.19-1664.5)	268131 (163753-403221)	1080.8 (662.21-1624.7)	-0.06 (-0.07--0.05)
Ireland	28880 (17845-43693)	1322.14 (816.77-2001.05)	45749 (28151-68269)	1317.46 (809.58-1967.67)	-0.08 (-0.11--0.06)
Israel	39455 (24524-58610)	1376.4 (856.81-2046.32)	76849 (47103-116049)	1305.45 (799.37-1971.93)	-0.13 (-0.16--0.1)
Italy	534362 (329537-800220)	1302.09 (801.92-1950.59)	576460 (355222-862016)	1296.78 (796.91-1940.63)	-0.07 (-0.08--0.05)
Jamaica	10325 (6231-15702)	849.62 (514.13-1292.6)	16474 (9976-24856)	845.24 (511.49-1275.16)	-0.03 (-0.05--0.01)
Japan	1503289 (930475-2245566)	1623.46 (1003.36-2424.63)	1270641 (785195-1906588)	1498.82 (923.61-2246.91)	-0.18 (-0.21--0.15)
Jordan	19819 (11995-30109)	1127.64 (683.6-1707.33)	86847 (53353-131228)	1106.48 (680.93-1670.16)	-0.05 (-0.06--0.04)
Kazakhstan	117633 (72183-175420)	1201.38 (738.74-1788.35)	156013 (95789-234226)	1227.9 (752.99-1843.61)	0.03 (-0.01-0.08)
Kenya	95858 (58797-143847)	1059.78 (653.71-1587.16)	260569 (159471-390897)	1032.27 (634.7-1547.83)	-0.08 (-0.09--0.07)
Kiribati	311 (188-471)	848.87 (513.32-1283.48)	608 (369-920)	873.93 (531.71-1321.25)	0.17 (0.14-0.21)
Kuwait	11108 (6776-16770)	1056.43 (646.61-1594.67)	41653 (25319-62517)	1107.38 (673.73-1664.92)	0.16 (0.14-0.18)
Kyrgyzstan	28566 (17504-42937)	1231.84 (757.13-1852.02)	49362 (30459-74622)	1196.9 (739.24-1808.93)	-0.08 (-0.08--0.07)
Lao People's Democratic Republic	15223 (9275-22829)	796.9 (487.16-1194.14)	33888 (20516-51333)	766.25 (465.55-1159.85)	-0.14 (-0.18--0.11)
Latvia	26020 (16121-39068)	1354.32 (838.54-2036.04)	18599 (11405-28092)	1334.03 (815.94-2012.46)	-0.03 (-0.04--0.02)
Lebanon	18740 (11447-28318)	1090.91 (666.69-1648.37)	40464 (24800-60918)	1083.03 (665.14-1631.85)	0.05 (0.03-0.07)
Lesotho	5813 (3554-8756)	849.44 (521.12-1278.62)	7749 (4693-11703)	782.83 (477.19-1180.08)	-0.31 (-0.34--0.28)
Liberia	9297 (5645-14036)	895.15 (546.27-1350.02)	23231 (14111-35165)	866.64 (528.58-1309.27)	-0.12 (-0.13--0.11)

Libya	22358 (13633-33767)	1100.96 (673.87-1660.8)	55319 (33885-83724)	1094.41 (669.36-1656.39)	-0.03 (-0.04--0.02)
Lithuania	35824 (22200-53877)	1394.85 (863.64-2098.44)	28211 (17495-42298)	1359.75 (840.9-2035.42)	-0.08 (-0.1--0.07)
Luxembourg	3636 (2229-5465)	1303.34 (798.06-1961.26)	6234 (3822-9381)	1269.86 (777.7-1911.92)	-0.08 (-0.09--0.07)
Madagascar	52640 (31811-79763)	1036.98 (630.6-1567.52)	131900 (79436-200381)	964.35 (583.91-1461.02)	-0.26 (-0.28--0.23)
Malawi	39803 (24635-59500)	976.21 (608.76-1457.12)	83989 (50838-128397)	967.28 (588.75-1477.36)	-0.03 (-0.04--0.01)
Malaysia	70032 (44162-102611)	751.32 (474.86-1101.25)	155340 (93175-237036)	725.03 (435.43-1105.76)	-0.2 (-0.25--0.14)
Maldives	677 (407-1033)	711.03 (428.74-1082.82)	2553 (1531-3852)	656.95 (396.11-993.04)	-0.26 (-0.3--0.21)
Mali	29567 (18118-44198)	796.1 (488.73-1190.63)	80491 (48486-122026)	829.28 (502.44-1256.97)	0.1 (0.07-0.12)
Malta	3413 (2103-5156)	1358.36 (837.22-2053.37)	4202 (2625-6285)	1347.38 (839.1-2016.31)	-0.08 (-0.11--0.06)
Marshall Islands	149 (90-224)	823.36 (500.58-1239.48)	276 (168-416)	798.61 (487.45-1203.53)	-0.09 (-0.1--0.09)
Mauritania	7348 (4565-11052)	828.49 (517.09-1245.34)	16950 (10281-25512)	834.05 (507.59-1254.88)	-0.03 (-0.06-0)
Mauritius	5221 (3222-7836)	806.23 (498.17-1210.05)	7471 (4560-11270)	750.69 (457.16-1133.06)	-0.17 (-0.23--0.12)
Mexico	418724 (254469-630394)	966.05 (589.4-1454.62)	877947 (537081-1320290)	1006.71 (615.64-1513.7)	0.09 (-0.02-0.21)
Micronesia (Federated States of)	391 (237-595)	868.85 (528.84-1322)	560 (339-843)	865.45 (523.89-1303.28)	0.01 (-0.01-0.02)
Monaco	282 (173-429)	1277.9 (782.87-1940.87)	337 (206-508)	1230.83 (750.14-1858.08)	-0.11 (-0.11--0.1)
Mongolia	11786 (7215-17648)	1207.05 (742.37-1804.42)	25024 (15456-37545)	1171.28 (724.07-1757.65)	-0.11 (-0.12--0.1)
Montenegro	7135 (4408-10642)	1705.19 (1053.69-2545.82)	7865 (4882-11746)	1720.64 (1064.37-2568.45)	0.07 (0.05-0.08)
Morocco	171829 (106860-257119)	1300.33 (812.03-1949.77)	312228 (192418-467889)	1257.22 (774.74-1884.13)	-0.06 (-0.17-0.05)
Mozambique	60253 (36639-90827)	1024.59 (625.43-1542.98)	132195 (81290-198706)	1008.28 (623.05-1516.46)	-0.02 (-0.04-0)
Myanmar	137876 (83918-208551)	655.3 (400.65-991.73)	242494 (147575-365791)	661 (402.4-997.06)	-0.01 (-0.04-0.02)
Namibia	5200 (3205-7823)	847.01 (524.02-1272.14)	11175 (6792-16951)	844.34 (515.34-1278.52)	0.04 (0.01-0.06)
Nauru	43 (26-65)	861.1 (523.69-1300.31)	51 (31-78)	870.63 (529.38-1322.82)	0.08 (0.05-0.11)
Nepal	142240 (89176-212641)	1538.64 (968.34-2296.67)	257106 (158272-386061)	1389.96 (857.22-2085.06)	-0.22 (-0.33--0.12)
Netherlands	122096 (74529-183697)	1167.52 (712.56-1756.31)	137612 (84940-208205)	1109.63 (684.24-1674.31)	-0.13 (-0.15--0.11)
New Zealand	39454 (24539-58779)	1770.09 (1100.98-2636.75)	60170 (37401-90047)	1672.74 (1038.69-2502.92)	-0.11 (-0.13--0.08)
Nicaragua	18467 (11230-27664)	1087.19 (664.03-1629.7)	44200 (26868-66536)	1072.57 (653.4-1613.28)	0 (-0.01-0.01)

Niger	28530 (17298-42995)	905.18 (551.36-1361.96)	87279 (52846-132803)	934.72 (570.29-1422.46)	0.16 (0.13-0.2)
Nigeria	374133 (229056-561346)	916.75 (563.28-1373.91)	947466 (576925-1421940)	919.03 (562.1-1377.14)	0.02 (-0.02-0.07)
Niue	11 (7-16)	850.4 (517.5-1289.29)	10 (6-15)	833.08 (505.72-1254.67)	-0.06 (-0.07--0.04)
North Macedonia	21013 (12929-31685)	1585.24 (975.44-2391.14)	27005 (16649-40313)	1571.32 (965.73-2347.73)	0 (-0.01-0)
Northern Mariana Islands	241 (146-369)	847.37 (514.85-1290.61)	316 (192-480)	839.46 (508.02-1277.1)	-0.05 (-0.08--0.02)
Norway	35364 (21743-53141)	1263.23 (776.5-1898.47)	45991 (28288-68933)	1191.59 (731.37-1789.01)	-0.19 (-0.21--0.18)
Oman	10569 (6426-16040)	1053.27 (643.67-1599.03)	33961 (20612-51293)	1031.78 (629.29-1561.3)	-0.05 (-0.06--0.03)
Pakistan	461564 (274380-711189)	935.48 (559.31-1440.61)	1310155 (779797-2021569)	1040.01 (621.89-1604.27)	0.47 (0.38-0.55)
Palau	74 (45-112)	844.29 (517.96-1280.14)	126 (76-191)	817.45 (494.75-1244.22)	-0.08 (-0.1--0.06)
Palestine	10117 (6198-15301)	1141.65 (702.16-1725.84)	31338 (19013-47691)	1094.07 (666.56-1664.14)	-0.12 (-0.13--0.11)
Panama	13618 (8235-20690)	1036.77 (628.29-1574.24)	28610 (17440-43175)	1039.09 (633.54-1567.38)	0.02 (0-0.04)
Papua New Guinea	16360 (9931-24713)	831.24 (506.57-1253.19)	46900 (28251-70367)	830.43 (501.89-1244.98)	0.03 (0-0.04)
Paraguay	20599 (12910-30628)	1033.09 (647.84-1535.75)	48044 (29222-72479)	1064.84 (648.35-1607.02)	0.1 (0.02-0.18)
Peru	83819 (50606-127487)	759.48 (460.01-1154.62)	183898 (110329-278599)	781.37 (469.23-1183.45)	0.16 (0.12-0.19)
Philippines	253744 (154694-381117)	825.95 (505.02-1239.58)	549194 (334080-826985)	790.27 (481.27-1189.51)	-0.17 (-0.2--0.15)
Poland	455909 (283734-676130)	1775.36 (1105.09-2638.13)	494317 (307600-734428)	1727.09 (1072.07-2568.73)	-0.1 (-0.11--0.08)
Portugal	95851 (59005-143811)	1380.39 (848.71-2072.24)	106330 (66092-159068)	1356.43 (839.56-2028.04)	-0.11 (-0.13--0.08)
Puerto Rico	18262 (11131-27731)	817.31 (498.16-1241.14)	19158 (11589-28853)	812.09 (489.46-1227.6)	-0.03 (-0.04--0.02)
Qatar	3041 (1852-4599)	1025.14 (627.53-1556.26)	25511 (15525-38765)	1044.68 (640.24-1583.78)	-0.01 (-0.03-0.01)
Republic of Korea	391521 (239250-589288)	1347.61 (824.5-2027.11)	527694 (322568-789164)	1257.68 (764.93-1882.97)	-0.19 (-0.22--0.17)
Republic of Moldova	40335 (24942-60471)	1389.01 (859.27-2084.52)	39312 (24302-59080)	1361.73 (838.47-2046.83)	-0.09 (-0.1--0.07)
Romania	299316 (186767-445673)	1849.72 (1153.08-2758.4)	243746 (150705-364082)	1751.54 (1079.72-2620.44)	-0.21 (-0.24--0.19)
Russian Federation	1538984 (954261-2290316)	1420.24 (879.88-2115.92)	1519972 (944747-2261716)	1366.83 (846.72-2035.65)	0.01 (-0.03-0.05)
Rwanda	31802 (19434-48034)	1070.5 (656.41-1615.56)	70014 (42558-105729)	1034.08 (630.19-1557.84)	-0.12 (-0.14--0.11)
Saint Kitts and Nevis	171 (103-259)	819.37 (492.62-1238.92)	373 (228-567)	797.24 (485.49-1210.3)	-0.09 (-0.1--0.09)
Saint Lucia	587 (356-896)	866.93 (528.36-1320.44)	1132 (695-1721)	825.02 (504.64-1253)	-0.16 (-0.16--0.15)

Saint Vincent and the Grenadines	438 (264-666)	813.87 (493.35-1236.82)	647 (395-981)	799.55 (486.93-1214.57)	-0.04 (-0.04--0.03)
Samoa	699 (421-1066)	912.02 (551.15-1387.58)	986 (600-1495)	857.24 (521.91-1298.78)	-0.26 (-0.29--0.22)
San Marino	215 (132-325)	1288.07 (787.96-1950.31)	304 (188-457)	1239.85 (760.7-1867.82)	-0.1 (-0.11--0.1)
Sao Tome and Principe	412 (247-622)	819.61 (493.75-1237.85)	921 (553-1400)	794.04 (479.33-1206.86)	-0.11 (-0.12--0.09)
Saudi Arabia	83917 (51327-126689)	1044.4 (640.82-1577.06)	310780 (189396-472250)	1071.15 (653.1-1627.25)	0.11 (0.1-0.12)
Senegal	26168 (16146-39256)	850.16 (528.04-1273.76)	62528 (37758-94562)	822.09 (499.75-1241.67)	-0.17 (-0.21--0.14)
Serbia	121317 (75184-180871)	1743.5 (1079.93-2600.5)	114799 (70953-172178)	1747.25 (1077.99-2621.62)	0.01 (0.01-0.02)
Seychelles	290 (173-442)	738.95 (443.9-1124.3)	554 (335-836)	697.2 (421.7-1054.43)	-0.19 (-0.2--0.19)
Sierra Leone	17383 (10465-26385)	941.54 (570.41-1427.24)	37033 (22499-55786)	887.54 (542.53-1335.34)	-0.19 (-0.21--0.17)
Singapore	24112 (14695-36275)	1129.1 (689.05-1698.83)	48024 (29426-72719)	1036.34 (633.78-1570.66)	-0.13 (-0.2--0.07)
Slovakia	61848 (38841-91293)	1782.87 (1120.63-2633.91)	70547 (43612-105388)	1712.57 (1054.39-2559.87)	-0.1 (-0.11--0.09)
Slovenia	22545 (14040-33273)	1596.27 (992.52-2358.64)	24404 (15174-36385)	1572.26 (974.94-2349.85)	-0.05 (-0.07--0.03)
Solomon Islands	1222 (733-1858)	836.82 (503.67-1270.06)	3203 (1948-4839)	886.84 (541.43-1338.52)	0.27 (0.24-0.3)
Somalia	31318 (18920-47371)	965.23 (587.73-1456.41)	83807 (51222-127270)	975.59 (598.95-1476.04)	0.01 (0-0.03)
South Africa	158507 (97135-237978)	864.63 (531.63-1296.01)	289791 (177377-433550)	788.89 (483.5-1179.63)	-0.26 (-0.27--0.25)
South Sudan	23333 (14123-35070)	941.36 (573.86-1414.53)	41350 (25116-62816)	939.05 (571.97-1427.73)	-0.01 (-0.03-0)
Spain	289640 (187931-415382)	1093.77 (709.05-1569.2)	339557 (207241-516901)	986.91 (599.81-1499.1)	-0.06 (-0.18-0.05)
Sri Lanka	73078 (44731-110416)	743.72 (457.17-1124.16)	114630 (69395-174218)	736.31 (444.91-1120.31)	-0.04 (-0.05--0.02)
Sudan	105829 (64197-160099)	1129.83 (688.45-1707.62)	260796 (158987-391168)	1123.25 (686.43-1685.17)	0 (-0.01-0)
Suriname	1773 (1063-2686)	815.42 (490.63-1232.75)	3293 (2015-4958)	822.24 (502.69-1237.68)	0.05 (0.04-0.07)
Sweden	41443 (26330-60660)	716.56 (453.44-1048.78)	66053 (39221-102227)	934.48 (553.95-1445.6)	0.86 (0.64-1.09)
Switzerland	75678 (50155-107279)	1551.84 (1026.89-2201.27)	89018 (54826-134139)	1347.2 (827.1-2033.13)	-0.22 (-0.38--0.05)
Syrian Arab Republic	65761 (40107-99539)	1150.1 (702.06-1739.16)	106952 (65523-160392)	1135.85 (692.65-1706.61)	-0.05 (-0.06--0.03)
Taiwan (Province of China)	121421 (81821-169022)	943.48 (636.21-1311.38)	211457 (149397-284655)	1081.27 (762.08-1458.57)	0.52 (0.43-0.59)
Tajikistan	28124 (17300-42401)	1159.31 (715.06-1748.21)	65182 (39677-97732)	1121.51 (683.57-1680.73)	-0.09 (-0.1--0.09)
Thailand	216020 (132217-325846)	637 (391.03-960.17)	375372 (226437-572079)	680.39 (409.61-1036.52)	0.28 (0.19-0.38)

Timor-Leste	2744 (1650-4178)	739.74 (447.98-1126.54)	4894 (2949-7439)	704.97 (426.58-1068.13)	-0.17 (-0.2--0.14)
Togo	13793 (8335-20918)	953.66 (580.25-1445.75)	37950 (23025-57291)	897.37 (547.61-1353.72)	-0.23 (-0.28--0.18)
Tokelau	7 (4-11)	859.13 (522.74-1311.3)	7 (4-11)	837.56 (510.74-1259.31)	-0.07 (-0.09--0.06)
Tonga	416 (252-628)	903.45 (548.85-1362.47)	497 (302-754)	868.35 (528.95-1316.81)	-0.15 (-0.17--0.14)
Trinidad and Tobago	5569 (3364-8419)	831.13 (503.91-1256.9)	8354 (5125-12580)	818.32 (500.27-1234.45)	-0.03 (-0.04--0.02)
Tunisia	49681 (30807-74235)	1112.66 (691.51-1660.52)	94048 (57586-140744)	1132.86 (692.65-1696.82)	0.04 (0.02-0.07)
Turkey	384730 (247081-558617)	1190.1 (766.06-1727.88)	685489 (420276-1040429)	1169.5 (717.01-1775.03)	0.05 (-0.05-0.14)
Turkmenistan	21037 (12867-31836)	1190.01 (731.09-1798.13)	38215 (23282-57662)	1155.43 (704.36-1743.35)	-0.05 (-0.07--0.04)
Tuvalu	46 (28-70)	876.08 (529.58-1330.05)	63 (38-96)	847.76 (514.81-1285.19)	-0.07 (-0.08--0.05)
Uganda	68349 (41787-103129)	1010.85 (620.83-1518.91)	177001 (107668-266818)	984.6 (601.85-1481.75)	-0.08 (-0.09--0.06)
Ukraine	627371 (388771-933073)	1637.07 (1012.62-2435.89)	539788 (337745-805888)	1580.62 (984.28-2364.11)	-0.07 (-0.09--0.04)
United Arab Emirates	10929 (6628-16562)	922.46 (562.86-1397.54)	84942 (51485-128424)	969.29 (588.25-1464.75)	0.11 (0.05-0.17)
United Kingdom	515216 (320206-766431)	1330.51 (826.02-1979.54)	608929 (376686-908851)	1268.7 (783.06-1894.86)	-0.06 (-0.09--0.02)
United Republic of Tanzania	105228 (63485-159626)	983.24 (597.15-1488.46)	262598 (160438-396364)	961.22 (591.18-1449.28)	-0.03 (-0.05--0.02)
United States of America	557 (335-850)	1635.44 (1030.34-2404.4)	3402390 (2289772-4729577)	1463.5 (982.78-2036.04)	-0.17 (-0.25--0.09)
United States Virgin Islands	2758928 (1737010-4054092)	824.67 (495.64-1258.38)	513 (313-775)	816.68 (496.64-1237.93)	-0.01 (-0.03-0)
Uruguay	25214 (15735-37761)	1268.31 (790.73-1899.83)	31836 (19588-47891)	1364.97 (838.53-2054.73)	0.21 (0.15-0.27)
Uzbekistan	119890 (72914-181603)	1205.29 (737.55-1821.07)	266823 (163590-400151)	1193.07 (731.96-1789.2)	0.03 (0.01-0.05)
Vanuatu	627 (382-950)	918.52 (562.65-1393.72)	1560 (955-2363)	929.36 (571.29-1407.68)	0.04 (0.01-0.08)
Venezuela (Bolivarian Republic of)	99606 (60137-150230)	1003.15 (607.69-1512.56)	176465 (107302-268704)	965 (585.92-1469.57)	-0.14 (-0.15--0.12)
Viet Nam	254497 (154137-379906)	777.68 (472.63-1160.3)	558705 (334856-844039)	778.84 (466.48-1177.44)	0.05 (0.03-0.08)
Yemen	64446 (39112-97404)	1152.49 (702.97-1739.88)	190765 (116382-286467)	1109.91 (679.39-1664.55)	-0.14 (-0.15--0.13)
Zambia	25439 (15616-38167)	800.03 (493.43-1197.61)	77792 (47111-118000)	877.84 (535.39-1328.28)	0.16 (0.09-0.22)
Zimbabwe	35905 (22020-53572)	869.88 (536.87-1297.2)	69064 (42175-105038)	933.68 (572.61-1420.18)	0.28 (0.27-0.3)

Abbreviations: ASDR, age-standardized DALYs rate; EAPC, estimated annual percentage change; UI, uncertainty interval; CI, confidence interval.

Table S5. The APC and AAPC in case number of LBP in working-age group of incidence, prevalence and DALYs from 1990 to 2021.

Incidence			Prevalence			DALYs		
**Range**	**APC (95% CI)**	**P**	**Range**	**APC (95% CI)**	**P**	**Range**	**APC (95% CI)**	**P**
1990-1993	0.68 (0.55-0.8)	< 0.001	1990-1993	0.63 (0.52-0.73)	< 0.001	1990-1993	0.64 (0.54-0.74)	< 0.001
1993-1999	1.18 (1.13-1.24)	< 0.001	1993-1999	1.16 (1.12-1.21)	< 0.001	1993-1999	1.17 (1.13-1.21)	< 0.001
1999-2011	1.76 (1.74-1.77)	< 0.001	1999-2011	1.76 (1.75-1.78)	< 0.001	1999-2011	1.78 (1.76-1.79)	< 0.001
2011-2015	1.54 (1.42-1.67)	< 0.001	2011-2015	1.57 (1.47-1.67)	< 0.001	2011-2015	1.58 (1.48-1.68)	< 0.001
2015-2021	1.07 (1.03-1.11)	< 0.001	2015-2021	1.05 (1.02-1.09)	< 0.001	2015-2021	1.01 (0.98-1.05)	< 0.001
**Range**	**AAPC (95%CI)**	**P**	**Range**	**AAPC (95% CI)**	**P**	**Range**	**AAPC (95% CI)**	**P**
1990-2021	1.38 (1.36-1.4)	< 0.001	1990-2021	1.37 (1.36-1.39)	< 0.001	1990-2021	1.37 (1.36-1.39)	< 0.001

Abbreviations: APC, annual percentage change; AAPC, average annual percentage change; DALYs, disability-adjusted life-years; CI, confidence interval.

Table S6. The APC and AAPC in ASR of LBP in working-age group of incidence, prevalence and DALYs from 1990 to 2021.

Incidence			Prevalence			DALYs		
**Range**	**APC (95% CI)**	**P**	**Range**	**APC (95% CI)**	**P**	**Range**	**APC (95% CI)**	**P**
1990-1993	-1.14 (-1.27--1.01)	< 0.001	1990-1993	-1.22 (-1.36--1.09)	< 0.001	1990-1993	-1.22 (-1.32--1.11)	< 0.001
1993-2000	-0.58 (-0.62--0.53)	< 0.001	1993-2000	-0.59 (-0.64--0.55)	< 0.001	1993-2000	-0.59 (-0.63--0.56)	< 0.001
2000-2004	-0.14 (-0.27--0.01)	0.036	2000-2004	-0.17 (-0.31--0.04)	0.017	2000-2004	-0.16 (-0.27--0.05)	0.007
2004-2010	-0.29 (-0.35--0.24)	< 0.001	2004-2010	-0.32 (-0.38--0.26)	< 0.001	2004-2010	-0.3 (-0.35--0.25)	< 0.001
2010-2025	-0.05 (-0.13-0.04)	0.242	2010-2025	-0.03 (-0.17-0.11)	0.66	2010-2025	-0.05 (-0.12-0.02)	0.145
2015-2021	-0.2 (-0.24--0.15)	< 0.001	2015-2021	-0.21 (-0.25--0.17)	< 0.001	2015-2021	-0.27 (-0.3--0.23)	< 0.001

**Range**	**AAPC (95%CI)**	**P**	**Range**	**AAPC (95% CI)**	**P**	**Range**	**AAPC (95% CI)**	**P**
1990-2021	-0.36 (-0.39--0.33)	< 0.001	1990-2021	-0.39 (-0.42--0.36)	< 0.001	1990-2021	-0.39 (-0.41--0.37)	< 0.001

Abbreviations: APC, annual percentage change; AAPC, average annual percentage change; ASR, age-standardized rate; DALYs, disability-adjusted life-years; CI, confidence interval.

Table S7. Decomposition analysis of LBP in working-age group change in incidence, prevalence, and DALYs by SDI quintile and sex, 1990 to 2021.

measure	sex	location	Overll difference	Aging	Population	Epidemiological change
Incidence	Both	Global	66624803.67	14279412.45 (21.43%)	70264314.99 (105.46%)	-17918923.77 (-26.9%)
		High SDI	6267956.15	2446664.83 (39.03%)	6873936.49 (109.67%)	-3052645.17 (-48.7%)
		High-middle SDI	7370772.09	4618832.49 (62.66%)	7609903.64 (103.24%)	-4857964.03 (-65.91%)
		Middle SDI	22136448.86	6651040.94 (30.05%)	19021849.31 (85.93%)	-3536441.39 (-15.98%)
		Low-middle SDI	19975295.84	1833292.53 (9.18%)	20091039.77 (100.58%)	-1949036.46 (-9.76%)
		Low SDI	10841173.04	-157362.66 (-1.45%)	11823241.56 (109.06%)	-824705.86 (-7.61%)
Incidence	Female	Global	42467165.08	8946355.96 (21.07%)	43186414.44 (101.69%)	-9665605.32 (-22.76%)
		High SDI	3442006.35	1343197.81 (39.02%)	3669034.55 (106.6%)	-1570226.01 (-45.62%)
		High-middle SDI	4248282.24	2813053.83 (66.22%)	4305680.66 (101.35%)	-2870452.26 (-67.57%)
		Middle SDI	14447211.26	4366970.92 (30.23%)	12147840.5 (84.08%)	-2067600.16 (-14.31%)
		Low-middle SDI	13456881.99	1340645.13 (9.96%)	13165837.93 (97.84%)	-1049601.06 (-7.8%)
		Low SDI	6853216.17	-38914.79 (-0.57%)	7372732.75 (107.58%)	-480601.79 (-7.01%)
Incidence	Male	Global	24157638.58	5414634.13 (22.41%)	27231560.54 (112.72%)	-8488556.09 (-35.14%)
		High SDI	2825949.81	1080533.05 (38.24%)	3125932.27 (110.62%)	-1380515.51 (-48.85%)
		High-middle SDI	3122489.85	1786742.43 (57.22%)	3236999.62 (103.67%)	-1901252.2 (-60.89%)
		Middle SDI	7689237.6	2373533.28 (30.87%)	7016736.77 (91.25%)	-1701032.45 (-22.12%)
		Low-middle SDI	6518413.85	567067.42 (8.7%)	7043349.44 (108.05%)	-1092003.01 (-16.75%)
		Low SDI	3987956.87	-98195.79 (-2.46%)	4468365.78 (112.05%)	-382213.13 (-9.58%)
Prevalence	Both	Global	156203830.9	35850602.69 (22.95%)	165463140.8 (105.93%)	-45109912.63 (-28.88%)
		High SDI	14971634.32	6495952.26 (43.39%)	16592085.77 (110.82%)	-8116403.72 (-54.21%)
		High-middle SDI	17340350.62	11493828.45 (66.28%)	17934474.85 (103.43%)	-12087952.68 (-69.71%)
		Middle SDI	51985042.04	16372971.54 (31.5%)	44197817.77 (85.02%)	-8585747.28 (-16.52%)
		Low-middle SDI	46827340.2	4544071.4 (9.7%)	47068944.43 (100.52%)	-4785675.63 (-10.22%)
		Low SDI	25003103.26	-395033.27 (-1.58%)	27493545.42 (109.96%)	-2095408.88 (-8.38%)
Prevalence	Female	Global	99655975.54	22816031.36 (22.89%)	103064347.43 (103.42%)	-26224403.25 (-26.31%)
		High SDI	7892350.67	3560577.47 (45.11%)	8865090.17 (112.33%)	-4533316.96 (-57.44%)
		High-middle SDI	9701940.14	7084308.31 (73.02%)	10314105.79 (106.31%)	-7696473.96 (-79.33%)
		Middle SDI	34078541.88	10939916.88 (32.1%)	28718809.86 (84.27%)	-5580184.86 (-16.37%)
		Low-middle SDI	31961887.2	3441507 (10.77%)	31434572.12 (98.35%)	-2914191.93 (-9.12%)

		Low SDI	15976735.03	-100626.36 (-0.63%)	17415719.34 (109.01%)	-1338357.95 (-8.38%)
		Global	56547855.31	13269742.11 (23.47%)	62789923.8 (111.04%)	-19511810.59 (-34.5%)
		High SDI	7079283.65	2878176.72 (40.66%)	7534324.06 (106.43%)	-3333217.13 (-47.08%)
Prevalence	Male	High-middle SDI	7638410.47	4353976.2 (57%)	7445511.09 (97.47%)	-4161076.82 (-54.48%)
		Middle SDI	17906500.16	5678995.38 (31.71%)	15841214.1 (88.47%)	-3613709.32 (-20.18%)
		Low-middle SDI	14865453	1321549.07 (8.89%)	15936292.86 (107.2%)	-2392388.94 (-16.09%)
		Low SDI	9026368.23	-236039.03 (-2.61%)	10123343.75 (112.15%)	-860936.48 (-9.54%)
		Global	17736120.15	4096137.66 (23.09%)	18830501.6 (106.17%)	-5190519.11 (-29.27%)
		High SDI	1668318.51	739670.03 (44.34%)	1892066.7 (113.41%)	-963418.22 (-57.75%)
DALYs	Both	High-middle SDI	1996917.54	1315210.65 (65.86%)	2049371.32 (102.63%)	-1367664.43 (-68.49%)
		Middle SDI	5916391.27	1878673.98 (31.75%)	5045661.59 (85.28%)	-1007944.3 (-17.04%)
		Low-middle SDI	5308197.52	516436.66 (9.73%)	5315992.11 (100.15%)	-524231.24 (-9.88%)
		Low SDI	2837546.81	-43541.09 (-1.53%)	3098024.11 (109.18%)	-216936.2 (-7.65%)
		Global	11212677.02	2590214.56 (23.1%)	11647542.7 (103.88%)	-3025080.24 (-26.98%)
		High SDI	866759.64	404503.46 (46.67%)	1004722.16 (115.92%)	-542465.97 (-62.59%)
DALYs	Female	High-middle SDI	1102881.85	806467.08 (73.12%)	1171342.01 (106.21%)	-874927.24 (-79.33%)
		Middle SDI	3849439.24	1246317.05 (32.38%)	3256026.61 (84.58%)	-652904.42 (-16.96%)
		Low-middle SDI	3591557.29	386884.55 (10.77%)	3522736.81 (98.08%)	-318064.08 (-8.86%)
		Low SDI	1797016.05	-10905.95 (-0.61%)	1946299.32 (108.31%)	-138377.31 (-7.7%)
		Global	6523443.12	1531957.18 (23.48%)	7225930.17 (110.77%)	-2234444.23 (-34.25%)
		High SDI	801558.87	328922.19 (41.04%)	866301.4 (108.08%)	-393664.73 (-49.11%)
DALYs	Male	High-middle SDI	894035.69	502643.85 (56.22%)	858722.05 (96.05%)	-467330.21 (-52.27%)
		Middle SDI	2066952.03	659693.66 (31.92%)	1829705.57 (88.52%)	-422447.2 (-20.44%)
		Low-middle SDI	1716640.23	153298.52 (8.93%)	1826252.61 (106.39%)	-262910.9 (-15.32%)
		Low SDI	1040530.75	-26363.35 (-2.53%)	1156641.14 (111.16%)	-89747.04 (-8.63%)

Abbreviations: DALYs, disability-adjusted life-years; SDI, sociodemographic index.

Table S8. The predicted case number and ASR of incidence, prevalence, and DALYs of LBP in working-age group from 2022 to 2050 globally.

Year	Incidence		Prevalence		DALYs	
	**Case number (95%Crl)**	**ASR (95% Crl)**	**Case number (95%Crl)**	**ASR (95% Crl)**	**Case number (95%Crl)**	**ASR (95% Crl)**
**Both**						
2022	195376575 (191346433-199406718)	3669.62 (3593.63-3745.61)	459482761 (449766030-469199491)	8615.2 (8432.54-8797.86)	52213797 (50681184-53746409)	978.97 (949.73-1008.22)
2023	197321109 (192060244-202581973)	3664.4 (3566.2-3762.6)	464056210 (451659161-476453259)	8601.89 (8371.34-8832.45)	52671250 (50487608-54854892)	976.37 (935.05-1017.7)
2024	199514947 (192326717-206703176)	3659.45 (3526.72-3792.17)	469179706 (452749728-485609683)	8588.4 (8286.52-8890.28)	53188775 (49736655-56640894)	973.76 (908.98-1038.54)
2025	201820304 (192268344-211372264)	3655.38 (3481.07-3829.7)	474540373 (452998312-496082435)	8576.45 (8185.58-8967.34)	53730880 (48910820-58550940)	971.31 (881.74-1060.88)
2026	204145908 (191955337-216336479)	3652.05 (3432.33-3871.78)	480008650 (452454845-507562455)	8567.09 (8073.34-9060.84)	54284080 (48289318-60278841)	969.15 (858.98-1079.33)
2027	206241756 (191164840-221318672)	3649.28 (3380.66-3917.9)	484977851 (450611825-519343877)	8559.74 (7950.78-9168.7)	54781419 (47811710-61751129)	967.24 (840.62-1093.85)
2028	207936968 (189742002-226131934)	3647.3 (3326.18-3968.44)	488987189 (447161540-530812838)	8554.28 (7819.72-9288.84)	55171250 (47333393-63009106)	965.54 (824.65-1106.43)
2029	209577751 (188012440-231143062)	3646.39 (3269.05-4023.74)	492835172 (442920524-542749820)	8550.74 (7681.3-9420.17)	55541662 (46868640-64214683)	964.06 (809.75-1118.36)
2030	211361984 (186153534-236570434)	3646.57 (3209.32-4083.82)	497003348 (438349488-555657207)	8549.24 (7536.23-9562.25)	55945455 (46425632-65465278)	962.77 (795.16-1130.4)
2031	213225623 (184096513-242354733)	3647.62 (3146.72-4148.52)	501395554 (433344108-569447001)	8550.32 (7384.97-9715.66)	56373457 (45972987-66773926)	961.79 (780.57-1143.02)
2032	215142292 (181803474-248481110)	3649 (3080.63-4217.37)	505941916 (427809564-584074268)	8552.62 (7226.04-9879.2)	56818815 (45487708-68149922)	960.96 (765.59-1156.33)
2033	216906777 (179114680-254698874)	3650.7 (3011.3-4290.1)	510115361 (421338719-598892002)	8555.52 (7059.59-10051.44)	57223268 (44907133-69539404)	960.21 (749.9-1170.52)
2034	218513398 (176042597-260984200)	3652.83 (2938.99-4366.67)	513874456 (413940513-613808399)	8558.82 (6885.96-10231.69)	57581483 (44222780-70940186)	959.51 (733.35-1185.66)
2035	220084295 (172696473-267472118)	3655.51 (2863.89-4447.14)	517521243 (405884619-629157866)	8562.86 (6705.57-10420.14)	57925820 (43459379-72392260)	958.87 (715.89-1201.85)
2036	221645799 (169093256-274198342)	3658.92 (2786.04-4531.82)	521163579 (397245382-645081776)	8568.7 (6519.07-10618.33)	58269131 (42624547-73913715)	958.45 (697.6-1219.29)
2037	223274259 (165276572-281271946)	3662.87 (2705.08-4620.65)	524986291 (388125982-661846600)	8576.04 (6325.68-10826.4)	58632196 (41729321-75535070)	958.2 (678.41-1237.99)

2038	224957495 (161236899-288678092)	3667.44 (2621.22-4713.67)	528921081 (378474799-679367363)	8584.71 (6125.56-11043.87)	59007352 (40765717-77248987)	958.11 (658.28-1257.94)
2039	226712527 (156984137-296440916)	3672.69 (2534.53-4810.85)	532989014 (368302602-697675425)	8594.56 (5918.72-11270.4)	59395699 (39733534-79057864)	958.16 (637.21-1279.11)
2040	228612905 (152561003-304664807)	3678.59 (2444.96-4912.22)	537373301 (357717673-717028930)	8605.49 (5705.04-11505.94)	59815986 (38644126-80987846)	958.32 (615.17-1301.47)
2041	230606404 (147912758-313300051)	3684.98 (2352.27-5017.7)	541954666 (346593636-737315697)	8617.31 (5484.02-11750.61)	60256787 (37485797-83027778)	958.59 (592.15-1325.03)
2042	232656326 (142994385-322318268)	3691.79 (2256.13-5127.44)	546658364 (334831938-758484790)	8630.14 (5255.16-12005.12)	60710795 (36249497-85172093)	958.98 (568.13-1349.85)
2043	234723287 (137776601-331669972)	3699.31 (2156.77-5241.87)	551381172 (322358877-780403467)	8644.43 (5018.76-12270.11)	61166897 (34927845-87405949)	959.57 (543.13-1376)
2044	236714228 (132200486-341227969)	3707.83 (2054.26-5361.4)	555894301 (309032430-802756172)	8660.66 (4774.97-12546.34)	61599353 (33505715-89692990)	960.36 (517.19-1403.52)
2045	238630563 (126265574-350995553)	3717.46 (1948.53-5486.39)	560201536 (294853524-825549548)	8679.17 (4523.66-12834.68)	62007272 (31983435-92031108)	961.39 (490.31-1432.48)
2046	240492501 (119974859-361010143)	3728.28 (1839.36-5617.19)	564357665 (279833745-848881584)	8700.19 (4264.35-13136.03)	62398073 (30364100-94432045)	962.71 (462.44-1462.97)
2047	242298809 (113314231-371283386)	3740.33 (1726.42-5754.25)	568379712 (263950474-872808949)	8724.18 (3996.39-13451.96)	62774431 (28646690-96902172)	964.35 (433.56-1495.15)
2048	244170286 (106337243-382003330)	3754.03 (1609.7-5898.36)	572548197 (247328308-897768085)	8751.86 (3719.72-13784)	63166727 (26845291-99488164)	966.41 (403.66-1529.17)
2049	246148081 (99046214-393249949)	3769.61 (1489.04-6050.17)	576952261 (229969762-923934760)	8783.7 (3433.93-14133.46)	63583696 (24960448-102206944)	968.92 (372.7-1565.14)
2050	248147952 (91384556-404911349)	3787.18 (1364.16-6210.19)	581383482 (211738769-951028195)	8819.87 (3138.3-14501.45)	64001553 (22977822-105025285)	971.89 (340.63-1603.15)
**Female**						
2022	119809830 (117392864-122226796)	4528.26 (4476.33-4580.2)	284716261 (278768243-290664279)	10736.87 (10609.47-10864.26)	32102261 (31269005-32935516)	1210.61 (1196.23-1224.99)
2023	121056040 (117894251-124217828)	4525.39 (4438.26-4612.53)	287658101 (280025890-295290312)	10727.96 (10516.93-10939.01)	32387449 (31219050-33555848)	1207.95 (1184.19-1231.71)
2024	122443119 (118144080-126742157)	4522.49 (4391.53-4653.45)	290910984 (280760662-301061306)	10717.95 (10402.68-11033.23)	32706038 (30909680-34502396)	1205.17 (1169.73-1240.61)
2025	123894824 (118189194-129600454)	4520.24 (4338.88-4701.58)	294299079 (280958931-307639228)	10708.79 (10273.7-11143.87)	33037884 (30558772-35516996)	1202.46 (1153.62-1251.29)
2026	125364497	4518.6	297775170	10702.5	33378742	1200.07

	(118053117-132675877)	(4281.07-4756.12)	(280671415-314878924)	(10133.79-11271.21)	(30292661-36464824)	(1136.33-1263.8)
2027	126695051	4517.2	300949400	10697.95	33686043	1197.9
	(117595180-135794923)	(4217.94-4816.45)	(279567527-322331274)	(9982.36-11413.53)	(30067349-37304738)	(1117.83-1277.97)
2028	127775394	4516.32	303518734	10694.74	33926002	1195.9
	(116727098-138823690)	(4150.56-4882.07)	(277446630-329590838)	(9820.96-11568.53)	(29804566-38047438)	(1098.26-1293.53)
2029	128819748	4516.14	305980010	10692.5	34152867	1193.98
	(115658912-141980584)	(4079.57-4952.72)	(274820392-337139628)	(9650.4-11734.6)	(29523756-38781977)	(1077.72-1310.26)
2030	129951920	4516.63	308635368	10691.08	34399315	1192.14
	(114506109-145397731)	(4005.18-5028.08)	(271979267-345291468)	(9471.19-11910.97)	(29237529-39561101)	(1056.23-1328.05)
2031	131136046	4517.49	311447147	10691.58	34662540	1190.51
	(113231338-149040754)	(3927.18-5107.81)	(268877724-354016570)	(9284.33-12098.82)	(28934135-40390945)	(1033.95-1347.06)
2032	132352557	4517.89	314361763	10691.76	34937364	1188.87
	(111808469-152896645)	(3844.68-5191.1)	(265445615-363277911)	(9087.52-12296)	(28601567-41273160)	(1010.65-1367.09)
2033	133469121	4517.78	317031696	10690.58	35185507	1187.1
	(110135282-156802960)	(3758.08-5277.49)	(261413996-372649396)	(8880.85-12500.3)	(28202907-42168106)	(986.35-1387.87)
2034	134478927	4517.19	319419839	10687.47	35402197	1185.13
	(108217414-160740440)	(3667.68-5366.72)	(256780284-382059395)	(8664.59-12710.35)	(27734128-43070266)	(961.04-1409.23)
2035	135458608	4516.22	321718234	10682.67	35607701	1182.95
	(106123787-164793429)	(3573.73-5458.7)	(251716827-391719642)	(8439.39-12925.97)	(27212623-44002778)	(934.79-1431.11)
2036	136428139	4515.09	324015142	10677.94	35812665	1180.79
	(103868016-168988261)	(3476.43-5553.75)	(246286881-401743404)	(8206.63-13149.26)	(26646622-44978707)	(907.79-1453.78)
2037	137434171	4513.51	326422152	10672.65	36029666	1178.58
	(101476232-173392111)	(3375.4-5651.62)	(240550259-412294044)	(7965.49-13379.82)	(26043441-46015892)	(879.95-1477.22)
2038	138470441	4511.65	328892236	10666.52	36253400	1176.32
	(98943850-177997032)	(3271.05-5752.25)	(234472144-423312328)	(7716.42-13616.63)	(25398769-47108032)	(851.33-1501.31)
2039	139545862	4509.51	331432873	10659.18	36483673	1173.93
	(96275982-182815742)	(3163.57-5855.44)	(228055745-434810001)	(7459.65-13858.7)	(24712491-48254854)	(821.95-1525.92)
2040	140703169	4506.86	334156139	10650.23	36731683	1171.35
	(93497326-187909011)	(3052.94-5960.8)	(221367654-446944623)	(7195.23-14105.24)	(23991834-49471531)	(791.78-1550.92)
2041	141906027	4503.38	336985193	10639.28	36990436	1168.57
	(90571887-193240168)	(2938.91-6067.85)	(214328332-459642055)	(6922.89-14355.69)	(23229991-50750882)	(760.84-1576.31)
2042	143131870	4498.92	339869423	10626.3	37255063	1165.61
	(87472359-198791382)	(2821.32-6176.52)	(206873832-472865014)	(6642.4-14610.19)	(22421219-52088908)	(729.11-1602.1)
2043	144364372	4494.02	342755544	10611.95	37519772	1162.53
	(84186578-204542166)	(2700.76-6287.27)	(198966959-486544129)	(6354.8-14869.1)	(21561914-53477631)	(696.7-1628.34)
2044	145546031	4488.95	345499836	10596.76	37768400	1159.36

	(80678705-210413357)	(2577.54-6400.36)	(190519971-500479700)	(6060.8-15132.73)	(20642700-54894101)	(663.68-1655.04)
2045	146673084	4483.74	348098705	10580.95	37999706	1156.11
	(76946303-216399865)	(2451.75-6515.72)	(181531234-514666175)	(5760.73-15401.16)	(19663482-56335930)	(630.07-1682.16)
2046	147754104	4478.25	350584177	10564.55	38218340	1152.82
	(72989866-222518341)	(2323.3-6633.2)	(172008011-529160343)	(5454.57-15674.54)	(18626324-57810355)	(595.88-1709.76)
2047	148788066	4472.51	352961008	10547.9	38425392	1149.53
	(68801307-228774824)	(2192.08-6752.93)	(161933897-543988119)	(5142.21-15953.6)	(17530294-59320490)	(561.13-1737.93)
2048	149858793	4467.09	355417713	10531.85	38640924	1146.32
	(64418947-235298640)	(2058.55-6875.64)	(151394559-559440867)	(4824.42-16239.29)	(16384844-60897004)	(525.87-1766.77)
2049	150995578	4462.28	358020193	10516.9	38871298	1143.23
	(59846157-242144999)	(1922.88-7001.68)	(140396710-575643677)	(4501.6-16532.19)	(15190745-62551851)	(490.15-1796.31)
2050	152141553	4458.01	360634610	10503.02	39101360	1140.24
	(55046398-249236707)	(1785.04-7130.99)	(128854694-592414527)	(4173.76-16832.27)	(13938931-64263790)	(453.98-1826.51)
**Male**						
2022	75566745	2822.66	174766500	6519.84	20111536	750.19
	(73953569-77179922)	(2789.86-2855.46)	(170997788-178535212)	(6441.54-6598.14)	(19412179-20810893)	(741.14-759.25)
2023	76265069	2815.61	176398109	6503.48	20283801	747.8
	(74165994-78364145)	(2761.61-2869.62)	(171633271-181162948)	(6376.06-6630.9)	(19268558-21299044)	(733.07-762.53)
2024	77071828	2809.05	178268722	6487.72	20482737	745.48
	(74182637-79961019)	(2728.63-2889.47)	(171989066-184548377)	(6299.02-6676.43)	(18826976-22138499)	(723.68-767.29)
2025	77925480	2803.3	180241294	6473.43	20692996	743.33
	(74079150-81771810)	(2692.54-2914.07)	(172039381-188443207)	(6214.32-6732.55)	(18352048-23033944)	(713.4-773.25)
2026	78781411	2797.96	182233481	6460.33	20905337	741.31
	(73902220-83660602)	(2653.44-2942.48)	(171783430-192683531)	(6122.86-6797.79)	(17996657-23814017)	(702.37-780.25)
2027	79546704	2792.93	184028451	6448.19	21095376	739.4
	(73569660-85523749)	(2611.42-2974.43)	(171044298-197012603)	(6024.89-6871.48)	(17744360-24446392)	(690.59-788.21)
2028	80161574	2788.39	185468455	6437.05	21245248	737.61
	(73014904-87308245)	(2567.14-3009.65)	(169714910-201222000)	(5921.54-6952.55)	(17528827-24961668)	(678.22-797.01)
2029	80758003	2784.59	186855162	6427.26	21388795	735.98
	(72353528-89162478)	(2521.09-3048.1)	(168100132-205610192)	(5813.8-7040.71)	(17344885-25432706)	(665.34-806.61)
2030	81410064	2781.6	188367980	6419.01	21546140	734.51
	(71647425-91172703)	(2473.48-3089.74)	(166370221-210365739)	(5702.19-7135.83)	(17188103-25904177)	(652.03-816.98)
2031	82089577	2779.16	189948407	6412.02	21710916	733.18
	(70865175-93313979)	(2424.08-3134.24)	(164466383-215430431)	(5586.48-7237.57)	(17038852-26382981)	(638.27-828.1)
2032	82789735	2776.89	191580153	6405.57	21881451	731.92
	(69995004-95584465)	(2372.5-3181.28)	(162363949-220796357)	(5465.87-7345.26)	(16886141-26876762)	(623.96-839.88)

2033	83437656	2774.78	193083664	6399.29	22037761	730.68
	(68979398-97895914)	(2318.94-3230.62)	(159924723-226242606)	(5340.59-7457.99)	(16704226-27371297)	(609.14-852.22)
2034	84034471	2772.93	194454617	6393.23	22179286	729.47
	(67825183-100243760)	(2263.64-3282.23)	(157160230-231749003)	(5211.02-7575.45)	(16488652-27869921)	(593.85-865.09)
2035	84625687	2771.44	195803008	6387.65	22318119	728.31
	(66572685-102678689)	(2206.73-3336.16)	(154167792-237438224)	(5077.56-7697.74)	(16246756-28389482)	(578.14-878.49)
2036	85217660	2770.32	197148436	6382.64	22456466	727.22
	(65225239-105210081)	(2148.2-3392.43)	(150958501-243338372)	(4940.22-7825.05)	(15977924-28935007)	(562-892.45)
2037	85840087	2769.31	198564139	6377.95	22602530	726.16
	(63800340-107879835)	(2087.76-3450.86)	(147575723-249552555)	(4798.62-7957.27)	(15685880-29519179)	(545.4-906.94)
2038	86487054	2768.42	200028845	6373.36	22753952	725.13
	(62293049-110681059)	(2025.54-3511.3)	(144002654-256055035)	(4652.93-8093.8)	(15366949-30140955)	(528.35-921.92)
2039	87166664	2767.67	201556140	6368.8	22912026	724.11
	(60708155-113625174)	(1961.65-3573.69)	(140246858-262865423)	(4503.31-8234.3)	(15021043-30803009)	(510.89-937.33)
2040	87909736	2767.13	203217163	6364.34	23084303	723.09
	(59063677-116755796)	(1896.18-3638.09)	(136350019-270084307)	(4349.92-8378.76)	(14652291-31516314)	(493.02-953.16)
2041	88700377	2766.73	204969473	6359.65	23266351	722.05
	(57340871-120059883)	(1829.02-3704.44)	(132265304-277673642)	(4192.49-8526.81)	(14255806-32276896)	(474.71-969.39)
2042	89524456	2766.28	206788941	6354.68	23455732	720.99
	(55522026-123526886)	(1759.99-3772.58)	(127958106-285619777)	(4030.83-8678.54)	(13828278-33083186)	(455.96-986.01)
2043	90358915	2765.79	208625628	6349.46	23647125	719.9
	(53590023-127127806)	(1689.17-3842.42)	(123391918-293859338)	(3865.21-8833.71)	(13365932-33928318)	(436.77-1003.03)
2044	91168197	2765.37	210394466	6344.15	23830952	718.82
	(51521782-130814612)	(1616.71-3914.04)	(118512459-302276472)	(3695.9-8992.39)	(12863016-34798889)	(417.21-1020.42)
2045	91957479	2765.21	212102831	6339.06	24007566	717.76
	(49319271-134595688)	(1542.72-3987.7)	(113322290-310883373)	(3523.17-9154.96)	(12319953-35695178)	(397.28-1038.23)
2046	92738397	2765.4	213773488	6334.24	24179733	716.72
	(46984993-138491802)	(1467.22-4063.58)	(107825734-319721241)	(3346.93-9321.54)	(11737776-36621690)	(376.98-1056.47)
2047	93510743	2765.87	215418703	6329.88	24349039	715.74
	(44512924-142508563)	(1390.06-4141.67)	(102016577-328820830)	(3167.13-9492.62)	(11116397-37581682)	(356.3-1075.19)
2048	94311493	2766.71	217130484	6326.29	24525804	714.85
	(41918296-146704690)	(1311.35-4222.07)	(95933750-338327218)	(2984.04-9668.54)	(10460447-38591161)	(335.28-1094.43)
2049	95152503	2767.98	218932068	6323.57	24712398	714.06
	(39200056-151104951)	(1231.09-4304.85)	(89573052-348291083)	(2797.75-9849.39)	(9769703-39655092)	(313.92-1114.21)
2050	96006400	2769.79	220748872	6321.76	24900193	713.37
	(36338158-155674642)	(1149.35-4390.24)	(82884075-358613668)	(2608.23-10035.28)	(9038891-40761495)	(292.22-1134.51)

Abbreviations: ASR, age-standardized rate; DALYs, disability-adjusted life-years; CrI, credible interval.

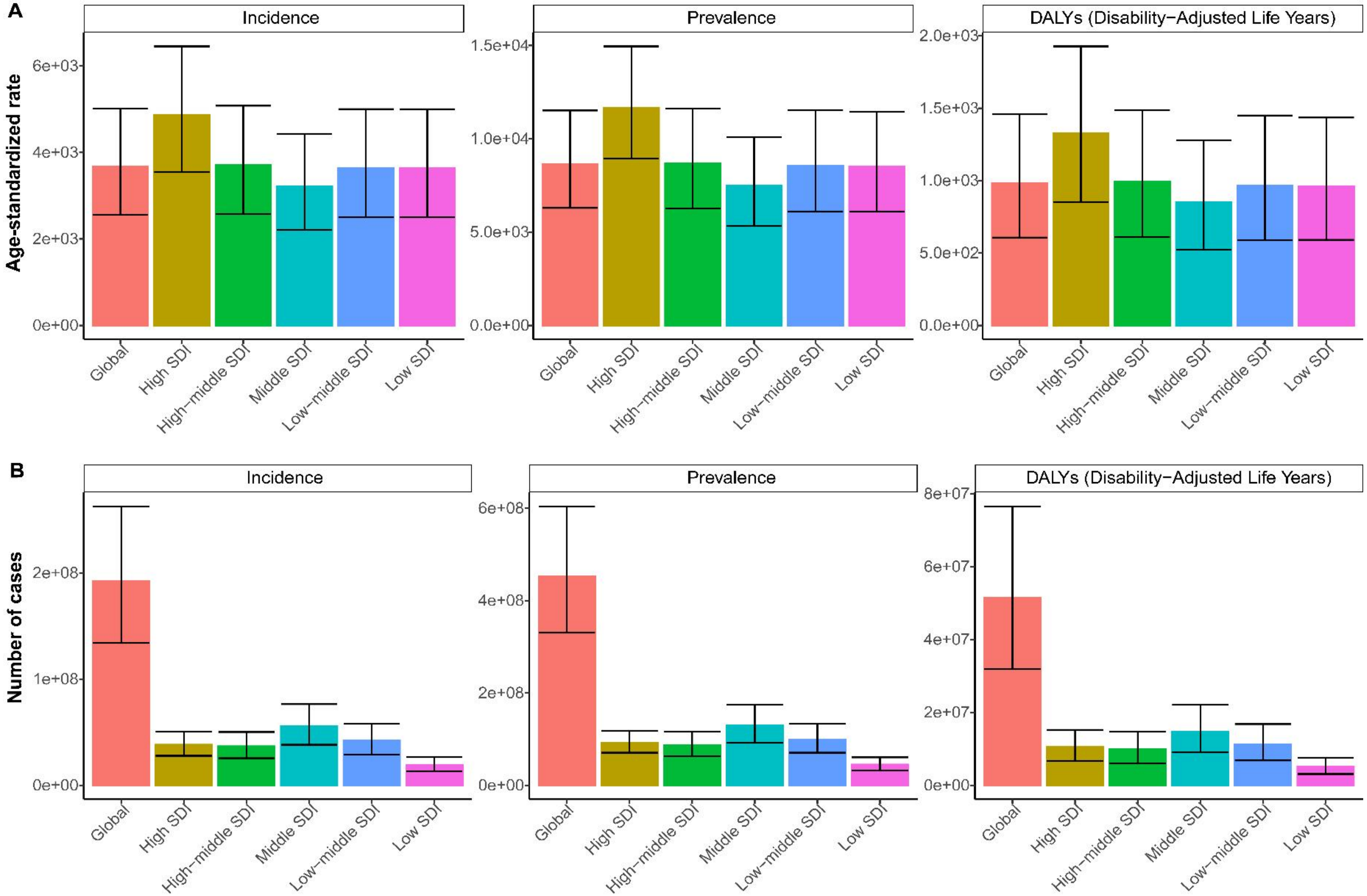

Figure S1. Age-standardized rate and number of incidence, prevalence, and DALYs of LBP in working-age population by Global and SDI, 2021.

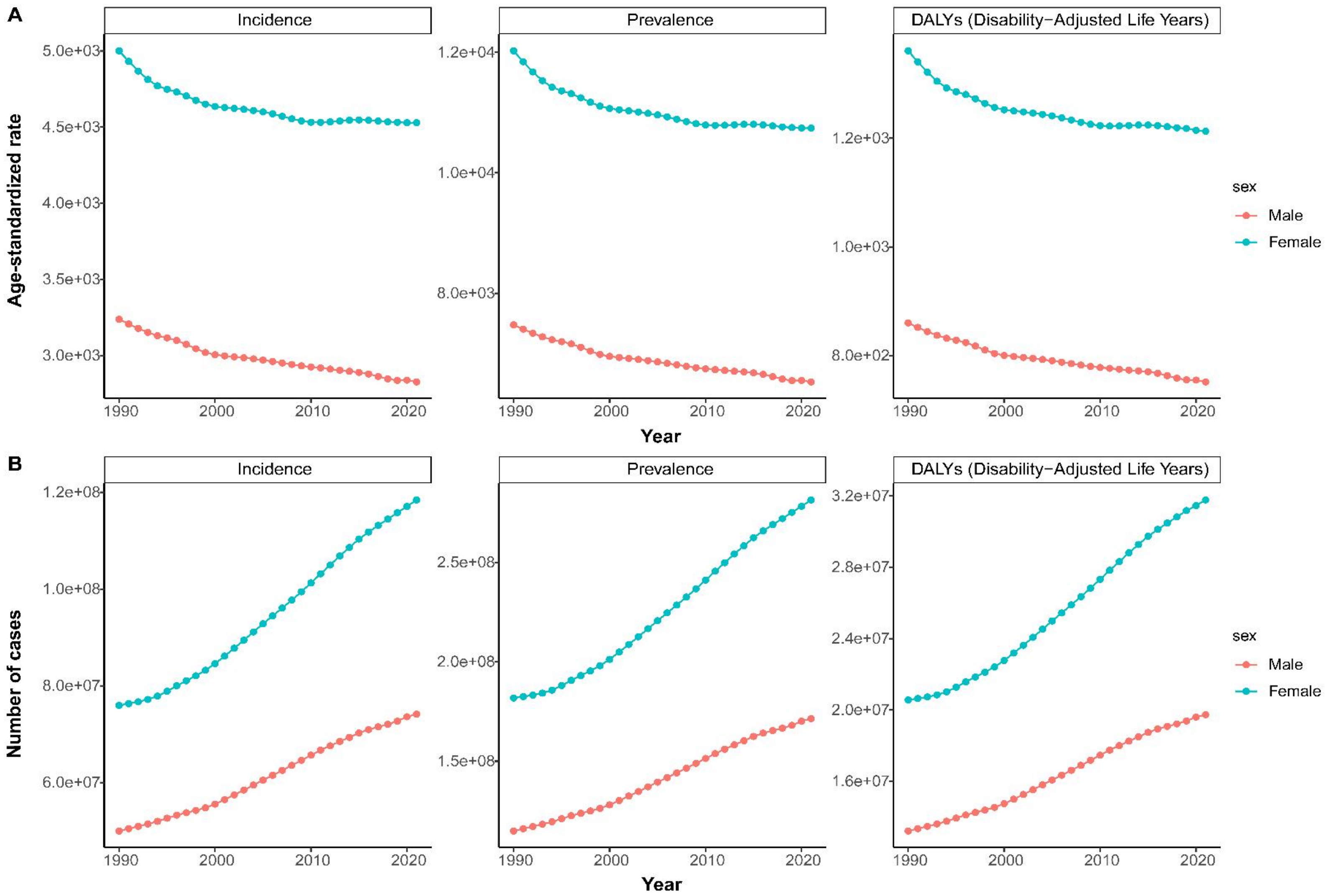

Figure S2. Age-standardized rate and number of incidence, prevalence, and DALYs change curves for LBP in working-age group by sex from 1990 to 2021.

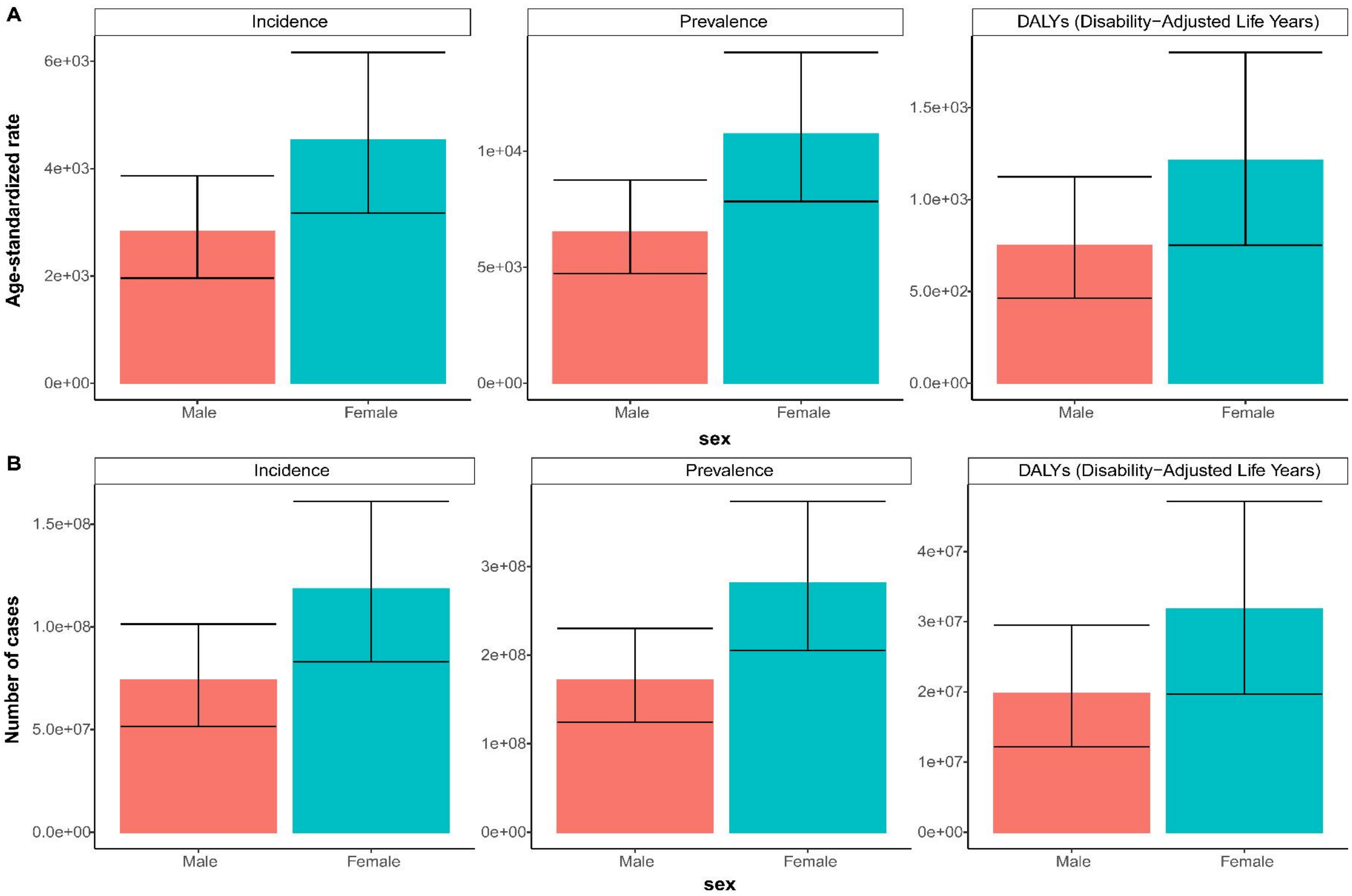

Figure S3. Age-standardized rate and number of incidence, prevalence, and DALYs of LBP in working-age group by sex, 2021.

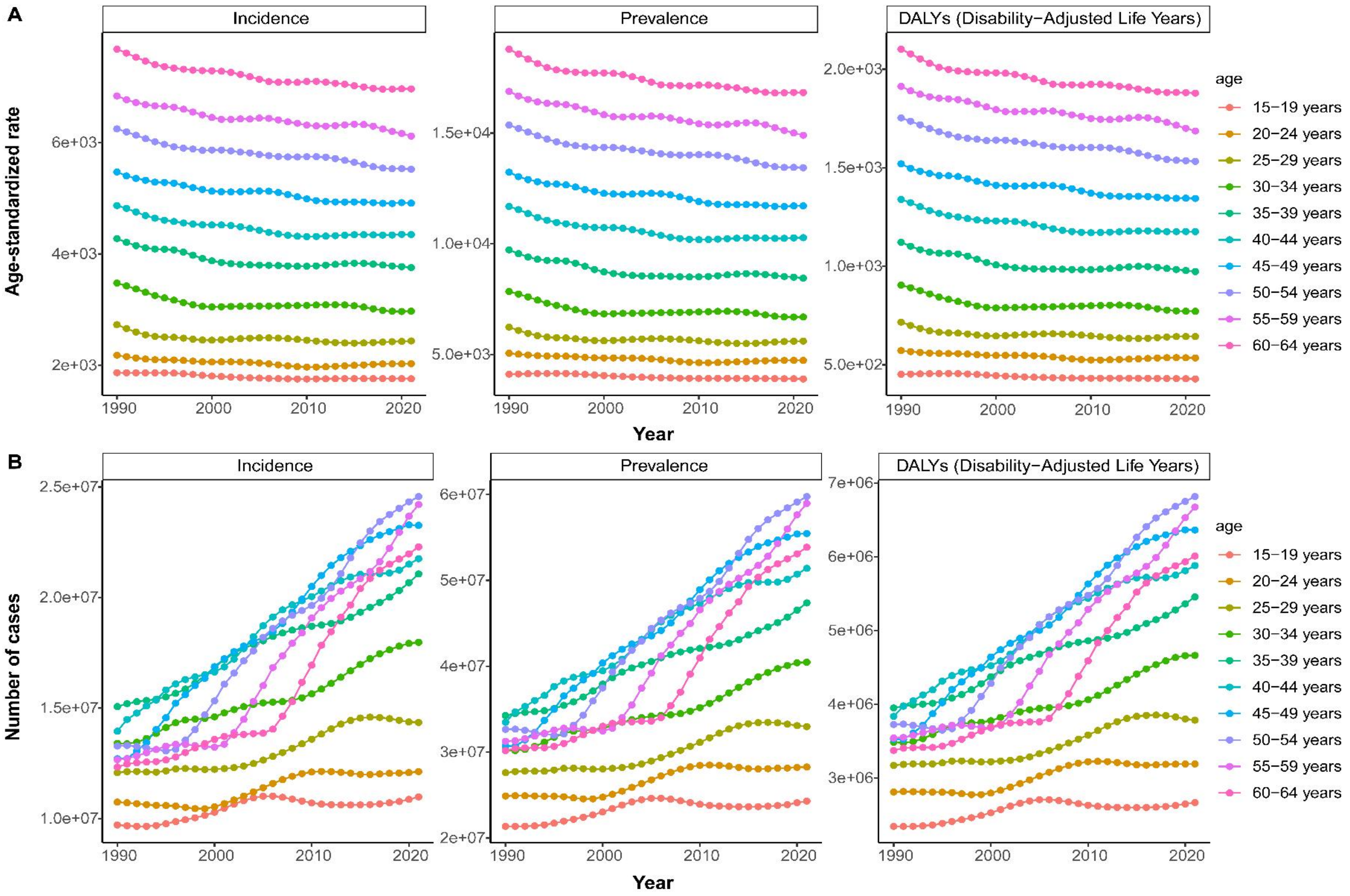

Figure S4. Age-standardized rate and number of incidence, prevalence, and DALYs change curves for LBP in working-age group by sex from 1990 to 2021.

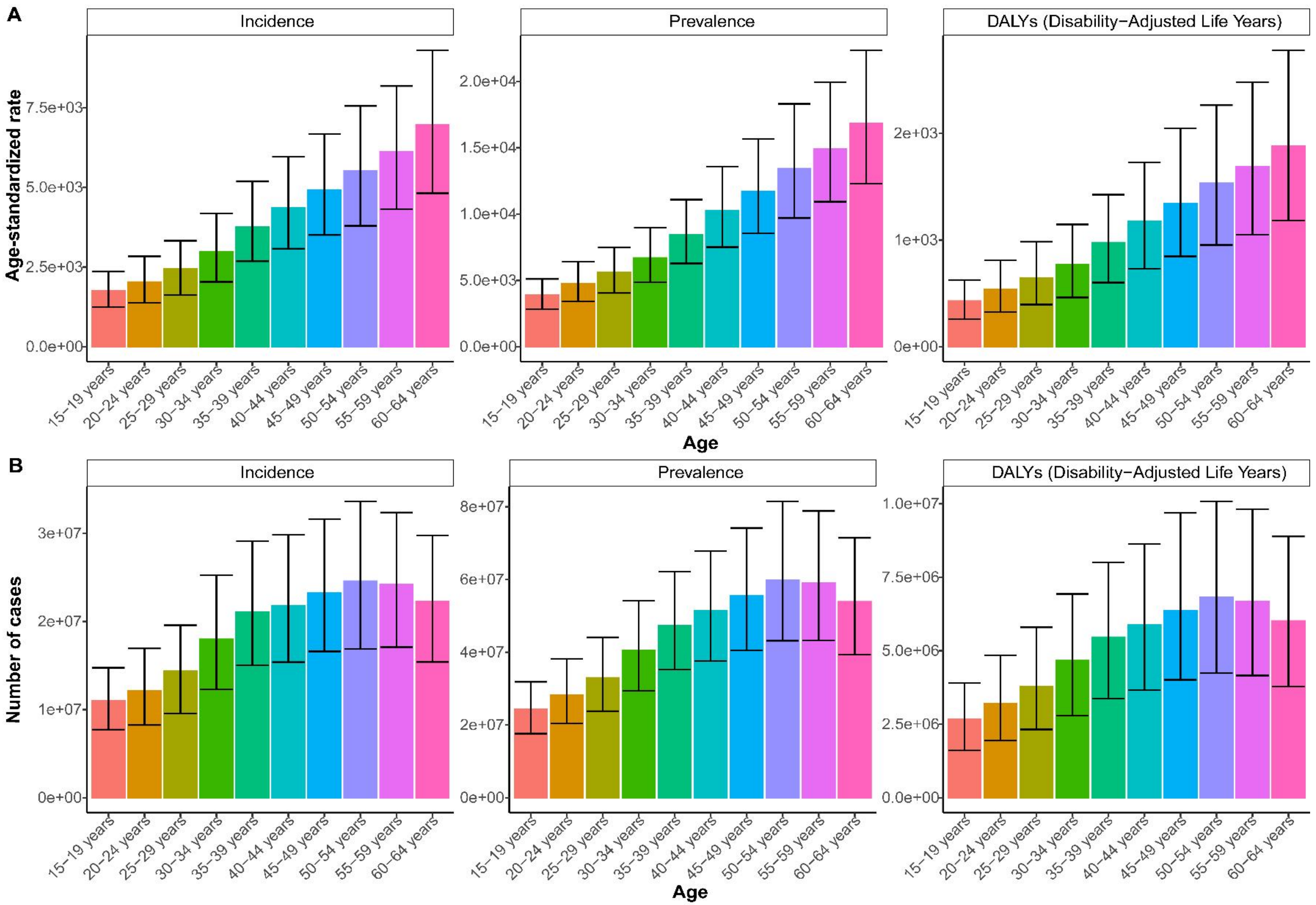

Figure S5. Age-standardized rate and number of incidence, prevalence, and DALYs of LBP in working-age group by age, 2021.

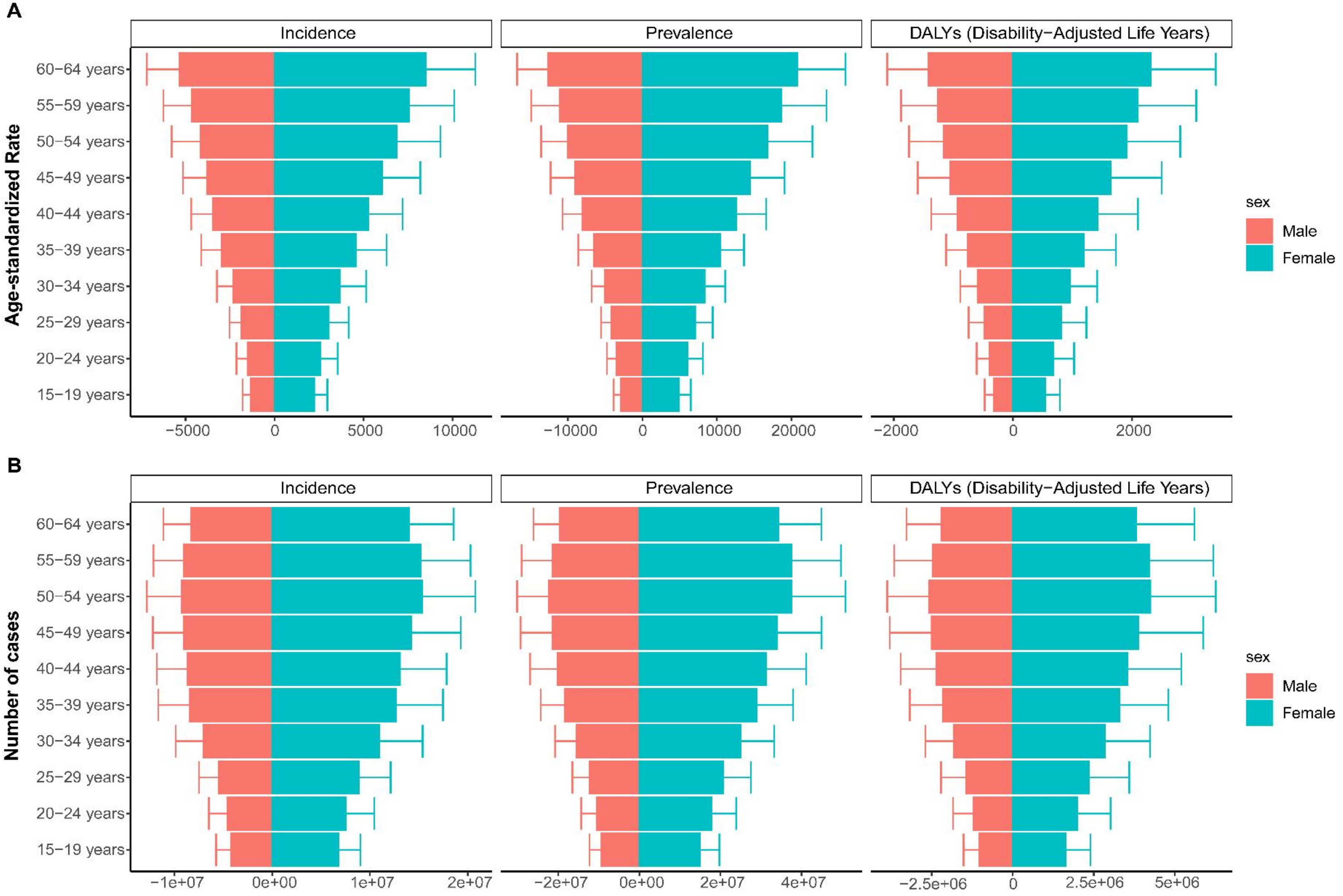

Figure S6. Age- standardized rate and number of incidence, prevalence, and DALYs of LBP in working-age group by sex, 2021.
